# Robust finite-time anti-swing control for quadrotor slung-load system based on compensation function observer

**DOI:** 10.1371/journal.pone.0331662

**Published:** 2026-04-24

**Authors:** Xiaoning Yu, Liaozhang Li, Kun Yan, Jiayong Fang, Peng Zhang, Bo Cheng

**Affiliations:** 1 College of Electronic Information Engineering, Xi’an Technological University, Xi’an, China; 2 College of Equipment Management and UAV Engineering, Air Force Engineering University, Xi’an, China; 3 Unmanned System Research Institute, Northwestern Polytechnical University, Xi’an, China; 4 Nation Key Laboratory of Unmanned Aerial Vehicle Technology, Northwestern Polytechnical University, Xi’an, China; University of Liverpool, UNITED KINGDOM OF GREAT BRITAIN AND NORTHERN IRELAND

## Abstract

In this work, a robust finite-time anti-swing controller is proposed for the quadrotor slung-load system subject to external disturbances. To stabilize the swing angles of the payload, an energy function incorporating both kinetic energy and potential energy is constructed such that the swing angles gradually converge to zero. Since the swing angle information is integrated into the energy function, the global convergence of the quadrotor slung-load system is improved. Meanwhile, a novel compensation function observer is designed to suppress unknown disturbances, thereby enhancing the robustness of the closed-loop system. Compared with the traditional extended state observer method, the developed compensation function observer achieves a faster convergence rate and higher observation accuracy with a smaller observer gain. To further improve the fast convergence performance of the entire closed-loop system, the finite-time control technique is adopted to design the robust anti-swing flight control scheme based on the Lyapunov stability theory and backstepping approach. This control scheme guarantees that all error signals of the quadrotor slung-load system are uniformly ultimately bounded. Finally, simulation verification is performed and comparison results are provided to illustrate the superior effectiveness of the presented control algorithm.

## 1 Introduction

In recent years, the quadrotor slung-load system (QSLS), which consists of a quadrotor unmanned aerial vehicle (UAV), a rigid string and a payload, has demonstrated significant application potential and scientific value in many areas, such as cargo transportation, cooperative positioning, forest-fire prevention and environmental monitoring [1–4]. However, the quadrotor UAV is a unique system characterized by underactuation and multivariability. When the payload is attached to the quadrotor by a rigid string, the complexity of the QSLS increases and the controllability needs to be enhanced [5]. Hence, anti-swing control has become a highly challenging research topic for improving the safety of the coupled system. In what follows, we briefly review representative anti-swing control strategies for QSLS and highlight the remaining challenges.

Recent research has made significant progress in the swing angle stabilization of QSLS, with various effective control strategies having emerged in the existing literature, such as the trajectory planning method [6–8], the state-dependent Riccati equation method [9], the nonlinear geometric control method [10], the adaptive control method [11], and the energy function-based control method [12–16]. Among these methods, the energy function method has been extensively employed owing to its straightforward design process and effective anti-swing capability. In [12], an energy function-based nonlinear control approach was introduced to overcome the inherent underactuation and strong coupling characteristics of double-pendulum-load quadrotor systems. In [13], a dual-loop nonlinear control scheme integrating energy analysis with barrier Lyapunov functions was presented to achieve coordinated trajectory tracking and swing suppression. In [14], an asymptotic stabilization control method based on the energy function technique was established for precise positioning of the QSLS and payload swing angle regulation. In [15], a unified control framework based on energy function analysis was constructed to address the challenges of quadrotor positioning, payload swing elimination and vertical motion control simultaneously. In [16], the energy function was combined with the adaptive control method to handle the issue of variable tether length in QSLS. In [17], backstepping control was optimized via an improved grey wolf algorithm to enhance load tracking for complex trajectories. However, the detrimental influence of external disturbances on the suspension system has often been overlooked in much of the existing literature. Therefore, there is an urgent need to develop an efficient disturbance rejection scheme to ensure that the QSLS can operate safely and stably in complex environments. In particular, the main practical motivation is to design an anti-swing controller that can maintain tracking and swing suppression performance even when unknown disturbance acts on the coupled system. This requires an explicit disturbance reconstruction and compensation mechanism rather than treating disturbance as secondary effects.

In practice, the unknown disturbances including wind gust and air turbulence can directly affect the trajectory tracking accuracy and pose a significant threat to the flight performance of the QSLS. To address this difficulty, the active disturbance rejection control (ADRC) strategy pioneered by Han [18] provides an effective disturbance compensation framework. As a core technology of ADRC, extended state observer (ESO) has demonstrated its potential both theoretically and practically. In [19], a modified nonlinear ESO was designed for handling the affine nonlinearities in dynamic systems. In [20], the sliding mode control technology was combined with the ESO approach to tackle the unknown disturbances in underwater robot operations. In [21], a novel ESO with time-varying gains was introduced, effectively solving the robust safe control of unmanned helicopter subjected to external disturbances. In [22], an ESO-based robust control strategy was designed for each subsystem of a fixed-wing UAV to improve its tracking performance and disturbance suppression ability. In [23], by adopting the ADRC technique, a feedback linearization controller was developed to cope with the model uncertainties and external disturbances in UAV systems. In [24], a cascaded ADRC framework was established for trajectory tracking control of quadrotor UAVs under external disturbances and model uncertainties. In [25], a hierarchical control framework with a disturbance estimator was proposed to handle limited inner-loop bandwidth. This approach ensures robust outer-loop tracking and anti-swing performance despite hardware constraints. However, it is important to note that the estimation accuracy and convergence speed of the ESO are strongly influenced by its design parameters, which largely determine the overall performance of the system. In particular, the high gain tuning parameters often lead to the so-called “peaking phenomenon” in the initial stage. More critically, the traditional ESO design process does not make full use of all the system information, which also affects the final estimation accuracy. Therefore, exploring novel anti-disturbance strategy is undoubtedly a necessary and challenging task.

In addition, finite-time control can improve the rapidity of the system, which is exactly what the suspension system urgently needs, and many valuable research achievements have been made in recent years [26–30]. In [26], a neural-based global finite-time convergence fault tolerance control strategy was proposed to identify and compensate for actuator faults of the unmanned helicopter. In [27], a finite-time stabilized adaptive fuzzy control approach was developed for nonlinear strict-feedback systems to enhance tracking performance. In [28], an adaptive finite-time tracking controller was designed for nonlinear systems under actuator faults. The application problem of finite-time attitude tracking control for unmanned helicopter was investigated in [29]. In [30], the adaptive sliding-mode observer was constructed to achieve the finite-time convergence of aerial manipulators, effectively compensating for both exogenous and endogenous uncertainties. Recently, various advanced finite-time control schemes have been further explored to enhance the tracking precision and robustness of different nonlinear systems [31–36]. In [31,32], terminal sliding mode control strategies were developed for servo motor systems to achieve fast finite-time stable sliding motion independent of initial conditions. To further address lumped disturbances and uncertainties, composite finite-time control frameworks incorporating terminal sliding mode and observers were successfully applied to underactuated flexible joint robots [33], vehicle steer-by-wire systems [34], and air conditioning systems [35]. More recently, in [36], an extreme learning machine-based integral terminal sliding mode approach was introduced for permanent magnet synchronous motors to achieve superior speed regulation and uncertainty compensation. However, when the problems of anti-swing and disturbance rejection are coupled simultaneously, the finite-time flight control for QSLS warrants further exploration.

Inspired by the above-mentioned research, this paper proposes a novel finite-time anti-swing control framework for the QSLS in the presence of external disturbances. The main contributions of this work are summarized as follows:

1) Compared with traditional anti-swing methods [37], the energy function-based approach not only simplifies the controller design procedure but also enhances the global convergence performance of the QSLS by effectively incorporating swing angle information.
2) Compared with traditional ESO methods [38], the designed compensation function observer achieves a faster convergence rate and higher observation accuracy with smaller observer gains, thereby enhancing the robustness of the QSLS system;
3) The designed finite-time anti-swing control strategy ensures bounded convergence for the QSLS while optimizing the transient performance of the entire closed-loop system.

The remaining part of this paper is organized as follows. Section 2 derives the nonlinear dynamic model and theoretical foundations. Section 3 develops the finite-time anti-swing control framework, followed by comparative simulation analyses in Section 4. Finally, conclusions are summarized in Section 5.

## 2 Problem statement

**Fig 1:**
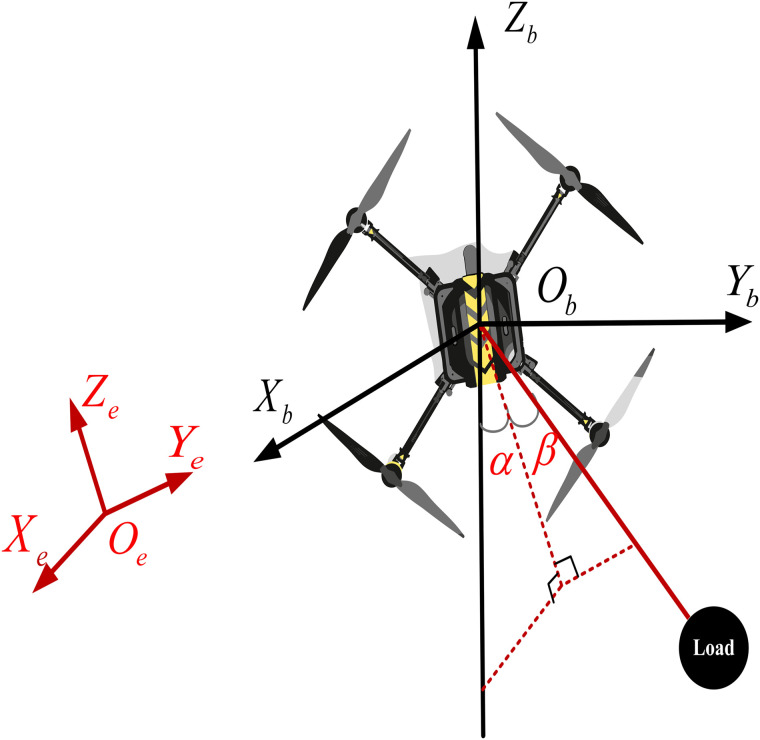
The structure of the QSLS.

The QSLS comprises a quadrotor UAV, a rigid string and a payload. In order to facilitate the understanding, the physical structure of the QSLS is shown in Fig 1, where $E_{e}=\left\{X_{e},Y_{e},Z_{e},O_{e}\right\}$ denotes the earth-fixed frame, $B_{b}=\left\{X_{b},Y_{b},Z_{b},O_{b}\right\}$ denotes the body-fixed frame, *O*_*e*_ is located on the ground, *O*_*b*_ coincides with the centroid of the quadrotor, the angles α and β represent the swing angles of the payload, respectively.

The position loop nonlinear dynamics of the QSLS are formulated through the Newton-Euler principles as follows [11,14]:


$$\left\{ \begin{array}{c} m_{Q}{\dot{V}}_{Q}=-G_{Q}+U_{1}+h_{0}+d\\ m_{L}{\dot{V}}_{L}=-G_{L}-h_{0} \end{array}\right.$$ (1)

where $G_{Q}=m_{Q}g\rho_{s}$, $G_{L}=m_{L}g\rho_{s}$, $\rho_{s}=[0,0,1{]}^{T}$, *m*_*Q*_, *m*_*L*_ and *g* denote the mass of quadrotor, the mass of slung-load, and the gravitational acceleration, respectively. *V*_*Q*_ = [*V*_*x*_, *V*_*y*_, *V*_*z*_]^*T*^ is the velocity vector of the quadrotor, *V*_*L*_ = [*v*_*x*_, *v*_*y*_, *v*_*z*_]^*T*^ is the velocity vector of the slung-load. *U*_*1*_ = [*u*_*x*_, *u*_*y*_, *u*_*z*_]^*T*^ is the control input vector, $h_{0}=[h_{x},h_{y},h_{z}{]}^{T}=[h\sin\alpha \cos\beta ,h\sin\beta ,-h\cos\alpha \cos\beta {]}^{T}$ is the cable tension vector in the body-fixed frame *B*_*b*_, *h* is the tension of the string, and *d* = [*d*_*x*_, *d*_*y*_, *d*_*z*_]^*T*^ represents the external disturbance vector.

From Fig 1, the spatial relationship between the quadrotor and slung-load can be mathematically expressed as


$$\xi_{L}=\xi_{Q}+p_{L}\Theta_{a}$$ (2)

where $\xi_{Q}=[x_{Q},y_{Q},z_{Q}{]}^{T}$ and $\xi_{L}=[x_{L},y_{L},z_{L}{]}^{T}$ denote the quadrotor and slung-load position vectors, respectively. *p*_*L*_ denotes the cable length, and $\Theta_{a}=[\Theta_{a1},\Theta_{a2},\Theta_{a3}{]}^{T}=[\sin\alpha \cos\beta ,\sin\beta ,-\cos\alpha \cos\beta {]}^{T}$.

Differentiating equation (2) twice with respect to time, the relative acceleration dynamics between the quadrotor and its slung-load can be derived as


$${\dot{V}}_{L}={\dot{V}}_{Q}+p_{L}\Theta_{b}$$ (3)

where $\Theta_{b}={\ddot{\Theta }}_{a}={\left[\Theta_{b1},\Theta_{b2},\Theta_{b3}\right]}^{T}$ with


$$\begin{array}{c} \left\{ \begin{array}{c} \Theta_{b1}=\ddot{\alpha }\cos\alpha \cos\beta -\ddot{\beta }\sin\alpha \sin\beta -{\dot{\alpha }}^{2}\sin\alpha \cos\beta \\ \;-\;{\dot{\beta }}^{2}\sin\alpha \cos\beta -2\dot{\alpha }\dot{\beta }\cos\alpha \sin\beta \\ \Theta_{b2}=\ddot{\beta }\cos\beta -{\dot{\beta }}^{2}\sin\beta \\ \Theta_{b3}=\ddot{\alpha }\sin\alpha \cos\beta +\ddot{\beta }\cos\alpha \sin\beta +{\dot{\alpha }}^{2}\cos\alpha \cos\beta \\ \;+\;{\dot{\beta }}^{2}\cos\alpha \cos\beta -2\dot{\alpha }\dot{\beta }\sin\alpha \sin\beta \end{array}\right. \end{array}$$ (4)

According to equations (1) to (4), the kinematic equation of the QSLS is given by


$${\dot{V}}_{Q}=\frac{U_{1}-m_{L}p_{L}\Theta_{b}+d}{m_{Q}+m_{L}}-g\rho_{s}$$ (5)

Based on equations (1), (4), and (5), the swing angle constraint equations are established as follows:


$$\left\{ \begin{array}{c} ((m_{Q}+m_{L}){\ddot{x}}_{Q}-(u_{x}+d_{x}))\cos\alpha \cos\beta +((m_{Q}+m_{L}){\ddot{z}}_{Q}\\ -(u_{z}+d_{z}))\sin\alpha \cos\beta +m_{L}p_{L}(\ddot{\alpha }\cos^{2}\beta -2\dot{\alpha }\dot{\beta }\sin\beta \cos\beta )=0\\ -((m_{Q}+m_{L}){\ddot{x}}_{Q}-(u_{x}+d_{x}))\sin\alpha \sin\beta +((m_{Q}+m_{L}){\ddot{y}}_{Q}-(u_{y}+d_{y}))\cos\beta \\ +((m_{Q}+m_{L}){\ddot{z}}_{Q}-(u_{z}+d_{z}))\cos\alpha \sin\beta +m_{L}p_{L}(\ddot{\beta }+{\dot{\alpha }}^{2}\sin\beta \cos\beta )=0 \end{array}\right.$$ (6)

Furthermore, by integrating equations (1) to (6), we have


$$R(n)\ddot{n}+J(n,\dot{n})\dot{n}+M(n)=H+d^{\prime }$$ (7)

where $H=[U_{1}-(m_{Q}+m_{L})g\rho_{s},0,0{]}^{T}$ represents the resultant external force acting on the system, $n=[x_{Q},y_{Q},z_{Q},\alpha ,\beta {]}^{T}$ denotes the state vector of QSLS, $d^{\prime }=[d,0,0{]}^{T}$. $R(n)\in R^{5\times 5}$, $J(n,\dot{n})\in R^{5\times 5}$, and $M(n)\in R^{5}$ are defined as the system’s inertia matrix, Coriolis coupling matrix, and gravity effect vector. Their mathematical formulations can be written as


$$\begin{array}{c} \;R(n)=\left[\begin{array}{ccccc} {}*20cR_{11} & 0 & 0 & R_{14} & R_{15}\\ 0 & R_{22} & 0 & 0 & R_{25}\\ 0 & 0 & R_{33} & R_{34} & R_{35}\\ R_{41} & 0 & R_{43} & R_{44} & 0\\ R_{51} & R_{52} & R_{53} & 0 & R_{55} \end{array}\right] \end{array}$$ (8)

$$\begin{array}{c} J(n,\dot{n})=\left[\begin{array}{ccccc} {}*20c0 & 0 & 0 & J_{14} & J_{15}\\ 0 & 0 & 0 & 0 & J_{25}\\ 0 & 0 & 0 & J_{34} & J_{35}\\ 0 & 0 & 0 & J_{44} & J_{45}\\ 0 & 0 & 0 & J_{54} & 0 \end{array}\right] \end{array}$$ (9)

$$\begin{array}{c} M(n)=[\begin{array}{ccccc} {}*20c0 & 0 & 0 & M_{14} & M_{15} \end{array}{]}^{T} \end{array}$$ (10)

where $R_{11}=R_{22}=R_{33}=m_{Q}+m_{L}$, $R_{44}=m_{L}p_{L}^{2}c_{\beta }^{2}$, $R_{55}=m_{L}p_{L}^{2}$, $R_{14}=R_{41}\;=m_{L}p_{L}c_{\alpha }c_{\beta }$, $R_{15}=R_{51}=-m_{L}p_{L}s_{\alpha }s_{\beta }$, $R_{34}=R_{43}=m_{L}p_{L}s_{\alpha }c_{\beta }$, $R_{25}=R_{52}=m_{L}p_{L}c_{\beta }$, $R_{35}=R_{53}=m_{L}p_{L}c_{\alpha }s_{\beta }$, $J_{14}=-m_{L}p_{L}(s_{\alpha }c_{\beta }\dot{\alpha }+c_{\alpha }s_{\beta }\dot{\beta })$, $J_{15}=-m_{L}p_{L}(s_{\alpha }c_{\beta }\dot{\beta }+c_{\alpha }s_{\beta }\dot{\alpha })$, $J_{25}=-m_{L}p_{L}s_{\beta }\dot{\beta }$, $J_{34}=m_{L}p_{L}(c_{\alpha }c_{\beta }\dot{\alpha }-s_{\alpha }s_{\beta }\dot{\beta })$, $J_{35}=m_{L}p_{L}(c_{\alpha }c_{\beta }\dot{\beta }-s_{\alpha }s_{\beta }\dot{\alpha })$, $J_{44}=-m_{L}p_{L}^{2}s_{\beta }c_{\beta }\dot{\beta }$, $J_{45}=-m_{L}p_{L}^{2}s_{\beta }c_{\beta }\dot{\alpha }$, $J_{54}=m_{L}p_{L}^{2}s_{\beta }c_{\beta }\dot{\alpha }$, $M_{14}=m_{L}p_{L}gs_{\alpha }c_{\beta }$, $M_{15}=m_{L}p_{L}gc_{\alpha }s_{\beta }$. The abbreviations c_*_ and s_*_ represent cos(*) and sin(*) in this work, respectively.

The control objective of this work is to design an energy function-based finite-time controller capable of tracking the desired trajectory accurately despite the presence of unknown disturbances and restraining the swing motion of the slung-load efficiently. To attain this control objective, a series of essential assumptions and properties are introduced and utilized in the subsequent stages of development.

**Assumption 1** [14,15]. For dynamic analysis, the suspension mechanism is supposed as massless and inelastic.

**Assumption 2** [6,14]. The payload maintains a downward position relative to the quadrotor, and the swing angles are constrained by $\left|\alpha \right|<\frac{\pi }{2},\left|\beta \right|<\frac{\pi }{2}$.

**Assumption 3** [30,39]. There exist two positive scalars $d_{\rho }$ and $d_{\sigma }$ such that


$$\left\|d\right\|\leq d_{\rho },\left\|\dot{d}\right\|\leq d_{\sigma }$$ (11)

**Property 1** [6,40,41]. The inertia matrix *R*(*n*) possesses invertibility and positive definiteness, with a positive bound *R*_*q*_ satisfying $\left\|R^{-1}(n)\right\|\leq R_{q}$.

**Remark 1**. The assumptions made above are essential for the theoretical formulation and practical stability of the QSLS. In practice, the mass of the suspension mechanism is typically much smaller than that of the QSLS. Hence, the Assumption 1 is reasonable and can be found in [14,15]. Assumption 2 ensures that the payload remains within a safe operating hemisphere. This configuration is intentionally maintained to prevent collisions with the quadrotor and ensures that the trigonometric terms in the dynamic model remain well-defined, which is logically reasonable and widely adopted in literature [6,14,15,40,41]. Furthermore, Assumption 3 reflects the physical reality that external disturbance (e.g., wind gusts) possess finite energy and change rates. This boundedness is a prerequisite for any disturbance observer, including the proposed compensation function observer, to achieve reliable reconstruction and compensation [30,39]. To sum up, the assumptions made in this paper are reasonable and can facilitate the advancement of controller design.

## 3 Main results

**Fig 2:**
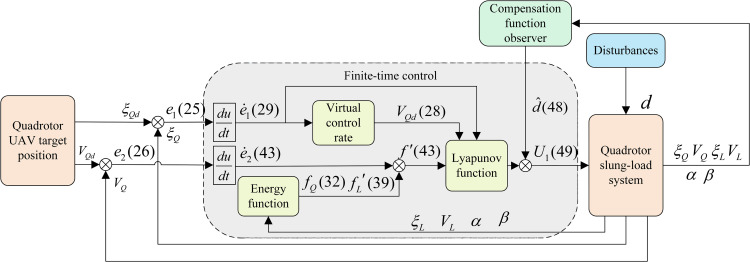
Architecture of the proposed control scheme.

During the actual flight of the QSLS, external disturbances pose a substantial threat to flight safety. Therefore, this section introduces a compensation function observer to precisely compensate for these disturbances. Furthermore, an anti-swing control strategy founded on the energy function approach is proposed to deal with the intricate coupling dynamics between the quadrotor and its slung-load. Ultimately, the finite-time approach is employed to improve the convergence speed of the QSLS. The structure of the overall control scheme is illustrated in Fig 2.

### 3.1 Design of compensation function observer

By reformulating equation (7), this model is adapted to support further analysis. Designing state variables *z*_1_ = *n* and $z_{2}=\dot{n}$, it gives


$$\left\{ \begin{array}{c} {\dot{z}}_{1}=z_{2}\\ R(z_{1}){\dot{z}}_{2}+J(z_{1},z_{2})z_{2}+M(z_{1})=H+d^{\prime } \end{array}\right.$$ (12)

According to Property 1, equations (12) can be further represented as


$$\left\{ \begin{array}{c} {\dot{z}}_{1}=z_{2}\\ {\dot{z}}_{2}=R^{-1}(z_{1})(-J(z_{1},z_{2})z_{2}-M(z_{1})+H)+D \end{array}\right.$$ (13)

where $D=R^{-1}(z_{1})d^{\prime }$.

In accordance with Assumption 3, it is known that *D* is bounded. Then, considering *D* as a new state *z*_3_ with $\dot{D}=\varepsilon_{d}$, we have


$$\left\{ \begin{array}{c} {\dot{z}}_{1}=z_{2}\\ {\dot{z}}_{2}=R^{-1}(z_{1})(-J(z_{1},z_{2})z_{2}-M(z_{1})+H)+z_{3}\\ {\dot{z}}_{3}=\varepsilon_{d} \end{array}\right.$$ (14)

According to equations (14), the compensation function observer for the QSLS is proposed as [42,43]


$$\left\{ \begin{array}{c} {\dot{\hat{z}}}_{1}={\hat{z}}_{2}\\ {\dot{\hat{z}}}_{2}=R^{-1}(z_{1})(-J(z_{1},z_{2})z_{2}-M(z_{1})+H)+z_{0}+N_{1}\lambda_{1}+N_{2}\lambda_{2}\\ {\dot{z}}_{0}=\eta_{1}N_{1}\lambda_{1}+\eta_{2}N_{2}\lambda_{2}\\ {\hat{z}}_{3}=z_{0}+N_{1}\lambda_{1}+N_{2}\lambda_{2} \end{array}\right.$$ (15)

where ${\hat{z}}_{1}$, ${\hat{z}}_{2}$ and ${\hat{z}}_{3}$ are the estimates of *z*_1_, *z*_2_ and *z*_3_, respectively. *z*_0_ is the intermediate variable, $\eta_{1}>0$ and $\eta_{2}>0$ are the positive constants, $N_{1}\in R^{5\times 5}$ and $N_{2}\in R^{5\times 5}$ are the designed positive definite matrixes. $\lambda_{1}=z_{1}-{\hat{z}}_{1}$, $\lambda_{2}=z_{2}-{\hat{z}}_{2}$ and $\lambda_{3}=z_{3}-{\hat{z}}_{3}$ are the estimation errors of *z*_1_, *z*_2_ and *z*_3_, respectively. Following [42,43], this structure leverages the intermediate variable *z*_0_ to utilize both position and velocity observation errors, which effectively suppresses the “initial peaking” phenomenon and ensures high estimation accuracy.

Differentiating $\lambda_{i}(i=1,2,3)$ with respect to time gives


$${\dot{\lambda }}_{1}={\dot{z}}_{1}-{\dot{\hat{z}}}_{1}=z_{2}-{\hat{z}}_{2}=\lambda_{2}$$ (16)

$${\dot{\lambda }}_{2}={\dot{z}}_{2}-{\dot{\hat{z}}}_{2}=z_{3}-{\hat{z}}_{3}=\lambda_{3}$$ (17)

$$\begin{array}{cc} {\dot{\lambda }}_{3} & =\varepsilon_{d}-\eta_{1}N_{1}\lambda_{1}-\eta_{2}N_{2}\lambda_{2}-N_{1}{\dot{\lambda }}_{1}-N_{2}{\dot{\lambda }}_{2}\\ & =\varepsilon_{d}-\eta_{1}N_{1}\lambda_{1}-\eta_{2}N_{2}\lambda_{2}-N_{1}\lambda_{2}-N_{2}\lambda_{3}\\ & =-\eta_{1}N_{1}\lambda_{1}-(\eta_{2}N_{2}+N_{1})\lambda_{2}-N_{2}\lambda_{3}+\varepsilon_{d} \end{array}$$ (18)

By defining $\Upsilon =[\lambda_{1}^{T},\lambda_{2}^{T},\lambda_{3}^{T}{]}^{T}$, the following relationship is obtained


$$\begin{array}{cc} \dot{\Upsilon } & =\left[\begin{array}{ccc} 0c0_{5\times 5} & I_{5\times 5} & 0_{5\times 5}\\ 0_{5\times 5} & 0_{5\times 5} & I_{5\times 5}\\ -\eta_{1}N_{1} & -(\eta_{2}N_{2}+N_{1}) & -N_{2} \end{array}\right]\Upsilon +\left[\begin{array}{c} 0c0_{5\times 1}\\ 0_{5\times 1}\\ \varepsilon_{d} \end{array}\right]\\ & =\varpi_{\lambda }\Upsilon +\varepsilon_{e} \end{array}$$ (19)

where the subscripts 5 × 5 and 5 × 1 specify the dimensional information of matrix. 0_5×5_ is the zero matrix and *I*_5×5_ is the identity matrix. Moreover, the bounded $\varepsilon_{d}$ implies $\varepsilon_{e}$ is also bounded.

Here, by selecting parameters properly, $\varpi_{\lambda }$ can be guaranteed to be a Hurwitz matrix. This implies the existence of a positive definite matrix $Q_{\lambda }$ verifying


$$\varpi_{\lambda }^{T}Q_{\lambda }+Q_{\lambda }\varpi_{\lambda }=-W_{\lambda }$$ (20)

where $W_{\lambda }$ is the positive definite matrix.

The proposed Lyapunov function candidate is given by


$$V_{1}(t)=\Upsilon^{T}Q_{\lambda }\Upsilon$$ (21)

By invoking equations (19) to (20), we can obtain


$$\begin{array}{cc} {\dot{V}}_{1}(t) & =\Upsilon^{T}Q_{\lambda }(\varpi_{\lambda }\Upsilon +\varepsilon_{\lambda })+(\varpi_{\lambda }\Upsilon +\varepsilon_{e}{)}^{T}Q_{\lambda }\Upsilon \\ & =\Upsilon^{T}(Q_{\lambda }\varpi_{\lambda }+\varpi_{\lambda }^{T}Q_{\lambda })\Upsilon +2\Upsilon^{T}Q_{\lambda }\varepsilon_{e}\\ & \leq -\Upsilon^{T}(W_{\lambda }-I_{15\times 15})\Upsilon +{\left\|Q_{\lambda }\varepsilon_{e}\right\|}^{2}\\ & \leq -\tau_{1}\Upsilon^{T}\Upsilon +\varepsilon_{dm} \end{array}$$ (22)

where $\tau_{1}=\lambda_{\min}(W_{\lambda }-I_{15\times 15})$ and $\varepsilon_{dm}={\left\|Q_{\lambda }\varepsilon_{e}\right\|}^{2}$.

### 3.2 Design of finite-time anti-swing control

To streamline the controller design process, the following lemmas are presented

**Lemma 1** [29,30]. Consider the positional loop equations for QSLS as equations (5) and (6). If there is a smooth positive definite function *V*(*x*) such that $\dot{V}(x)\leq -\delta_{1}V^{r}(x)+\delta_{2}$ always holds, where $\delta_{1}>0$, 0 < *r* < 1 and $0<\delta_{2}<\infty$ are real numbers, then the position of QSLS is finite-time stable. Meanwhile, the settling time *T*_*st*_ can be computed by $T_{st}\leq \frac{1}{(1-r)\vartheta \delta_{1}}[V^{1-r}(x(0))-(\frac{\delta_{2}}{(1-\vartheta )\delta_{1}}{)}^{\frac{1-r}{r}}]$ with 0<ϑ<1 being a constant.

**Lemma 2** [26,40]. For any real numbers $\varepsilon_{i}(i=1,2,\ldots ,n)$, the following relationship holds


$$\left\{ \begin{array}{c} (\sum_{i=1}^{n}\left|\varepsilon_{i}\right|{)}^{\nu }\leq \sum_{i=1}^{n}{\left|\varepsilon_{i}\right|}^{\nu }\\ (\sum_{i=1}^{n}{\left|\varepsilon_{i}\right|}^{2}{)}^{\rho }\leq (\sum_{i=1}^{n}{\left|\varepsilon_{i}\right|}^{\rho }{)}^{2} \end{array}\right.$$ (23)

where 0<ν<1 and 0<ρ<2 are constants.

**Lemma 3** [26]. For any set of positive constants *b*_1_, *b*_2_ and *b*_3_, the following inequation holds


$${\left|\sigma_{1}\right|}^{b_{1}}{\left|\sigma_{2}\right|}^{b_{2}}\leq \frac{b_{1}}{b_{1}+b_{2}}b_{3}{\left|\sigma_{1}\right|}^{b_{1}+b_{2}}+\frac{b_{2}}{b_{1}+b_{2}}b_{3}^{\frac{b_{1}}{b_{2}}}{\left|\sigma_{2}\right|}^{b_{1}+b_{2}}$$ (24)

where $\sigma_{1}$ and $\sigma_{2}$ are real variables.

Based on equation (5), the following tracking error vectors are defined:


$$e_{1}=\xi_{Q}-\xi_{Q_{d}}$$ (25)

$$e_{2}=V_{Q}-V_{Q_{d}}$$ (26)

Taking the derivative of *e*_1_ yields


$${\dot{e}}_{1}={\dot{\xi }}_{Q}-{\dot{\xi }}_{Q_{d}}=e_{2}+V_{Q_{d}}-{\dot{\xi }}_{Q_{d}}$$ (27)

The virtual control law is designed as


$$V_{Q_{d}}={\dot{\xi }}_{Q_{d}}-e_{1}^{2r-1}$$ (28)

where $e_{1}^{2r-1}={\left[e_{11}^{2r-1},e_{12}^{2r-1},e_{13}^{2r-1}\right]}^{T}$, r∈(0,1) is a designed constant.

Substituting equation (28) into equation (27) yields


$${\dot{e}}_{1}=e_{2}-e_{1}^{2r-1}$$ (29)

The Lyapunov function candidate is selected as


$$V_{2}(t)=\frac{1}{2}e_{1}^{T}S_{1}e_{1}$$ (30)

where $S_{1}=\text{diag}\left\{S_{11},S_{12},S_{13}\right\}$ is the designed positive definite diagonal matrix.

Taking the derivative of *V*_2_(*t*) and invoking equation (29) yields


$${\dot{V}}_{2}(t)=e_{1}^{T}S_{1}{\dot{e}}_{1}=e_{1}^{T}S_{1}e_{2}-e_{1}^{T}S_{1}e_{1}^{2r-1}$$ (31)

To simultaneously achieve trajectory tracking and anti-swing control of the QSLS, a finite-time swing rejection control scheme is derived from the energy function of the QSLS. First, the kinetic energy of the quadrotor is defined as


$$f_{Q}=\frac{1}{2}m_{Q}({\dot{x}}_{Q}^{2}+{\dot{y}}_{Q}^{2}+{\dot{z}}_{Q}^{2})$$ (32)

Differentiating equation (32) yields


$${\dot{f}}_{Q}={\dot{x}}_{Q}\left(m_{Q}{\ddot{x}}_{Q}\right)+{\dot{y}}_{Q}\left(m_{Q}{\ddot{y}}_{Q}\right)+{\dot{z}}_{Q}\left(m_{Q}{\ddot{z}}_{Q}\right)$$ (33)

Subsequently, the kinetic energy of slung-load is expressed as


$$f_{L}=\frac{1}{2}m_{L}({\dot{x}}_{L}^{2}+{\dot{y}}_{L}^{2}+{\dot{z}}_{L}^{2})$$ (34)

Taking the derivative of *f*_*L*_ gives


$${\dot{f}}_{L}={\dot{x}}_{L}\left(m_{L}{\ddot{x}}_{L}\right)+{\dot{y}}_{L}\left(m_{L}{\ddot{y}}_{L}\right)+{\dot{z}}_{L}\left(m_{L}{\ddot{z}}_{L}\right)$$ (35)

Differentiating equation (2) yields


$${\dot{\xi }}_{L}={\dot{\xi }}_{Q}+\Lambda$$ (36)

where


$$\Lambda =\left[\begin{array}{c} \Lambda_{x}\\ \Lambda_{y}\\ \Lambda_{z} \end{array}\right]=p_{L}{\dot{\Theta }}_{a}=\left[\begin{array}{c} p_{L}(\cos\alpha \cos\beta \dot{\alpha }-\sin\alpha \sin\beta \dot{\beta })\\ p_{L}\cos\beta \dot{\beta }\\ -p_{L}(\sin\alpha \cos\beta \dot{\alpha }+\cos\alpha \sin\beta \dot{\beta }) \end{array}\right].$$

Substituting equations (1) and (36) into equation (35), it gives


$$\begin{array}{cc} {\dot{f}}_{L} & ={\dot{x}}_{Q}\left(-h\sin\alpha \cos\beta \right)+{\dot{y}}_{Q}\left(-h\sin\beta \right)+{\dot{z}}_{Q}\left(-h\cos\alpha \cos\beta \right)\\ & \;-m_{L}gp_{L}\left(\sin\alpha \cos\beta \dot{\alpha }+\cos\alpha \sin\beta \dot{\beta }\right) \end{array}$$ (37)

Then, the position of the slung-load is defined as the zero potential energy plane when the swing angle is set to zero. Consequently, the potential energy equation of the slung-load can be expressed as follows:


$$f_{Lp}=p_{L}m_{L}g(1-\cos\alpha \cos\beta )$$ (38)

The total energy function $f_{L}^{\prime }$ containing equations (34) and (38) is constructed as


$$\begin{array}{cc} f_{L}^{\prime } & =f_{L}+f_{Lp}\\ & =\frac{1}{2}m_{L}({\dot{x}}_{L}^{2}+{\dot{y}}_{L}^{2}+{\dot{z}}_{L}^{2})+p_{L}m_{L}g(1-\cos\alpha \cos\beta ) \end{array}$$ (39)

By differentiating equation (39), it can be obtained that


$${{\dot{f}}^{\prime }}_{L}={\dot{x}}_{Q}\left(-h\sin\alpha \cos\beta \right)+{\dot{y}}_{Q}\left(-hsin\beta \right)+{\dot{z}}_{Q}\left(-h\cos\alpha \cos\beta \right)$$ (40)

Invoking equations (32) and (39), the complete energy function is constructed as


$$\begin{array}{cc} f & =S_{Q}f_{Q}+S_{L}f_{L}^{\prime }\\ & =\frac{1}{2}S_{Q}m_{Q}({\dot{x}}_{Q}^{2}+{\dot{y}}_{Q}^{2}+{\dot{z}}_{Q}^{2})+\frac{1}{2}S_{L}m_{L}({\dot{x}}_{L}^{2}+{\dot{y}}_{L}^{2}+{\dot{z}}_{L}^{2})\\ & \;+S_{L}p_{L}m_{L}g(1-\cos\alpha \cos\beta ) \end{array}$$ (41)

where *S*_*Q*_ > 0 and *S*_*L*_ > 0 are energy coefficients.

Finally, in order to combine the energy function with the velocity error of the QSLS, an auxiliary energy function is defined as


$$\begin{array}{cc} f_{d} & =\frac{1}{2}S_{Q}m_{Q}\left(V_{xd}^{2}+V_{yd}^{2}+{\dot{V}}_{zd}^{2}\right)+\frac{1}{2}S_{L}m_{L}\left(v_{xd}^{2}+v_{yd}^{2}+v_{zd}^{2}\right)\\ & \;-S_{Q}m_{Q}\left(V_{x}V_{xd}+V_{y}V_{yd}+V_{z}V_{zd}\right)-S_{L}m_{L}\left(v_{x}v_{xd}+v_{y}v_{yd}+v_{z}v_{zd}\right) \end{array}$$ (42)

Combining it with equation (41) to form a new energy function as follows:


$$\begin{array}{cc} f^{\prime } & =f+f_{d}=\frac{1}{2}S_{Q}m_{Q}({\dot{x}}_{Q}^{2}+{\dot{y}}_{Q}^{2}+{\dot{z}}_{Q}^{2})+\frac{1}{2}S_{Q}m_{Q}(V_{xd}^{2}+V_{yd}^{2}+{\dot{V}}_{zd}^{2})\\ & \;-S_{Q}m_{Q}(V_{x}V_{xd}+V_{y}V_{yd}+V_{z}V_{zd})+\frac{1}{2}S_{L}m_{L}({\dot{x}}_{L}^{2}+{\dot{y}}_{L}^{2}+{\dot{z}}_{L}^{2})\\ & \;+\frac{1}{2}S_{L}m_{L}(v_{xd}^{2}+v_{yd}^{2}+v_{zd}^{2})-S_{L}m_{L}(v_{x}v_{xd}+v_{y}v_{yd}+v_{z}v_{zd})\\ & \;+S_{L}p_{L}m_{L}g(1-\cos\alpha \cos\beta )\\ & =\frac{1}{2}S_{Q}m_{Q}(V_{x}^{2}+V_{y}^{2}+V_{z}^{2})+\frac{1}{2}S_{Q}m_{Q}(V_{xd}^{2}+V_{yd}^{2}+{\dot{V}}_{zd}^{2})\\ & \;-S_{Q}m_{Q}(V_{x}V_{xd}+V_{y}V_{yd}+V_{z}V_{zd})+\frac{1}{2}S_{L}m_{L}(v_{x}^{2}+v_{y}^{2}+v_{z}^{2})\\ & \;+\frac{1}{2}S_{L}m_{L}(v_{xd}^{2}+v_{yd}^{2}+v_{zd}^{2})-S_{L}m_{L}(v_{x}v_{xd}+v_{y}v_{yd}+v_{z}v_{zd})\\ & \;+S_{L}p_{L}m_{L}g(1-\cos\alpha \cos\beta )\\ & =\frac{1}{2}S_{Q}m_{Q}e_{2}^{T}e_{2}+\frac{1}{2}S_{L}m_{L}L^{T}L+S_{L}p_{L}m_{L}g(1-\cos\alpha \cos\beta ) \end{array}$$ (43)

where *L* = *V*_*L*_ − *V*_*Ld*_.

By Assumption 2, the inequality (1−cosαcosβ)≥0 holds, which implies that $f^{\prime }$ is a positive definite function.

The relationship between the desired trajectories of the slung-load and the quadrotor is given by


$$V_{Qd}=V_{Ld}$$ (44)

Finally, since there is no term directly related to *L* in the QSLS model, it is necessary to transform the term. By invoking equations (36) and (44), equation (43) can be rewritten as follows:


$$\begin{array}{cc} f^{\prime } & =\frac{1}{2}\left(S_{Q}m_{Q}+S_{L}m_{L}\right)e_{2}^{T}e_{2}+S_{L}m_{L}p_{L}\Lambda^{T}e_{2}\\ & \;+S_{L}p_{L}m_{L}g(1-\cos\alpha \cos\beta )+\frac{1}{2}S_{L}m_{L}p_{L}^{2}\Lambda^{T}\Lambda \end{array}$$ (45)

From equations (1), (33), (36), (40), and (44), the derivative of $f^{\prime }$ is given by


$$\begin{array}{cc} {\dot{f}}^{\prime } & =S_{Q}e_{2}^{T}(m_{Q}{\dot{V}}_{Q}-m_{Q}{\dot{V}}_{Qd})+S_{L}L^{T}(m_{L}{\dot{V}}_{L}-m_{L}{\dot{V}}_{Ld})\\ & \;+S_{L}p_{L}m_{L}g(\sin\alpha \cos\beta \dot{\alpha }+\cos\alpha \sin\beta \dot{\beta })\\ & =S_{Q}e_{2}^{T}(m_{Q}{\dot{V}}_{Q}-m_{Q}{\dot{V}}_{Qd})+S_{L}(e_{2}+\Lambda {)}^{T}(-G_{L}-h_{0}-m_{L}{\dot{V}}_{d})\\ & \;+S_{L}p_{L}m_{L}g(\sin\alpha \cos\beta \dot{\alpha }+\cos\alpha \sin\beta \dot{\beta })\\ & =S_{Q}e_{2}^{T}(m_{Q}{\dot{V}}_{Q}-m_{Q}{\dot{V}}_{Qd})-S_{L}m_{L}\Lambda^{T}{\dot{V}}_{d}\\ & \;+S_{L}e_{2}^{T}(-G_{L}-G_{Q}+U_{1}+d-m_{Q}{\dot{V}}_{Q}-m_{L}{\dot{V}}_{Ld})\\ & =e_{2}^{T}((S_{Q}-S_{L})m_{Q}{\dot{V}}_{Q}-S_{L}(-G_{L}-G_{Q}+U_{1}+d-m_{L}{\dot{V}}_{Ld}))\\ & \;-S_{L}m_{L}\Lambda^{T}{\dot{V}}_{d} \end{array}$$ (46)

By substituting equation (5) into equation (46), the following result can be obtained


$$\begin{array}{cc} {\dot{f}}^{\prime } & =e_{2}^{T}(\frac{m_{Q}S_{Q}+m_{L}S_{L}}{m_{Q}+m_{L}}U_{1}-\frac{(S_{Q}-S_{L})m_{Q}m_{L}p_{L}}{m_{Q}+m_{L}}\Theta_{b}-(S_{Q}m_{Q}\\ & \;+S_{L}m_{L})g\rho_{s}+\frac{m_{Q}S_{Q}+m_{L}S_{L}}{m_{Q}+m_{L}}d-(m_{Q}S_{Q}+m_{L}S_{L}){\dot{V}}_{Qd})\\ & \;-S_{L}m_{L}\Lambda^{T}{\dot{V}}_{d} \end{array}$$ (47)

The estimate ${\hat{z}}_{3}$ of *z*_3_ is obtained from the compensation function observer designed in the previous section, which is related to $\hat{d}$ as


$$\hat{d}=I_{q}R(z_{1}){\hat{z}}_{3}$$ (48)

where $I_{q}=\left[\begin{array}{ccccc} 1 & 0 & 0 & 0 & 0\\ 0 & 1 & 0 & 0 & 0\\ 0 & 0 & 1 & 0 & 0 \end{array}\right]$.

Then, based on the QSLS position model (5) and the energy function (45), the finite-time anti-swing controller is designed as follows:


$$\begin{array}{cc} U_{1} & =k_{1}(-k_{2}S_{2}e_{2}^{2r-1}+k_{3}m_{Q}m_{L}u_{L}\Theta_{b}-k_{4}{\hat{d}}_{1}-\frac{1}{2}k_{4}^{2}e_{2}+k_{2}g\rho_{s}\\ & \;-S_{1}e_{1}+k_{2}{\dot{V}}_{Qd}-\frac{1}{2}\frac{e_{2}m_{L}^{2}S_{L}^{2}\Lambda^{T}\Lambda }{e_{2}^{T}e_{2}+\varepsilon_{0}}-\frac{1}{2}\frac{e_{2}k_{5}^{r}(\frac{1}{2}+\frac{\mu_{L}^{r}}{2^{r}})\Lambda^{T}\Lambda^{2r-1}}{e_{2}^{T}e_{2}+\varepsilon_{0}}) \end{array}$$ (49)

where $e_{2}^{2r-1}={\left[e_{21}^{2r-1},e_{22}^{2r-1},e_{23}^{2r-1}\right]}^{T}$, $k_{1}=\frac{m_{Q}+m_{L}}{S_{Q}m_{Q}+S_{L}m_{L}}$, $k_{2}=S_{Q}m_{Q}+S_{L}m_{L}$, $k_{3}=\frac{\left(S_{Q}-S_{L}\right)}{m_{Q}+m_{L}}$, $k_{4}=\frac{1}{k_{1}}$, *k*_5_ = *S*_*L*_*m*_*L*_*p*_*L*_, $S_{2}=\text{diag}\left\{S_{21},S_{22},S_{23}\right\}$ is the designed positive definite matrix, $\varepsilon_{0}$ is an extremely small positive constant.

Selecting the Lyapunov function candidate as


$$V_{3}(t)=V_{2}(t)+f^{\prime }$$ (50)

By substituting equation (45) into equation (50), the following result can be obtained


$$V_{3}(t)=\frac{1}{2}e_{1}^{T}S_{1}e_{1}+\frac{1}{2}k_{2}e_{2}^{T}e_{2}+k_{5}\Lambda^{T}e_{2}+\frac{1}{2}k_{5}p_{L}\Lambda^{T}\Lambda +C$$ (51)

where $C=k_{5}g(1-\cos\alpha \cos\beta ),$ *k*_5_ = *S*_*L*_*m*_*L*_*p*_*L*_.

Finally, by invoking equations (31), (47) and (49), the derivative of equation (51) can be reformulated as


$$\begin{array}{cc} {\dot{V}}_{3}(t) & ={\dot{V}}_{2}(t)+{\dot{f}}^{\prime }\\ & =e_{1}^{T}S_{1}(e_{2}-e_{1}^{2r-1})+e_{2}^{T}(\frac{m_{Q}S_{Q}+m_{L}S_{L}}{m_{Q}+m_{L}}U_{1}-\frac{(S_{Q}-S_{L})m_{Q}m_{L}p_{L}}{m_{Q}+m_{L}}\Theta_{b})\\ & \;-(S_{Q}m_{Q}+S_{L}m_{L})g\rho_{s}+\frac{m_{Q}S_{Q}+m_{L}S_{L}}{m_{Q}+m_{L}}d-(m_{Q}S_{Q}+m_{L}S_{L}){\dot{V}}_{Qd})\\ & \;-S_{L}m_{L}\Lambda^{T}{\dot{V}}_{Qd}\\ & \leq e_{1}^{T}S_{1}e_{2}-e_{1}^{T}S_{1}e_{1}^{2r-1}+\frac{1}{2}{\dot{V}}_{Qd}^{T}{\dot{V}}_{Qd}+e_{2}^{T}(-k_{2}S_{2}e_{2}^{2r-1}-S_{1}e_{1}\\ & \;+k_{4}{\tilde{d}}_{1}-\frac{1}{2}k_{4}^{2}e_{2}-\frac{1}{2}\frac{e_{2}m_{L}^{2}S_{L}^{2}\Lambda^{T}\Lambda }{e_{2}^{T}e_{2}+\varepsilon_{0}}-\frac{1}{2}\frac{e_{2}k_{5}^{r}(\frac{1}{2}+\frac{\mu_{L}^{r}}{2^{r}})\Lambda^{T}\Lambda^{2r-1}}{e_{2}^{T}e_{2}+\varepsilon_{0}})\\ & \leq -e_{1}^{T}S_{1}e_{1}^{2r-1}-k_{2}e_{2}^{T}S_{2}e_{2}^{2r-1}+\frac{1}{2}{\tilde{d}}_{1}^{T}{\tilde{d}}_{1}+\varepsilon_{Qd}\\ & \;-\frac{1}{2}\frac{e_{2}^{T}e_{2}m_{L}^{2}S_{L}^{2}\Lambda^{T}\Lambda }{e_{2}^{T}e_{2}+\varepsilon_{0}}-\frac{1}{2}\frac{e_{2}^{T}e_{2}k_{5}^{r}(\frac{1}{2}+\frac{\mu_{L}^{r}}{2^{r}})\Lambda^{T}\Lambda^{2r-1}}{e_{2}^{T}e_{2}+\varepsilon_{0}} \end{array}$$ (52)

where ${\tilde{d}}_{1}=(d_{1}-{\hat{d}}_{1})$, $\varepsilon_{Qd}=\frac{1}{2}{\dot{V}}_{Qd}^{T}{\dot{V}}_{Qd}$.

### 3.3 Stability analysis

To summarize the core contributions, the following theorem is presented:

**Theorem 1.** *Consider the disturbed QSLS nonlinear model (7). The compensation function observer is designed (15). By applying the robust finite-time anti-swing control strategy (49), the swing angles of the slung-load can be suppressed efficiently and all error signals are guaranteed to be bounded, enabling the system output to track the desired trajectory.*

**Proof.** Consider the Lyapunov function candidate as


$$\begin{array}{cc} V_{4}(t) & =V_{1}(t)+V_{3}(t)\\ & =\Upsilon^{T}Q_{\lambda }\Upsilon +\frac{1}{2}e_{1}^{T}S_{1}e_{1}+\frac{1}{2}k_{2}e_{2}^{T}e_{2}\\ & \;+k_{5}\Lambda^{T}e_{2}+\frac{1}{2}k_{5}p_{L}\Lambda^{T}\Lambda +C \end{array}$$ (53)

Invoking equations (22), (31) and (52), the time derivative of *V*_4_(*t*) is given by


$$\begin{array}{cc} {\dot{V}}_{4}(t) & ={\dot{V}}_{1}(t)+{\dot{V}}_{3}(t)\\ & \;\leq -\tau_{1}\Upsilon^{T}\Upsilon +\varepsilon_{dm}-\sum_{i=1}^{3}S_{1i}e_{1i}^{2r}-k_{2}\sum_{i=1}^{3}S_{2i}e_{2i}^{2r}+\varepsilon_{Qd}-\frac{1}{2}\frac{e_{2}^{T}e_{2}m_{L}^{2}S_{L}^{2}\Lambda^{T}\Lambda }{e_{2}^{T}e_{2}+\varepsilon_{0}}\\ & \;-\frac{1}{2}\frac{e_{2}^{T}e_{2}k_{5}^{r}(\frac{1}{2}+\frac{\mu_{L}^{r}}{2^{r}})\Lambda^{T}\Lambda^{2r-1}}{e_{2}^{T}e_{2}+\varepsilon_{0}}+\frac{1}{2}{{\tilde{d}}_{1}}^{T}{\tilde{d}}_{1}\;-k_{5}^{r}\Lambda^{T}e_{2}^{T}e_{2}^{r-1}\Lambda^{r-1}-C^{r}+C^{r}\\ & \;+k_{5}^{r}\Lambda^{T}e_{2}^{T}e_{2}^{r-1}\Lambda^{r-1}-\frac{1}{2^{r}}k_{5}^{r}p_{L}^{r}\Lambda^{T}\Lambda^{2r-1}+\frac{1}{2^{r}}k_{5}^{r}p_{L}^{r}\Lambda^{T}\Lambda^{2r-1}\\ & \leq -\tau_{1}\Upsilon^{T}\Upsilon +\varepsilon_{dm}-\sum_{i=1}^{3}S_{1i}e_{1i}^{2r}-k_{2}\sum_{i=1}^{3}S_{2i}e_{2i}^{2r}-\frac{1}{2}\frac{e_{2}^{T}e_{2}m_{L}^{2}S_{L}^{2}\Lambda^{T}\Lambda }{e_{2}^{T}e_{2}+\varepsilon_{0}}\\ & \;+\frac{1}{2}{{\tilde{d}}_{1}}^{T}{\tilde{d}}_{1}+\frac{1}{2}e_{2}^{T}e_{2}^{2r-1}\;-k_{5}^{r}\Lambda^{T}e_{2}^{T}e_{2}^{r-1}\Lambda^{r-1}+\varepsilon_{Qd}-\frac{1}{2^{r}}k_{5}^{r}p_{L}^{r}\Lambda^{T}\Lambda^{2r-1}\\ & \;-\frac{1}{2}\frac{e_{2}^{T}e_{2}k_{5}^{r}(\frac{1}{2}+\frac{\mu_{L}^{r}}{2^{r}})\Lambda^{T}\Lambda^{2r-1}}{e_{2}^{T}e_{2}+\varepsilon_{0}}-C^{r}+\frac{1}{2}k_{5}^{r}\Lambda^{T}\Lambda^{2r-1}+C^{r}\\ & \;\leq -\sum_{i=1}^{3}S_{1i}e_{1i}^{2r}-k_{2}\sum_{i=1}^{3}S_{3i}e_{2i}^{2r}-k_{5}^{r}\Lambda^{T}e_{2}^{T}e_{2}^{r-1}\Lambda^{r-1}\\ & \;-\frac{1}{2^{r}}k_{5}^{r}p_{L}^{r}\Lambda^{T}\Lambda^{2r-1}-C^{r}-\Pi_{1}+\Pi_{2} \end{array}$$ (54)

where $S_{3}=diag\left\{S_{31},S_{32},S_{32}\right\}=k_{2}S_{2}-\frac{1}{2}I_{3\times 3}$, $\Pi_{1}=(\tau_{1}-\tau_{2})\Upsilon^{T}\Upsilon$, $\Pi_{2}=\varepsilon_{dm}+C^{r}+\varepsilon_{Qd},\tau_{2}=\lambda_{\max}\left\{R^{T}(z_{1})I_{q}^{T}I_{q}R(z_{1})\right\}$.

By utilizing Lemma 2, the following results are obtained:


$$-\sum_{i=1}^{3}S_{1i}e_{1i}^{2r}\leq -{\overset{―}{S}}_{1m}{\left(\frac{1}{2}\sum_{i=1}^{3}S_{1i}e_{1i}^{2}\right)}^{r}$$ (55)

$$-k_{2}\sum_{i=1}^{3}S_{3i}e_{2i}^{2r}\leq -S_{3m}\sum_{i=1}^{3}e_{2i}^{2r}\leq -{\overset{―}{S}}_{3m}{\left(\frac{k_{2}}{2}\sum_{i=1}^{3}e_{2i}^{2}\right)}^{r}$$ (56)

where ${\bar{S}}_{1m}=2^{r}$, $S_{3m}=k_{2}\min\left\{S_{3}\right\}$, ${\bar{S}}_{3m}=S_{3m}2^{r}k_{2}^{-r}$.

According to Lemma 3, when taking $\sigma_{1}=\Pi_{1}$, $\sigma_{2}=1$, *b*_1_ = *r*, *b*_2_ = 1 − *r* and $b_{3}=\frac{1}{r}$, the following is obtained


$$-\Pi_{1}\leq -{\Pi_{1}}^{r}+(1-r){\frac{1}{r}}^{\frac{r}{1-r}}$$ (57)

Invoking equations (55), (56), and (57), equation (54) can be rewritten in the form


$$\begin{array}{cc} {\dot{V}}_{4}(t) & \leq -{\bar{S}}_{1m}{\left(\frac{1}{2}\sum_{i=1}^{3}S_{1i}e_{1i}^{2}\right)}^{r}-{\bar{S}}_{3m}{\left(\frac{k_{2}}{2}\sum_{i=1}^{3}e_{2i}^{2}\right)}^{r}-k_{5}^{r}\Lambda^{T}e_{2}^{T}e_{2}^{r-1}\Lambda^{r-1}\\ & \;-\frac{1}{2^{r}}k_{5}^{r}p_{L}^{r}\Lambda^{T}\Lambda^{2r-1}-C^{r}-\Pi_{1}^{r}+(1-r){\left(\frac{1}{r}\right)}^{\frac{r}{1-r}}+\Pi_{2}\\ & \leq -\Pi_{3}V_{4}^{r}(t)+\Pi_{4} \end{array}$$ (58)

where $\Pi_{3}=\min\{{\bar{S}}_{1m},{\bar{S}}_{3m},1\}$, and $\Pi_{4}=(1-r){\frac{1}{r}}^{\frac{r}{1-r}}+\Pi_{2}$.

The system exhibits finite-time convergence as established by Lemma 1. Furthermore, the upper bound of the convergence time is given by $T_{st}\leq \frac{1}{(1-r)\vartheta \delta_{1}}[V_{4}^{1-r}(x(0))-(\frac{\delta_{2}}{(1-\vartheta )\delta_{1}}{)}^{\frac{1-r}{r}}]$. The proof is completed.

**Remark 2.** In this section, the energy function method is employed to suppress the swing angle of the payload. In the aforementioned control design process, the energy function is used to construct the Lyapunov function. Through rigorous derivation and proof, it can be concluded that the Lyapunov function is monotonically decreasing, which means the energy function is also monotonically decreasing. Considered the definition of energy function, it is noted that it consists of the kinetic energy and potential energy of the payload. Hence, as the energy function decreases continuously, both the kinetic energy and potential energy gradually diminish, and the swing angles will eventually converge to an equilibrium state. This is precisely the principle by which the energy function method suppresses the swing angle of the payload.

**Remark 3.** In this section, the compensation function observer method is adopted to estimate the unknown disturbance. Compared with the traditional ESO (its specific design form is given in the simulation), the form of the error system (16)-(18) is a standard cascade system and it is more simple for the control design. More importantly, both $\lambda_{1}$ and $\lambda_{2}$ are utilized to observe the unknown disturbance. In other words, different from only using $\lambda_{1}$ in the traditional ESO-based appraoch, all system information is fully usage in (15). Hence, the developed compensation function observer has higher observation accuracy.

**Remark 4.** This work presents a unified robust finite-time anti-swing control framework for the QSLS by integrating the energy function strategy with compensation function observer. Unlike traditional anti-swing control methods, the developed approach addresses anti-swing, disturbance observation, and robust finite-time convergence within one integrated framework. Notably, by utilizing the intermediate variable *z*_0_ in the compensation function observer, the initial peaking phenomenon is effectively suppressed, and the robust finite-time convergence of the overall closed-loop system is theoretically established.

## 4 Simulation results

This section validates the control performance of the QSLS under the proposed control method. The physical parameters of the QSLS are directly adopted from established literature [14] to ensure that the evaluation is grounded in realistic and verified models. By referencing these well-documented parameters rather than arbitrary data, the evaluation maintains consistency with existing research and accurately reflects the dynamics of actual physical platforms. Specifically, the parameters are set as follows:


$$m_{Q}=0.468\;\;\;\;\;\text{kg},m_{L}=0.068\;\;\;\;\text{kg},p_{L}=0.9\;\;\;\text{m},g=9.81\;\;\;\text{m/}s^{2}$$

The initial swing angles, position and attitude angles of the QSLS are assumed as: ${x_{Q}}_{0}={y_{Q}}_{0}={z_{Q}}_{0}=0$ m, and $\alpha_{0}=\beta_{0}=0$ rad. The expected position of the quadrotor is selected as $\xi_{Qd}=[0.9\cos(0.2t),1+\sin(0.2t),2.5{]}^{T}$m. The external disturbance *d* are supposed as


$$d=\left[\begin{array}{c} 1.6\sin(0.3t)\\ 2.1\sin(0.4t)\\ 1.2\sin(0.02t) \end{array}\right]$$

Meanwhile, the selection of user-specified parameters is $\eta_{1}=0.1$, $\eta_{2}=2$, $N_{1}=\text{diag}\left\{2,2,2,2,2\right\}$, $N_{2}=\text{diag}\left\{20,20,20,20,20\right\}$, *S*_*Q*_ = 0.011, *S*_*L*_ = 26, *r* = 0.9, $S_{1}=\text{diag}\left\{15,12,10\right\}$, $S_{2}=\text{diag}\left\{2.1,1.85,4.5\right\}$.

The PID controller is designed as


$$F(t)=-s_{p1}e_{1}(t)-s_{i1}\int e_{1}(\tau )d\tau -s_{d1}{\dot{e}}_{1}(t)\;+s_{p2}e_{\Omega }(t)+s_{i2}\int e_{\Omega }(\tau )d\tau +s_{d2}{\dot{e}}_{\Omega }(t)$$

where $e_{\Omega }={\left[\alpha -\alpha_{d},\beta -\beta_{d}\right]}^{T}$, *s*_p1_, *s*_i1_, *s*_d1_, *s*_p2_, *s*_i2_ and *s*_d2_ are parameter matrices.

**Fig 3:**
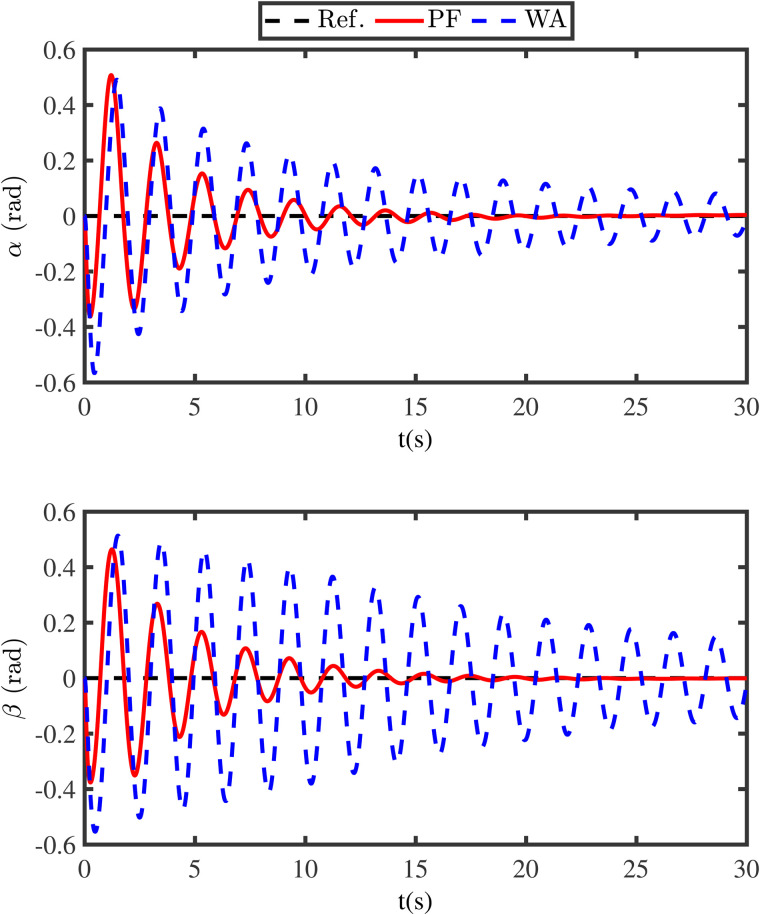
Tracking results of swing angles.

**Fig 4:**
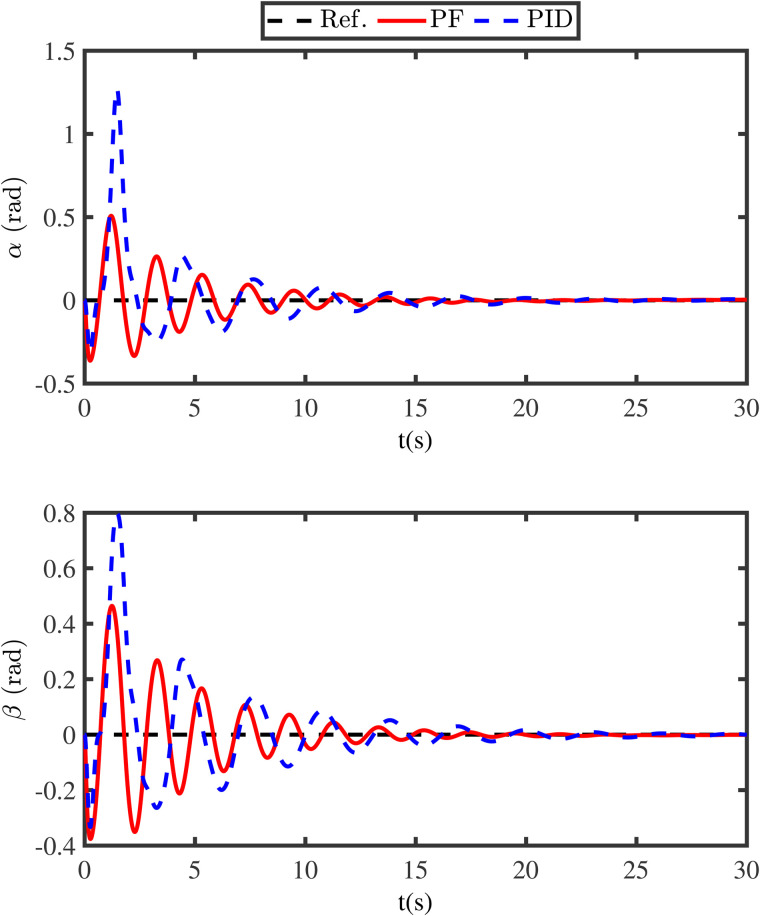
Comparison anti-swing results under the proposed energy function method and PID method.

**Fig 5:**
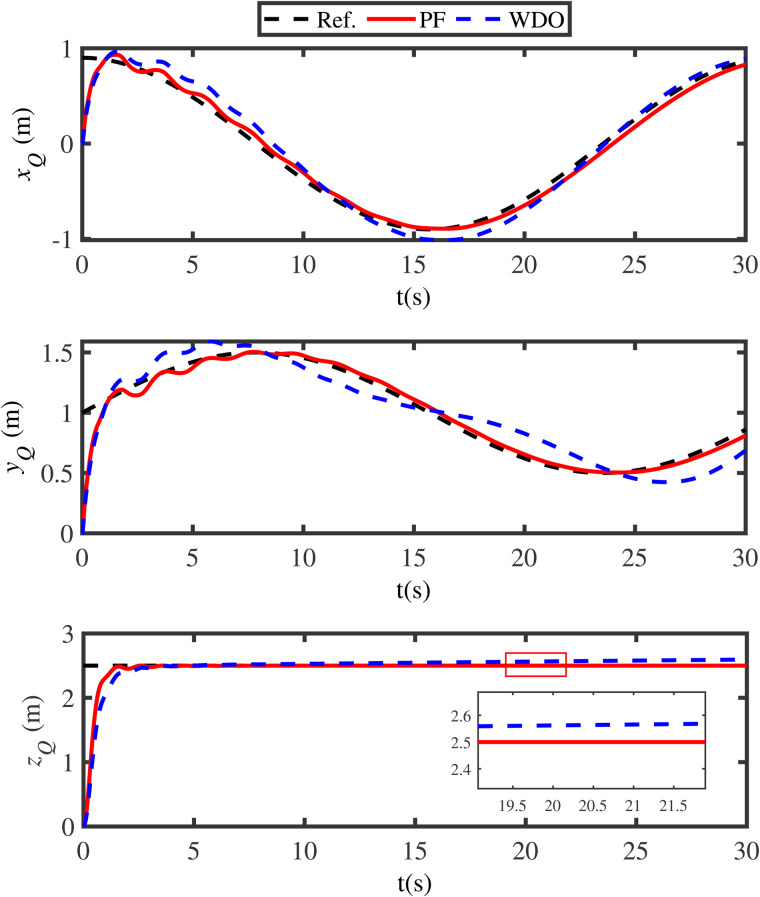
Tracking results of position loop.

**Fig 6:**
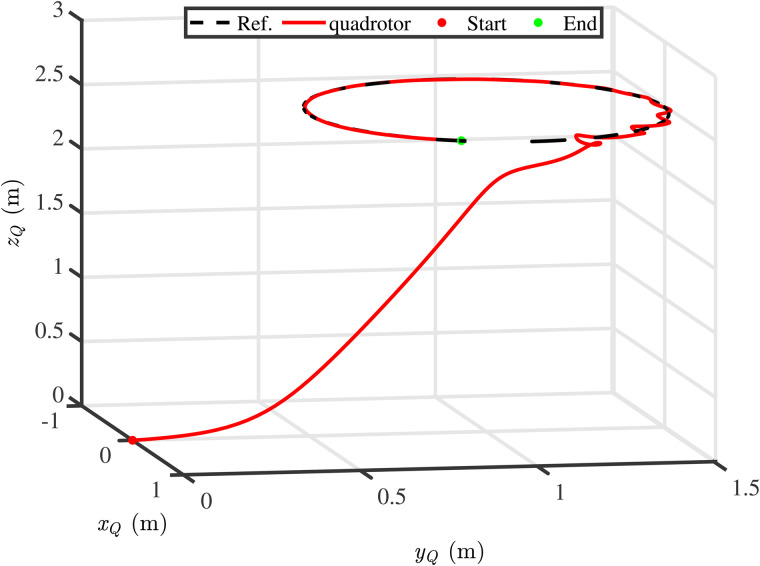
Three-dimensional tracking results of position loop.

**Fig 7:**
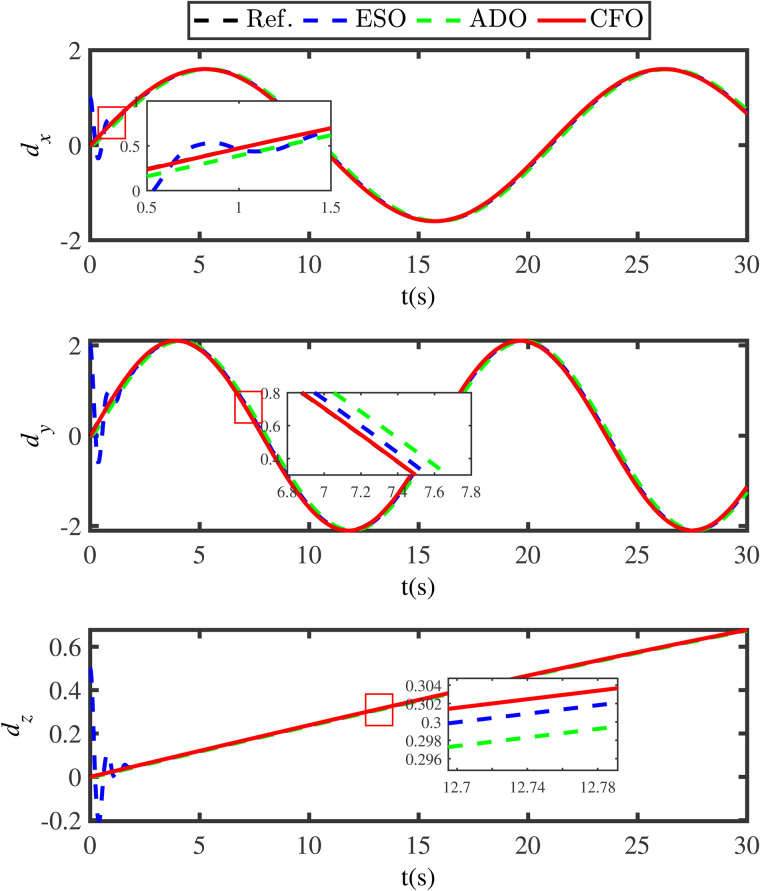
Comparison disturbance estimation results under different methods.

**Fig 8:**
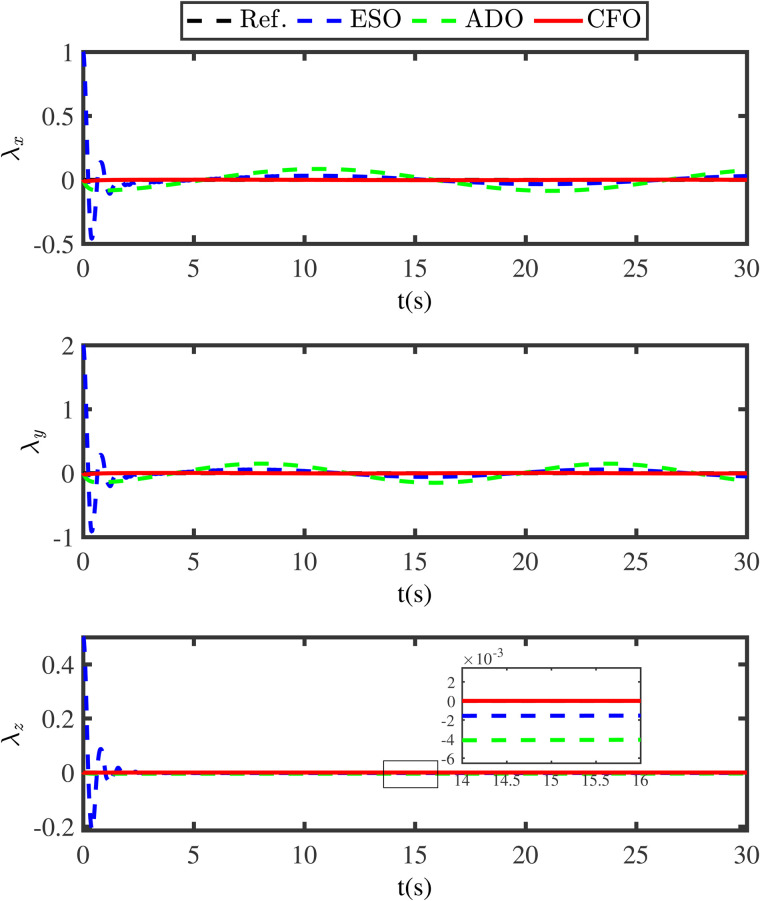
Comparison disturbance estimation errors under under different methods.

**Fig 9:**
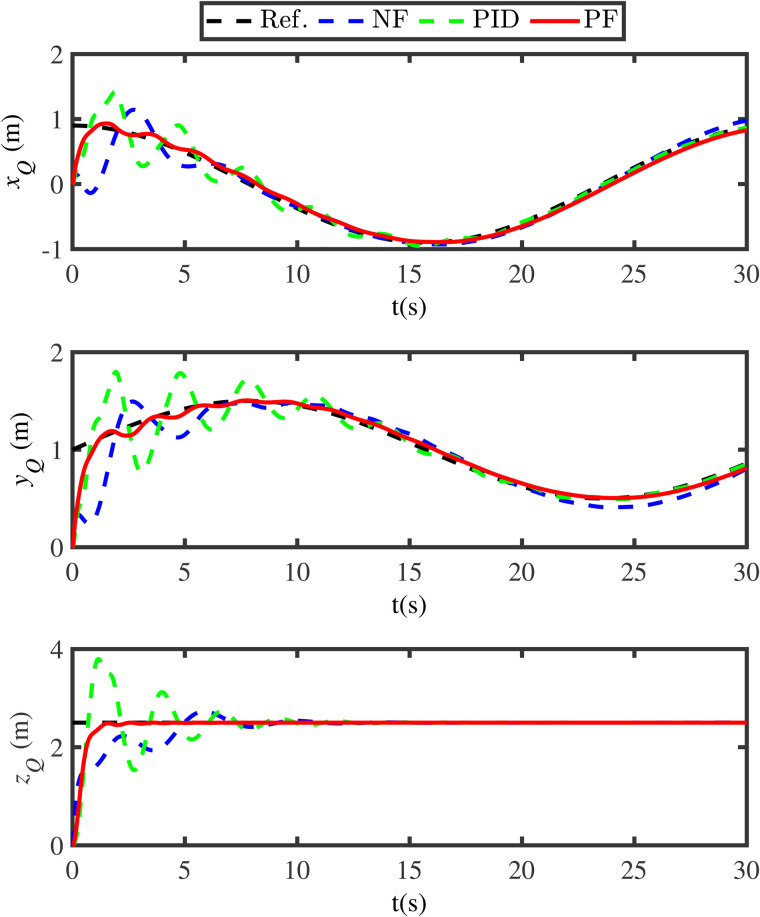
Comparison tracking results under the different control methods.

**Fig 10:**
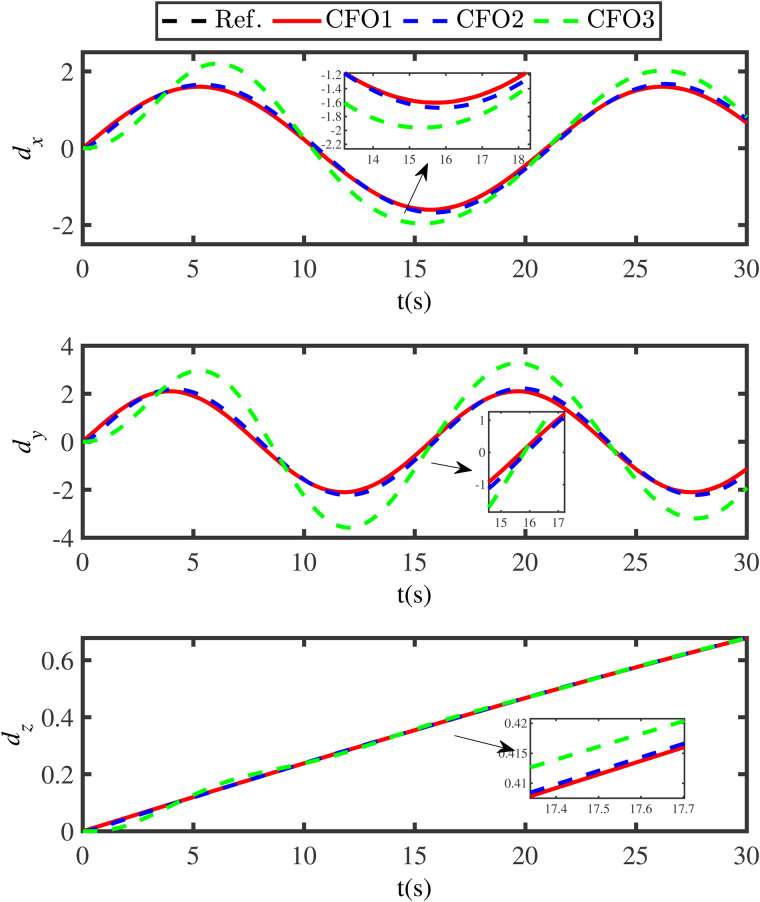
Comparison of compensation function observer tracking performance under different parameters.

**Fig 11:**
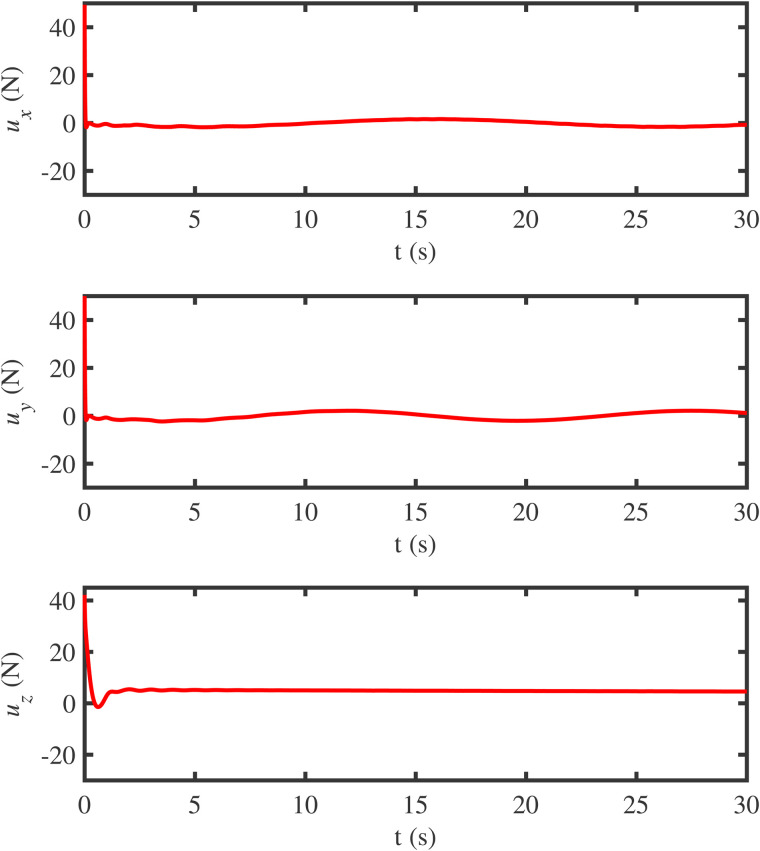
The curves of the finite-time anti-swing controller.

The simulation results are presented in Figs 3-11. Fig 3 shows the comparison results of whether the swing angles are suppressed, where the black lines (Ref.) represent the expected trajectories, the red lines (PF) represent the swing angles under the energy function method, and the blue lines (WA) denote the swing angles without control. From Fig 3, it can be seen that if the swing angles are not controlled, it will continue to swing left and right, which is detrimental to flight safety. However, under the proposed energy function-based method, the swing angles can converge to the neighborhood of zero in a short time. Meanwhile, in order to reveal the advantage of the energy function method, the comparison anti-swing results are provided in Fig 4, where the blue lines (PID) denote the swing angles under the PID controller. From Fig 4, it is observed that the proposed energy function-based method enables the swing angles to have a smaller overshoot and a faster convergence speed.

Fig 5 illustrates the impact of external unknown disturbances on the tracking performance of the QSLS, where the black lines (Ref.) represent the reference trajectories, the red lines (PF) refer to the actual trajectory under the proposed control scheme, and the blue lines (WDO) depict the tracking results with the disturbance being not suppressed. Fig 5 clearly demonstrates that external disturbances significantly degrade the safety performance of the QSLS. If the unknown disturbances cannot be addressed in a timely manner, the stability of the QSLS will be implicated. In addition, to present the control effect more clearly, the 3D tracking results of position loop are provided in Fig 6.

To further indicate the superiority of the developed compensation function observer, the ESO method and the adaptive disturbance observer (ADO) method are compared. The ESO is designed as


$$\left\{ \begin{array}{c} \lambda_{1}=z_{1}-{\hat{z}}_{1}\\ {\dot{\hat{z}}}_{1}={\hat{z}}_{2}+N_{s1}\lambda_{1}\\ {\dot{\hat{z}}}_{2}=R^{-1}(z_{1})(-J(z_{1},z_{2})z_{2}-M(z_{1})+H)+{\hat{z}}_{3}+N_{s2}\lambda_{1}\\ {\dot{\hat{z}}}_{3}=N_{s3}\lambda_{1} \end{array}\right.$$

where *N*_*s*1_, *N*_*s*2_ and *N*_*s*3_ are the designed parameters.

The ADO is designed as


$$\left\{ \begin{array}{c} {\hat{z}}_{1}={\hat{z}}_{2}\\ {\hat{z}}_{2}=R^{-1}(z_{1})(-J(z_{1},z_{2})z_{2}-M(z_{1})+H)+{\hat{z}}_{3}\\ {\hat{z}}_{3}=K\left(z_{2}-{\hat{z}}_{2}\right) \end{array}\right.$$

where *K* is the designed parameter.

Fig 7 gives the disturbance estimation comparison results of the ESO approach, the ADO approach and the proposed compensation function observer approach. Fig 8 shows the estimation errors under these different methods. In Figs 7 and 8, the black lines (Ref.) represent the actual disturbances, the blue lines (ESO) indicate the disturbance estimations under the ESO method, the green lines (ADO) denote the disturbance estimations under the ADO method, and the red lines (CFO) represent the disturbance estimations under the presented compensation function observer method. The comparative results in Figs 7 and 8 clearly demonstrate that the proposed compensation function observer method can ensure the QSLS have higher tracking accuracy and smaller tracking error. Moreover, to achieve satisfactory tracking result, the gain values of ESO are selected as $N_{s1}=\text{diag}\left\{250,250,250,250,250\right\}$, $N_{s2}=\text{diag}\left\{1380,1380,1380,1380,1380\right\}$, $N_{s3}=\text{diag}\left\{9000,9000,9000,9000,9000\right\}$. Obviously, the gain value of ESO is much larger than that of the compensation function observer. This also leads to the issue of initial peaking in the traditional ESO method.

Subsequently, Fig 9 compares the overall tracking performance of the QSLS under the proposed finite-time anti-swing control method, the conventional PID controller and traditional backstepping control method. In Fig 9, the black lines (Ref.) represent the reference trajectories, the blue lines (NF) represent the tracking errors under the energy function control method, the green lines (PID) represent the tracking errors under the PID controller, and the red lines (PF) denote the tracking errors under the proposed controller. From Fig 9, it is clear that the proposed finite-time control strategy has a faster convergence speed, which implies that the proposed control strategy can ensure the system has good transient performance.

Fig 10 illustrates the tracking performance of the compensation function observer under three different sets of gain parameters. Specifically, the first group (red solid line) is set as $\eta_{1}=0.1$, $\eta_{2}=2$, *N*_1_ = diag{2,2,2,2,2}, *N*_2_ = diag{20,20,20,20,20}; the second group (blue dashed line) is set as $\eta_{1}=0.1$, $\eta_{2}=0.2$, *N*_1_ = diag{0.2,0.2,0.2,0.2,0.2}, *N*_2_ = diag{2,2,2,2,2}; and the third group (green dashed line) is set as $\eta_{1}=0.1$, $\eta_{2}=0.2$, *N*_1_ = diag{0.2,0.2,0.2,0.2,0.2}, *N*_2_ = diag {0.2,0.2,0.2,0.2,0.2}. It can be observed that larger gain parameters lead to a better tracking effect and faster convergence, though at the cost of higher overshoot. This indicates that the current parameter selection relies on an empirical approach to balance tracking precision and transient stability. Further research on systematic parameter optimization will be conducted in future work.

The curves of the designed finite-time anti-swing controller are illustrated in Fig 11, which demonstrates the rapidity of the control performance. To sum up, the simulation results confirm that the designed controller significantly suppresses load swing and effectively ensures stable flight of the QSLS. It is worth noting that since the controller is based on analytical energy formulations, it avoids heavy online optimization and ensures a low computational cost. In MATLAB simulations, the computational load is minimal, and the system achieves stable convergence within 5s, guaranteeing a high potential for real-time implementation.

## 5 Conclusion

The finite-time anti-swing control problem has been investigated for the QSLS suffering from external disturbances. Firstly, based on comprehensive force analysis and kinematic constraints, the position loop nonlinear dynamics of the QSLS has been developed. Then, the compensation function observer has been designed to handle the unknown disturbances during the flight process. Finally, the energy function method has been combined with the finite-time theory to develop the robust anti-swing controller for the QSLS, ensuring the tracking performance and fast convergence of the whole system. Simulation results have demonstrated the feasibility and superior performance of the proposed method. In the future, the multi-constraint problem of the QSLS based on control barrier functions will be investigated, such as full state constraints and input saturation constraints. Furthermore, the real-world flight validation along with the associated parameter optimization method will be further explored.

## Supporting information

S1 File Supplementary information.zip: It contains the design parameters (Parameters.txt), the complete simulation source code, and a compressed package of all figures. (ZIP)
