# Supplementary material for: Robust finite-time anti-swing control for quadrotor slung-load system based on compensation function observer
Source: PLoS One. 2026 Apr 24;21(4):e0331662. doi: 10.1371/journal.pone.0331662 (PMC13108781; doi:10.1371/journal.pone.0331662)
Supplement: S1 File — (ZIP) [file pone.0331662.s001.zip › LaTeX source file (with figures)-bib/plos_latex_template.pdf]

Finite-time Robust Anti-swing Control for Quadrotor Slung-load System Based on Compensation Function Observer

Xiaoning Yu[1], Liaozhang Li[1], Kun Yan[1], Jiayong Fang[2‡], Peng Zhang[2], Bo Cheng[3,4].

1 College of Electronic Information Engineering, Xi'an Technological University, Xi'an, 710021, China
2 College of Equipment Management and UAV Engineering, Air Force Engineering University, Xi'an, 710051, China
3 Unmanned System Research Institute, Northwestern Polytechnical University, Xi'an, 710072, China
4 Nation Key Laboratory of Unmanned Aerial Vehicle Technology, Northwestern Polytechnical University, Xi'an, 710072, China

* correspondingauthor{fjylike@163.com}

Abstract

In this work, a finite-time robust anti-swing controller is proposed for the quadtrotor slung-load system suffering from external disturbances. In order to stabilize the swing angles of the slung-load, the energy function containing kinetic energy and potential energy is constructed such that the swing angles gradually converge to zero. Meanwhile, a novel compensation function observer are designed to suppress the unknown disturbances, which enhance the system robustness. Compared with the traditional extended state observer method, the constructed compensation function observer has faster convergence rate and better observation accuracy under smaller observer gain. For improving the fast convergence of the whole closed-loop system, the finite-time technique is utilized to design the robust anti-swing flight control scheme based on the Lyapunov stability theory and backstepping method, which ensures that all error signals of the quadrotor slung-load system are uniformly ultimately bounded. Finally, simulation verification is performed and some comparison results are provided to illustrate the effectiveness of the proposed control algorithm.

Keywords: Quadrotor slung-load system, Anti-swing control, Energy function approach, Compensation function observer, Finite-time

1 Introduction

In recent years, the quadrotor slung-load system (QSLS), which consists of a quadrotor unmanned aerial vehicle (UAV), a rigid string and a payload, has demonstrated significant application potential and scientific value in many areas, such as cargo transportation, cooperative positioning, forest-fire prevention and environmental monitoring [1–4]. However, the quadrotor UAV is a unique system characterized by underactuation and multivariable. When the payload is attached to the quadrotor by a rigid string, the complexity of the QSLS increases and the controllability needs to be enhanced [5]. Hence, as a top priority for improving the safety of the whole coupled system, anti-swing control has become a highly challenging research area.

Recent research has made significant progress in the swing angle stabilization of QSLS, with various effective control strategies having emerged in the existing literature, such as the trajectory planning method [6–8], the state-dependent Riccati equation method [9], the nonlinear geometric control method [10], the adaptive control method [11], and the energy function-based control method [12–16]. Among these methods, the energy function method has been extensively employed owing to its straightforward design process and effective anti-swing capability. In [12], an energy function-based nonlinear control approach was introduced to overcome the inherent underactuation and strong coupling characteristics of double-pendulum-load quadrotor systems. In [13], a dual-loop nonlinear control scheme integrating energy analysis with barrier Lyapunov functions was presented to achieve coordinated trajectory tracking and swing suppression. In [14], an asymptotic stabilization control method based on the energy function technique was established for precise positioning of the QSLS and payload swing angle regulation. In [15], a unified control framework based on energy function analysis was constructed to address the challenges of quadrotor positioning, payload swing elimination and vertical motion control simultaneously. In [16], the energy function was combined with the adaptive control method to handle the issue of variable tether length in QSLS. However, the detrimental influence of external disturbances on the suspension system has often been overlooked in much of the existing literature. Therefore, there is an urgent need to develop an efficient disturbance rejection scheme to ensure that the QSLS can operate safely and stably in complex environments.

In practice, the unknown disturbances including wind gust and air turbulence can directly affect the trajectory tracking accuracy and pose a significant threat to the flight performance of the QSLS. To address this difficulty, the active disturbance rejection control (ADRC) strategy pioneered by Han [17] provides an effective disturbance compensation framework. As a core technology of ADRC, extended state observer (ESO) has demonstrated its potential both theoretically and practically. In [18], a modified nonlinear ESO was designed for handling the affine nonlinearities in dynamic systems. In [19], the sliding mode control technology was combined with the ESO approach to tackle the unknown disturbances in underwater robot operations. In [20], a novel ESO with time-varying gains was introduced, effectively solving the robust safe control of

unmanned helicopter subjected to external disturbances. In [21], an ESO-based robust control strategy was designed for each subsystem of a fixed-wing UAV to improve its tracking performance and disturbance suppression ability. In [22], by adopting the ADRC technique, a feedback linearization controller was developed to cope with the model uncertainties and external disturbances in UAV systems. In [23], a cascaded ADRC framework was established for trajectory tracking control of quadrotor UAVs under external disturbances and model uncertainties. However, it is important to note that the estimation accuracy and convergence speed of the ESO are strongly influenced by its design parameters, which largely determine the overall performance of the system. In particular, the high gain tuning parameters often lead to the so-called "peaking phenomenon" in the initial stage. More critically, the traditional ESO design process does not make full use of all the system information, which also affects the final estimation accuracy. Therefore, exploring novel anti-disturbance strategy is undoubtedly a necessary and challenging task.

In addition, finite-time control can improve the rapidity of the system, which is exactly what the suspension system urgently need, and many valuable research achievements have been made in recent years [24–28]. In [24], a neural-based global finite-time convergence fault tolerance control strategy was proposed to identify and compensate for actuator faults of the unmanned helicopter. In [25], a finite-time stabilized adaptive fuzzy control approach was developed for nonlinear strict-feedback systems to enhance tracking performance. In [26], an adaptive finite-time tracking controller was designed for nonlinear systems under actuator faults. The application problem of finite-time attitude tracking control for unmanned helicopter was investigated in [27]. In [28], the adaptive sliding-mode observer was constructed to achieve the finite-time convergence of aerial manipulators, effectively compensating for both exogenous and endogenous uncertainties. However, when the problems of anti-swing and disturbance rejection are coupled simultaneously, the finite-time flight control for QSLS warrants further exploration.

Inspired by the above-mentioned research, this paper proposes a novel finite-time anti-swing control framework for the QSLS in the presence of external disturbances. The main contributions of this work are summarized as follows:

1) Compared with the traditional anti-swing approaches, the energy function method can not only simplify the controller design process but also improve the global convergence of the QSLS by integrating the swing angle information;

2) Compared with the traditional ESO approaches, the designed compensation function observer has a faster convergence rate and better observation accuracy under smaller observer gains, thereby enhancing the robustness of the QSLS;

3) The designed finite-time anti-swing control strategy can provide guaranteed bounded convergence for the QSLS while optimizing the transient behavior of the overall closed-loop system.

The remaining part of this paper is organized as follows. Section 2 derives the nonlinear dynamic model and theoretical foundations. Section 3 develops the finite-time anti-swing control framework, followed by comparative simulation analyses in Section 4. Finally, conclusions are summarized in Section 5.

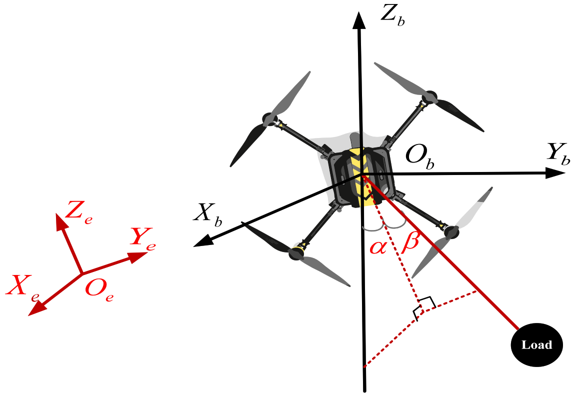

Fig 1. The structure of the QSLS

2 Problem Statement

The QSLS comprises a quadrotor UAV, a rigid string and a payload. In order to facilitate the understanding, the physical structure of the QSLS is shown in Fig. 1, where $E_e = \{X_e, Y_e, Z_e, O_e\}$ denotes the earth-fixed frame, $B_b = \{X_b, Y_b, Z_b, O_b\}$ denotes the body-fixed frame, O_e is located on the ground, O_b coincides with the centroid of the quadrotor, the angles α and β represent the swing angles of the payload, respectively.

The position loop nonlinear dynamics of the QSLS are formulated through the Newton-Euler principles as follows [11, 14]:

$$\begin{cases} m_Q \dot{V}_Q = -G_Q + U_1 + h_0 + d \\ m_L \dot{V}_L = -G_L - h_0 \end{cases} \tag{1}$$

where $G_Q = m_Q g \rho_s$, $G_L = m_L g \rho_s$, $\rho_s = [0, 0, 1]^T$, m_Q, m_L and g denote the mass of quadrotor, the mass of slung-load, and gravitational acceleration, respectively. $V_Q = [V_x, V_y, V_z]^T$ is the velocity vector of the quadrotor, $V_L = [v_x, v_y, v_z]^T$ is the velocity vector of the slung-load. $U_1 = [u_x, u_y, u_z]^T$ is the control input vector, $h_0 = [h_x, h_y, h_z]^T = [h \sin\alpha\cos\beta, h\sin\beta, -h\cos\alpha\cos\beta]^T$ is the cable tension vector in the body-fixed frame B_b, h is the tension of the string, and $d = [d_x, d_y, d_z]^T$ represents the external disturbance vector.

From Fig. 1, the spatial relationship between the quadrotor and slung-load can be mathematically expressed as

$$\xi_L = \xi_Q + p_L \Theta_a \tag{2}$$

where $\xi_Q = [x_Q, y_Q, z_Q]^T$ and $\xi_L = [x_L, y_L, z_L]^T$ denote the quadrotor and slung-load position vectors, respectively. p_L denotes the cable length, and $\Theta_a = [\Theta_{a1}, \Theta_{a2}, \Theta_{a3}]^T = [\sin\alpha\cos\beta, \sin\beta, -\cos\alpha\cos\beta]^T$.

Differentiating equation (2) twice with respect to time, the relative acceleration dynamics between the quadrotor and its slung-load can be derived as

$$\dot{V}_L = \dot{V}_Q + p_L \Theta_b \tag{3}$$

where $\Theta_b = \ddot{\Theta}_a = [\Theta_{b1}, \Theta_{b2}, \Theta_{b3}]^T$ with

$$\begin{cases} \Theta_{b1} = \ddot{\alpha}\cos\alpha\cos\beta - \ddot{\beta}\sin\alpha\sin\beta - \dot{\alpha}^2\sin\alpha\cos\beta \\ \qquad -\dot{\beta}^2\sin\alpha\cos\beta - 2\dot{\alpha}\dot{\beta}\cos\alpha\sin\beta \\ \Theta_{b2} = \ddot{\beta}\cos\beta - \dot{\beta}^2\sin\beta \\ \Theta_{b3} = \ddot{\alpha}\sin\alpha\cos\beta + \ddot{\beta}\cos\alpha\sin\beta + \dot{\alpha}^2\cos\alpha\cos\beta \\ \qquad +\dot{\beta}^2\cos\alpha\cos\beta - 2\dot{\alpha}\dot{\beta}\sin\alpha\sin\beta \end{cases} \tag{4}$$

According to equations (1) to (4), the kinematic equation of the QSLS is given by

$$\dot{V}_Q = \frac{U_1 - m_L p_L \Theta_b + d}{m_Q + m_L} - g\rho_s \tag{5}$$

Based on equations (1), (4), and (5), the swing angle constraint equations are established as follows:

$$\begin{cases} ((m_Q + m_L)\ddot{x}_Q - (u_x + d_x))\cos\alpha\cos\beta + ((m_Q + m_L)\ddot{z}_Q \\ -(u_z + d_z))\sin\alpha\cos\beta + m_L p_L(\ddot{\alpha}\cos^2\beta - 2\dot{\alpha}\dot{\beta}\sin\beta\cos\beta) = 0 \\ -((m_Q + m_L)\ddot{x}_Q - (u_x + d_x))\sin\alpha\sin\beta + ((m_Q + m_L)\ddot{y}_Q - (u_y + d_y))\cos\beta \\ +((m_Q + m_L)\ddot{z}_Q - (u_z + d_z))\cos\alpha\sin\beta + m_L p_L(\ddot{\beta} + \dot{\alpha}^2\sin\beta\cos\beta) = 0 \end{cases} \tag{6}$$

Furthermore, by integrating equations (1) to (6), we have

$$R(n)\ddot{n} + J(n, \dot{n})\dot{n} + M(n) = H + d' \tag{7}$$

where $H = [U_1 - (m_Q + m_L)g\rho_s, 0, 0]^T$ represents the resultant external force acting on the system, $n = [x_Q, y_Q, z_Q, \alpha, \beta]^T$ denotes the state vector of QSLS, $d' = [d, 0, 0]^T$. $R(n) \in R^{5\times5}$, $J(n, \dot{n}) \in R^{5\times5}$, and $M(n) \in R^5$ are defined as the system's inertia matrix, Coriolis coupling matrix, and gravity effect vector. Their mathematical formulations can be written as

$$R(n) = \begin{bmatrix} R_{11} & 0 & 0 & R_{14} & R_{15} \\ 0 & R_{22} & 0 & 0 & R_{25} \\ 0 & 0 & R_{33} & R_{34} & R_{35} \\ R_{41} & 0 & R_{43} & R_{44} & 0 \\ R_{51} & R_{52} & R_{53} & 0 & R_{55} \end{bmatrix} \tag{8}$$

$$J(n, \dot{n}) = \begin{bmatrix} 0 & 0 & 0 & J_{14} & J_{15} \\ 0 & 0 & 0 & 0 & J_{25} \\ 0 & 0 & 0 & J_{34} & J_{35} \\ 0 & 0 & 0 & J_{44} & J_{45} \\ 0 & 0 & 0 & J_{54} & 0 \end{bmatrix} \tag{9}$$

$$M(n) = [\ 0 \quad 0 \quad 0 \quad M_{14} \quad M_{15}\ ]^T \tag{10}$$

where $R_{11} = R_{22} = R_{33} = m_Q + m_L$, $R_{44} = m_L p_L^2 c_\beta^2$, $R_{55} = m_L p_L^2$, $R_{14} = R_{41} = m_L p_L c_\alpha c_\beta$, $R_{15} = R_{51} = -m_L p_L s_\alpha s_\beta$, $R_{34} = R_{43} = m_L p_L s_\alpha c_\beta$, $R_{25} = R_{52} = m_L p_L c_\beta$, $R_{35} = R_{53} = m_L p_L c_\alpha s_\beta$, $J_{14} = -m_L p_L (s_\alpha c_\beta \dot{\alpha} + c_\alpha s_\beta \dot{\beta})$, $J_{15} = -m_L p_L (s_\alpha c_\beta \dot{\beta} + c_\alpha s_\beta \dot{\alpha})$, $J_{25} = -m_L p_L s_\beta \dot{\beta}$, $J_{34} = m_L p_L (c_\alpha c_\beta \dot{\alpha} - s_\alpha s_\beta \dot{\beta})$, $J_{35} = m_L p_L (c_\alpha c_\beta \dot{\beta} - s_\alpha s_\beta \dot{\alpha})$, $J_{44} = -m_L p_L^2 s_\beta c_\beta \dot{\beta}$, $J_{45} = -m_L p_L^2 s_\beta c_\beta \dot{\alpha}$, $J_{54} = m_L p_L^2 s_\beta c_\beta \dot{\alpha}$, $M_{14} = m_L p_L g s_\alpha c_\beta$, $M_{15} = m_L p_L g c_\alpha s_\beta$. The abbreviations c_* and s_* represent $\cos(*)$ and $\sin(*)$ in this work, respectively.

The control objective of this work is to design an energy function-based finite-time controller capable of tracking the desired trajectory accurately despite the presence of unknown disturbances and restraining the swing motion of the slung-load efficiently. To attain this control objective, a series of essential assumptions and properties are introduced and utilized in the subsequent stages of development.

Assumption 1 [14,15]. For dynamic analysis, the suspension mechanism is supposed as massless and inelastic.

Assumption 2 [6,14]. The payload maintains a downward position relative to the quadrotor, and the swing angles are constrained by $|\alpha| < \frac{\pi}{2}, |\beta| < \frac{\pi}{2}$.

Assumption 3 [28,29]. There exist two positive scalars d_ρ and d_σ such that

$$\|d\| \leq d_\rho, \left\|\dot{d}\right\| \leq d_\sigma \tag{11}$$

Property 1 [6,30,31]. The inertia matrix $R(n)$ possesses invertibility and positive definiteness, with a positive bound R_q satisfying $\left\|R^{-1}(n)\right\| \leq R_q$.

Remark 1. In the domain of quadrotor slung-load transportation mission, the string is rigid and the payload is consistently positioned beneath the quadrotor. This configuration is intentionally employed to reduce the likelihood of collisions with the quadrotor, thereby ensuring comprehensive flight safety. If the payload swings above the body of the quadrotor or becomes parallel to it, this will be inconsistent with real-world scenarios. Hence, within the framework of practical engineering systems, Assumption 2 is logically reasonable and can be well-founded in many existing literatures [6,14,15,30,31]. Furthermore, the energy of external disturbances is limited in practice. Otherwise, the controller will be unable to provide sufficient energy to counteract them. Therefore, Assumption 3 is reasonable for practical industrial system [28,29].

3 Main results

During the actual flight of the QSLS, external disturbances pose a substantial threat to flight safety. Therefore, this section introduces a compensation function observer to precisely compensate for these disturbances. Furthermore, an anti-swing control strategy founded on the energy function approach is proposed to deal with the intricate coupling dynamics between the quadrotor and its slung-load. Ultimately, the finite-time approach is employed to improve the

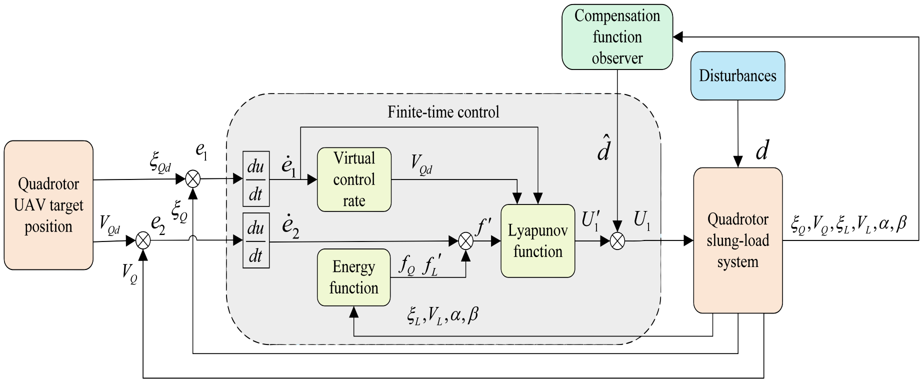

Fig 2. Architecture of the proposed control scheme

convergence speed of the QSLS. The structure of the overall control scheme is illustrated in Fig. 2.

3.1 Design of Compensation Function Observer

By reformulating equation (7), this model is adapted to support further analysis. Designing state variables $z_1 = n$ and $z_2 = \dot{n}$, it gives

$$\begin{cases} \dot{z}_1 = z_2 \\ R(z_1)\dot{z}_2 + J(z_1, z_2)z_2 + M(z_1) = H + d' \end{cases} \tag{12}$$

According to Property 1, equations (12) can be further represented as

$$\begin{cases} \dot{z}_1 = z_2 \\ \dot{z}_2 = R^{-1}(z_1)(-J(z_1, z_2)z_2 - M(z_1) + H) + D \end{cases} \tag{13}$$

where $D = R^{-1}(z_1)d'$.

In accordance with Assumption 3, it is known that D is bounded. Then, considering D as a new state z_3 with $\dot{D} = \varepsilon_d$, we have

$$\begin{cases} \dot{z}_1 = z_2 \\ \dot{z}_2 = R^{-1}(z_1)(-J(z_1, z_2)z_2 - M(z_1) + H) + z_3 \\ \dot{z}_3 = \varepsilon_d \end{cases} \tag{14}$$

According to equations (14), the compensation function observer for the QSLS is proposed as

$$\begin{cases} \dot{\hat{z}}_1 = \hat{z}_2 \\ \dot{\hat{z}}_2 = R^{-1}(z_1)(-J(z_1, z_2)z_2 - M(z_1) + H) + \hat{z}_3 \\ \dot{\hat{z}}_3 = \eta_1 N_1 \lambda_1 + (\eta_2 N_2 + N_1)\lambda_2 + N_2 \lambda_3 \\ \hat{z}_3 = z_0 + N_1 \lambda_1 + N_2 \lambda_2 \\ \dot{z}_0 = \eta_1 N_1 \lambda_1 + \eta_2 N_2 \lambda_2 \end{cases} \tag{15}$$

where $\hat{z}_1$, $\hat{z}_2$ and $\hat{z}_3$ are the estimates of z_1, z_2 and z_3, respectively. z_0 is the intermediate variable, $\eta_1 > 0$ and $\eta_2 > 0$ are the positive constants, $N_1 \in R^{5\times5}$ and $N_2 \in R^{5\times5}$ are the designed positive definite matrixes. $\lambda_1 = z_1 - \hat{z}_1$, $\lambda_2 = z_2 - \hat{z}_2$ and $\lambda_3 = z_3 - \hat{z}_3$ are the estimation errors of z_1, z_2 and z_3, respectively.

Differentiating $\lambda_i (i = 1, 2, 3)$ with respect to time gives

$$\dot{\lambda}_1 = \dot{z}_1 - \dot{\hat{z}}_1 = z_2 - \hat{z}_2 = \lambda_2 \tag{16}$$

$$\dot{\lambda}_2 = \dot{z}_2 - \dot{\hat{z}}_2 = z_3 - \hat{z}_3 = \lambda_3 \tag{17}$$

$$\begin{aligned}\dot{\lambda}_3 &= \varepsilon_d - \eta_1 N_1 \lambda_1 - \eta_2 N_2 \lambda_2 - N_1 \dot{\lambda}_1 - N_2 \dot{\lambda}_2 \\ &= \varepsilon_d - \eta_1 N_1 \lambda_1 - \eta_2 N_2 \lambda_2 - N_1 \lambda_2 - N_2 \lambda_3 \\ &= -\eta_1 N_1 \lambda_1 - (\eta_2 N_2 + N_1)\lambda_2 - N_2 \lambda_3 + \varepsilon_d \end{aligned} \tag{18}$$

By defining $\Upsilon = [\lambda_1^T, \lambda_2^T, \lambda_3^T]^T$, the following relationship is obtained

$$\begin{aligned}\dot{\Upsilon} &= \begin{bmatrix} 0_{5\times5} & I_{5\times5} & 0_{5\times5} \\ 0_{5\times5} & 0_{5\times5} & I_{5\times5} \\ -\eta_1 N_1 & -(\eta_2 N_2 + N_1) & -N_2 \end{bmatrix} \Upsilon + \begin{bmatrix} 0_{5\times1} \\ 0_{5\times1} \\ \varepsilon_d \end{bmatrix} \\ &= \varpi_\lambda \Upsilon + \varepsilon_e \end{aligned} \tag{19}$$

where the subscripts 5×5 and 5×1 specify the dimensional information of matrix. $0_{5\times5}$ is the zero matrix and $I_{5\times5}$ is the identity matrix. Moreover, the bounded ε_d implies ε_e is also bounded.

Here, by selecting parameters properly, ϖ_λ can be guaranteed to be a Hurwitz matrix. This implies the existence of a positive definite matrix Q_λ verifying

$$\varpi_\lambda^T Q_\lambda + Q_\lambda \varpi_\lambda = -W_\lambda \tag{20}$$

where W_λ is the positive definite matrix.

The proposed Lyapunov function candidate is given by

$$V_1(t) = \Upsilon^T Q_\lambda \Upsilon \tag{21}$$

By invoking equations (19) to (20), we can obtain

$$\begin{aligned}\dot{V}_1(t) &= \Upsilon^T Q_\lambda (\varpi_\lambda \Upsilon + \varepsilon_\lambda) + (\varpi_\lambda \Upsilon + \varepsilon_e)^T Q_\lambda \Upsilon \\ &= \Upsilon^T (Q_\lambda \varpi_\lambda + \varpi_\lambda^T Q_\lambda)\Upsilon + 2\Upsilon^T Q_\lambda \varepsilon_e \\ &\leq -\Upsilon^T (W_\lambda - I_{15\times15})\Upsilon + \|Q_\lambda \varepsilon_e\|^2 \\ &\leq -\tau_1 \Upsilon^T \Upsilon + \varepsilon_{dm} \end{aligned} \tag{22}$$

where $\tau_1 = \lambda_{\min}(W_\lambda - I_{15\times15})$ and $\varepsilon_{dm} = \|Q_\lambda \varepsilon_e\|^2$.

3.2 Design of Finite-time Anti-Swing Control

To streamline the controller design process, the following lemmas are presented

Lemma 1 [27, 28]. Consider the positional loop equations for QSLS as equations (5) and (6). If there is a smooth positive definite function $V(x)$ such that $\dot{V}(x) \leq -\delta_1 V^r(x) + \delta_2$ always holds, where $\delta_1 > 0$, $0 < r < 1$ and $0 < \delta_2 < \infty$ are real numbers, then the position of QSLS is finite-time stable. Meanwhile, the settling time T_{st} can be computed by $T_{st} \leq \frac{1}{(1-r)\vartheta\delta_1}[V^{1-r}(x(0)) - (\frac{\delta_2}{(1-\vartheta)\delta_1})^{\frac{1-r}{r}}]$ with $0 < \vartheta < 1$ being a constant.

Lemma 2 [24, 30]. For any real numbers $\varepsilon_i (i = 1, 2, \ldots, n)$, the following relationship holds

$$\begin{cases} (\sum\limits_{i=1}^{n} |\varepsilon_i|)^{\nu} \leq \sum\limits_{i=1}^{n} |\varepsilon_i|^{\nu} \\ (\sum\limits_{i=1}^{n} |\varepsilon_i|^2)^{\rho} \leq (\sum\limits_{i=1}^{n} |\varepsilon_i|^{\rho})^2 \end{cases} \tag{23}$$

where $0 < \nu < 1$ and $0 < \rho < 2$ are constants.

Lemma 3 [24]. For any set of positive constants b_1, b_2 and b_3, the following inequation holds

$$|\sigma_1|^{b_1} |\sigma_2|^{b_2} \leq \frac{b_1}{b_1 + b_2} b_3 |\sigma_1|^{b_1+b_2} + \frac{b_2}{b_1 + b_2} b_3^{\frac{b_1}{b_2}} |\sigma_2|^{b_1+b_2} \tag{24}$$

where σ_1 and σ_2 are real variables.

Based on equation (5), the following tracking error vectors are defined:

$$e_1 = \xi_Q - \xi_{Q_d} \tag{25}$$

$$e_2 = V_Q - V_{Q_d} \tag{26}$$

Taking the derivative of e_1 yields

$$\dot{e}_1 = \dot{\xi}_Q - \dot{\xi}_{Q_d} = e_2 + V_{Q_d} - \dot{\xi}_{Q_d} \tag{27}$$

The virtual control law is designed as

$$V_{Q_d} = \dot{\xi}_{Q_d} - e_1^{2r-1} \tag{28}$$

where $e_1^{2r-1} = \left[e_{11}^{2r-1}, e_{12}^{2r-1}, e_{13}^{2r-1}\right]^T$, $r \in (0, 1)$ is a designed constant.

Substituting equation (28) into equation (27) yields

$$\dot{e}_1 = e_2 - e_1^{2r-1} \tag{29}$$

The Lyapunov function candidate is selected as

$$V_2(t) = \frac{1}{2} e_1^T S_1 e_1 \tag{30}$$

where $S_1 = \text{diag}\{S_{11}, S_{12}, S_{13}\}$ is the designed positive definite diagonal matrix.

Taking the derivative of $V_2(t)$ and invoking equation (29) yields

$$\dot{V}_2(t) = e_1^T S_1 \dot{e}_1 = e_1^T S_1 e_2 - e_1^T S_1 e_1^{2r-1} \tag{31}$$

To simultaneously achieve trajectory tracking and anti-swing control of the QSLS, a finite-time swing rejection control scheme is derived from the energy function of the QSLS. First, the kinetic energy of the quadrotor is defined as

$$f_Q = \frac{1}{2}m_Q(\dot{x}_Q^2 + \dot{y}_Q^2 + \dot{z}_Q^2) \tag{32}$$

Differentiating equation (32) yields

$$\dot{f}_Q = \dot{x}_Q\left(m_Q\ddot{x}_Q\right) + \dot{y}_Q\left(m_Q\ddot{y}_Q\right) + \dot{z}_Q\left(m_Q\ddot{z}_Q\right) \tag{33}$$

Subsequently, the kinetic energy of slung-load is expressed as

$$f_L = \frac{1}{2}m_L(\dot{x}_L^2 + \dot{y}_L^2 + \dot{z}_L^2) \tag{34}$$

Taking the derivative of f_L gives

$$\dot{f}_L = \dot{x}_L\left(m_L\ddot{x}_L\right) + \dot{y}_L\left(m_L\ddot{y}_L\right) + \dot{z}_L\left(m_L\ddot{z}_L\right) \tag{35}$$

Differentiating equation (2) yields

$$\dot{\xi}_L = \dot{\xi}_Q + \Lambda \tag{36}$$

where

$$\Lambda = \begin{bmatrix} \Lambda_x \\ \Lambda_y \\ \Lambda_z \end{bmatrix} = p_L\dot{\Theta}_a = \begin{bmatrix} p_L(\cos\alpha\cos\beta\dot{\alpha} - \sin\alpha\sin\beta\dot{\beta}) \\ p_L\cos\beta\dot{\beta} \\ -p_L(\sin\alpha\cos\beta\dot{\alpha} + \cos\alpha\sin\beta\dot{\beta}) \end{bmatrix}.$$

Substituting equations (1) and (36) into equation (35), it gives

$$\begin{aligned} \dot{f}_L = {} & \dot{x}_Q\left(-h\sin\alpha\cos\beta\right) + \dot{y}_Q\left(-h\sin\beta\right) + \dot{z}_Q\left(-h\cos\alpha\cos\beta\right) \\ & - m_L g p_L\left(\sin\alpha\cos\beta\dot{\alpha} + \cos\alpha\sin\beta\dot{\beta}\right) \end{aligned} \tag{37}$$

Then, the position of the slung-load is defined as the zero potential energy plane when the swing angle is set to zero. Consequently, the potential energy equation of the slung-load can be expressed as follows:

$$f_{Lp} = p_L m_L g(1 - \cos\alpha\cos\beta) \tag{38}$$

The total energy function f'_L containing equations (34) and (38) is constructed as

$$\begin{aligned} f'_L &= f_L + f_{Lp} \\ &= \frac{1}{2}m_L(\dot{x}_L^2 + \dot{y}_L^2 + \dot{z}_L^2) + p_L m_L g(1 - \cos\alpha\cos\beta) \end{aligned} \tag{39}$$

By differentiating equation (39), it can be obtained that

$$\dot{f}'_L = \dot{x}_Q\left(-h\sin\alpha\cos\beta\right) + \dot{y}_Q\left(-hsin\beta\right) + \dot{z}_Q\left(-h\cos\alpha\cos\beta\right) \tag{40}$$

Invoking equations (32) and (39), the complete energy function is constructed as

$$\begin{aligned} f &= S_Q f_Q + S_L f_L' \\ &= \frac{1}{2} S_Q m_Q (\dot{x}_Q^2 + \dot{y}_Q^2 + \dot{z}_Q^2) + \frac{1}{2} S_L m_L (\dot{x}_L^2 + \dot{y}_L^2 + \dot{z}_L^2) \\ &\quad + S_L p_L m_L g (1 - \cos\alpha \cos\beta) \end{aligned} \tag{41}$$

where $S_Q > 0$ and $S_L > 0$ are energy coefficients.

Finally, in order to combine the energy function with the velocity error of the QSLS, an auxiliary energy function is defined as

$$\begin{aligned} f_d = &\frac{1}{2} S_Q m_Q \left(V_{xd}^2 + V_{yd}^2 + \dot{V}_{zd}^2 \right) + \frac{1}{2} S_L m_L \left(v_{xd}^2 + v_{yd}^2 + v_{zd}^2 \right) \\ &- S_Q m_Q \left(V_x V_{xd} + V_y V_{yd} + V_z V_{zd} \right) - S_L m_L \left(v_x v_{xd} + v_y v_{yd} + v_z v_{zd} \right) \end{aligned} \tag{42}$$

Combining it with equation (41) to form a new energy function as follows:

$$\begin{aligned} f' = f + f_d &= \frac{1}{2} S_Q m_Q (\dot{x}_Q^2 + \dot{y}_Q^2 + \dot{z}_Q^2) + \frac{1}{2} S_Q m_Q (V_{xd}^2 + V_{yd}^2 + \dot{V}_{zd}^2) \\ &\quad - S_Q m_Q (V_x V_{xd} + V_y V_{yd} + V_z V_{zd}) + \frac{1}{2} S_L m_L (\dot{x}_L^2 + \dot{y}_L^2 + \dot{z}_L^2) \\ &\quad + \frac{1}{2} S_L m_L (v_{xd}^2 + v_{yd}^2 + v_{zd}^2) - S_L m_L (v_x v_{xd} + v_y v_{yd} + v_z v_{zd}) \\ &\quad + S_L p_L m_L g (1 - \cos\alpha \cos\beta) \\ &= \frac{1}{2} S_Q m_Q (V_x^2 + V_y^2 + V_z^2) + \frac{1}{2} S_Q m_Q (V_{xd}^2 + V_{yd}^2 + \dot{V}_{zd}^2) \\ &\quad - S_Q m_Q (V_x V_{xd} + V_y V_{yd} + V_z V_{zd}) + \frac{1}{2} S_L m_L (v_x^2 + v_y^2 + v_z^2) \\ &\quad + \frac{1}{2} S_L m_L (v_{xd}^2 + v_{yd}^2 + v_{zd}^2) - S_L m_L (v_x v_{xd} + v_y v_{yd} + v_z v_{zd}) \\ &\quad + S_L p_L m_L g (1 - \cos\alpha \cos\beta) \\ &= \frac{1}{2} S_Q m_Q e_2^T e_2 + \frac{1}{2} S_L m_L L^T L + S_L p_L m_L g (1 - \cos\alpha \cos\beta) \end{aligned} \tag{43}$$

where $L = V_L - V_{Ld}$.

By Assumption 2, the inequality $(1 - \cos\alpha \cos\beta) \geq 0$ holds, which implies that f' is a positive definite function.

The relationship between the desired trajectories of the slung-load and the quadrotor is given by

$$V_{Qd} = V_{Ld} \tag{44}$$

Finally, since there is no term directly related to L in the QSLS model, it is necessary to transform the term. By invoking equations (36) and (44), equation

(43) can be rewritten as follows:

$$f' = \frac{1}{2}\left(S_Q m_Q + S_L m_L\right) e_2^T e_2 + S_L m_L p_L \Lambda^T e_2$$
$$+ S_L p_L m_L g(1 - \cos\alpha\cos\beta) + \frac{1}{2} S_L m_L p_L^2 \Lambda^T \Lambda \tag{45}$$

From equations (1), (33), (36), (40), and (44), the derivative of f' is given by

$$\begin{aligned}
\dot{f}' &= S_Q e_2^T (m_Q \dot{V}_Q - m_Q \dot{V}_{Qd}) + S_L L^T (m_L \dot{V}_L - m_L \dot{V}_{Ld}) \\
&\quad + S_L p_L m_L g(\sin\alpha\cos\beta\dot{\alpha} + \cos\alpha\sin\beta\dot{\beta}) \\
&= S_Q e_2^T (m_Q \dot{V}_Q - m_Q \dot{V}_{Qd}) + S_L (e_2 + \Lambda)^T (-G_L - h_0 - m_L \dot{V}_d) \\
&\quad + S_L p_L m_L g(\sin\alpha\cos\beta\dot{\alpha} + \cos\alpha\sin\beta\dot{\beta}) \\
&= S_Q e_2^T (m_Q \dot{V}_Q - m_Q \dot{V}_{Qd}) - S_L m_L \Lambda^T \dot{V}_d \\
&\quad + S_L e_2^T (-G_L - G_Q + U_1 + d - m_Q \dot{V}_Q - m_L \dot{V}_{Ld}) \\
&= e_2^T ((S_Q - S_L) m_Q \dot{V}_Q - S_L(-G_L - G_Q + U_1 + d - m_L \dot{V}_{Ld})) \\
&\quad - S_L m_L \Lambda^T \dot{V}_d
\end{aligned} \tag{46}$$

By substituting equation (5) into equation (46), the following result can be obtained

$$\begin{aligned}
\dot{f}' &= e_2^T \Big(\frac{m_Q S_Q + m_L S_L}{m_Q + m_L} U_1 - \frac{(S_Q - S_L) m_Q m_L p_L}{m_Q + m_L} \Theta_b - (S_Q m_Q \\
&\quad + S_L m_L) g \rho_s + \frac{m_Q S_Q + m_L S_L}{m_Q + m_L} d - (m_Q S_Q + m_L S_L) \dot{V}_{Qd}\Big) \\
&\quad - S_L m_L \Lambda^T \dot{V}_d
\end{aligned} \tag{47}$$

The estimate $\hat{z}_3$ of z_3 is obtained from the compensation function observer designed in the previous section, which is related to $\hat{d}$ as

$$\hat{d} = I_q R(z_1) \hat{z}_3 \tag{48}$$

where $I_q = \begin{bmatrix} 1 & 0 & 0 & 0 & 0 \\ 0 & 1 & 0 & 0 & 0 \\ 0 & 0 & 1 & 0 & 0 \end{bmatrix}$.

Then, based on the QSLS position model (5) and the energy function (45), the finite-time anti-swing controller is designed as follows:

$$\begin{aligned}
U_1 = k_1 \Big(&-k_2 S_2 e_2^{2r-1} + k_3 m_Q m_L u_L \Theta_b - k_4 \hat{d}_1 - \frac{1}{2} k_4^2 e_2 \\
&+ k_2 g \rho_s - S_1 e_1 + k_2 \dot{V}_{Qd} - \frac{1}{2} \frac{e_2 m_L^2 S_L^2 \Lambda^T \Lambda}{e_2^T e_2}\Big)
\end{aligned} \tag{49}$$

where $e_2^{2r-1} = \left[e_{21}^{2r-1}, e_{22}^{2r-1}, e_{23}^{2r-1}\right]^T$, $k_1 = \frac{m_Q + m_L}{S_Q m_Q + S_L m_L}$, $k_2 = S_Q m_Q + S_L m_L$, $k_3 = \frac{(S_Q - S_L)}{m_Q + m_L}$, $k_4 = \frac{1}{k_1}$, $S_2 = \operatorname{diag}\{S_{21}, S_{22}, S_{23}\}$ is the designed positive definite matrix.

Selecting the Lyapunov function candidate as

$$V_3(t) = V_2(t) + f' \tag{50}$$

By substituting equation (45) into equation (50), the following result can be obtained

$$V_3(t) = \frac{1}{2}e_1^T S_1 e_1 + \frac{1}{2}k_2 e_2^T e_2 + k_5 \Lambda^T e_2 + \frac{1}{2}k_5 p_L \Lambda^T \Lambda + C \tag{51}$$

where $C = k_5 g(1 - \cos\alpha\cos\beta)$, $k_5 = S_L m_L p_L$.

Finally, by invoking equations (31), (47) and (49), the derivative of equation (51) can be reformulated as

$$\begin{aligned}
\dot{V}_3(t) &= \dot{V}_2(t) + \dot{f}' \\
&= e_1^T S_1(e_2 - e_1^{2r-1}) + e_2^T(\frac{m_Q S_Q + m_L S_L}{m_Q + m_L}U_1 - \frac{(S_Q - S_L)m_Q m_L p_L}{m_Q + m_L}\Theta_b) \\
&- (S_Q m_Q + S_L m_L)g\rho_s + \frac{m_Q S_Q + m_L S_L}{m_Q + m_L}d - (m_Q S_Q + m_L S_L)\dot{V}_{Qd} \\
&- \frac{1}{2}\frac{e_2 m_L^2 S_L^2 \Lambda^T \Lambda}{e_2^T e_2}) - S_L m_L \Lambda^T \dot{V}_{Qd} \\
&\leq e_1^T S_1 e_2 - e_1^T S_1 e_1^{2r-1} + \frac{1}{2}\dot{V}_{Qd}^T \dot{V}_{Qd} + e_2^T(-k_2 S_2 e_2^{2r-1} - S_1 e_1 \\
&+ k_4 \tilde{d}_1 - \frac{1}{2}k_4^2 e_2) \\
&\leq -e_1^T S_1 e_1^{2r-1} - k_2 e_2^T S_2 e_2^{2r-1} + \frac{1}{2}\tilde{d}_1^T \tilde{d}_1 + \varepsilon_{Qd}
\end{aligned} \tag{52}$$

where $\tilde{d}_1 = (d_1 - \hat{d}_1)$, $\varepsilon_{Qd} = \frac{1}{2}\dot{V}_{Qd}^T \dot{V}_{Qd}$.

3.3 Stability Analysis

To summarize the core contributions, the following theorem is presented:

Theorem 1. *Consider the disturbed QSLS nonlinear model (7). The compensation function observer is designed (15). By applying the robust finite-time anti-swing control strategy (49), the swing angles of the slung-load can be suppressed efficiently and all error signals are guaranteed to be bounded, enabling the system output to track the desired trajectory.*

Proof. Consider the Lyapunov function candidate as

$$\begin{aligned}
V_4(t) &= V_1(t) + V_3(t) \\
&= \Upsilon^T Q_\lambda \Upsilon + \frac{1}{2}e_1^T S_1 e_1 + \frac{1}{2}k_2 e_2^T e_2 \\
&+ k_5 \Lambda^T e_2 + \frac{1}{2}k_5 p_L \Lambda^T \Lambda + C
\end{aligned} \tag{53}$$

Invoking equations (22), (31) and (52), the time derivative of $V_4(t)$ is given by

$$
\begin{aligned}
\dot{V}_4(t) &= \dot{V}_1(t) + \dot{V}_3(t) \\
&\le -\tau_1 \Upsilon^T \Upsilon + \varepsilon_{dm} - \sum_{i=1}^{3} S_{1i} e_{1i}^{2r} - k_2 \sum_{i=1}^{3} S_{2i} e_{2i}^{2r} + \varepsilon_{Qd} \\
&+ \frac{1}{2} \tilde{d}_1^T \tilde{d}_1 \ - k_5^r \Lambda^T e_2^T e_2^{r-1} \Lambda^{r-1} + k_5^r \Lambda^T e_2^T e_2^{r-1} \Lambda^{r-1} \\
&- C^r + C^r - \frac{1}{2^r} k_5^r p_L^r \Lambda^T \Lambda^{2r-1} + \frac{1}{2^r} k_5^r p_L^r \Lambda^T \Lambda^{2r-1} \\
&\le -\tau_1 \Upsilon^T \Upsilon + \varepsilon_{dm} - \sum_{i=1}^{3} S_{1i} e_{1i}^{2r} - k_2 \sum_{i=1}^{3} S_{2i} e_{2i}^{2r} \\
&+ \frac{1}{2} \tilde{d}_1^T \tilde{d}_1 + \frac{1}{2} e_2^T e_2^{2r-1} \ - k_5^r \Lambda^T e_2^T e_2^{r-1} \Lambda^{r-1} + \varepsilon_{Qd} \\
&- \frac{1}{2^r} k_5^r p_L^r \Lambda^T \Lambda^{2r-1} - C^r + \frac{1}{2} k_5^r \Lambda^T \Lambda^{2r-1} + C^r \\
&\le - \sum_{i=1}^{3} S_{1i} e_{1i}^{2r} - k_2 \sum_{i=1}^{3} S_{3i} e_{2i}^{2r} - k_5^r \Lambda^T e_2^T e_2^{r-1} \Lambda^{r-1} \\
&- \frac{1}{2^r} k_5^r p_L^r \Lambda^T \Lambda^{2r-1} - C^r - \Pi_1 + \Pi_2
\end{aligned} \tag{54}
$$

where $S_3 = diag\{S_{31}, S_{32}, S_{32}\} = k_2 S_2 - \frac{1}{2} I_{3\times 3}$, $\Pi_1 = \tau_1 \Upsilon^T \Upsilon$, $\Pi_2 = \frac{1}{2} \tilde{d}_1^T \tilde{d}_1 + \varepsilon_{dm} + k_5^r \left(\frac{1}{2} + \frac{p_L^r}{2^r} \right) \Lambda^T \Lambda^{2r-1} + C^r + \varepsilon_{Qd}$.

By utilizing Lemma 2, the following results are obtained:

$$
- \sum_{i=1}^{3} S_{1i} e_{1i}^{2r} \le -\overline{S}_{1m} \left(\frac{1}{2} \sum_{i=1}^{3} S_{1i} e_{1i}^{2} \right)^r \tag{55}
$$

$$
-k_2 \sum_{i=1}^{3} S_{3i} e_{2i}^{2r} \le -S_{3m} \sum_{i=1}^{3} e_{2i}^{2r} \le -\overline{S}_{3m} \left(\frac{k_2}{2} \sum_{i=1}^{3} e_{2i}^{2} \right)^r \tag{56}
$$

where $S_{3m} = \lambda_{min}(S_3)$, $\bar{S}_{3m} = S_{3m} 2^r k_2^{-r}$, $\bar{S}_{1m} = 2^r$.

According to Lemma 3, when taking $\sigma_1 = \Pi_1$, $\sigma_2 = 1$, $b_1 = r$, $b_2 = 1 - r$ and $b_3 = \frac{1}{r}$, the following is obtained

$$
-\Pi_1 \le -{\Pi_1}^r + (1 - r) \frac{1}{r}^{\frac{r}{1-r}} \tag{57}
$$

Invoking equations (55), (56), and (57), equation (54) can be rewritten in

the form

$$
\begin{aligned}
\dot{V}_4(t) \le & -\bar{S}_{1m}\left(\frac{1}{2}\sum_{i=1}^{3} S_{1i}e_{1i}^2\right)^r - \bar{S}_{3m}\left(\frac{k_2}{2}\sum_{i=1}^{3} e_{2i}^2\right)^r - k_5^r\Lambda^T e_2^T e_2^{r-1}\Lambda^{r-1} \\
& - \frac{1}{2^r}k_5^r p_L^r \Lambda^T \Lambda^{2r-1} - C^r - \Pi_1^r + (1-r)\left(\frac{1}{r}\right)^{\frac{r}{1-r}} + \Pi_2 \\
\le & -\Pi_3 V_4^r(t) + \Pi_4
\end{aligned} \tag{58}
$$

where $\Pi_3 = \min\{\bar{S}_{1m}, \bar{S}_{3m}, 1\}$, and $\Pi_4 = (1-r)\frac{1}{r}^{\frac{r}{1-r}} + \Pi_2$.

The system exhibits finite-time convergence as established by Lemma 1. Furthermore, the upper bound of the convergence time is given by $T_{st} \le \frac{1}{(1-r)\vartheta\delta_1}[V_4^{1-r}(x(0)) - (\frac{\delta_2}{(1-\vartheta)\delta_1})^{\frac{1-r}{r}}]$. The proof is completed.

Remark 2. In this section, the energy function method is employed to suppress the swing angle of the payload. In the aforementioned control design process, the energy function is used to construct the Lyapunov function. Through rigorous derivation and proof, it can be concluded that the Lyapunov function is monotonically decreasing, which means the energy function is also monotonically decreasing. Considered the definition of energy function, it is noted that it consists of the kinetic energy and potential energy of the payload. Hence, as the energy function decreases continuously, both the kinetic energy and potential energy gradually diminish, and the swing angles will eventually converge to an equilibrium state. This is precisely the principle by which the energy function method suppresses the swing angle of the payload.

Remark 3. In this section, the compensation function observer method is adopted to estimate the unknown disturbance. Compared with the traditional ESO (its specific design form is given in the simulation), the form of the error system (16)-(18) is a standard cascade system and it is more simple for the control design. More importantly, both λ_1 and λ_2 are utilized to observe the unknown disturbance. In other words, different from only using λ_1 in the traditional ESO-based appraoch, all system information is fully usage in (15). Hence, the developed compensation function observer has higher observation accuracy.

4 Simulation Results

This section validates the control performance of the QSLS under the proposed control method. The physical parameters of the QSLS are set as follows:

$$m_Q = 0.468 \text{ kg}, m_L = 0.068 \text{ kg}, p_L = 0.9 \text{ m}, g = 9.81 \text{ m/s}^2$$

The initial swing angles, position and attitude angles of the QSLS are assumed as: $x_{Q_0} = y_{Q_0} = z_{Q_0} = 0$ m, and $\alpha_0 = \beta_0 = 0$ rad. The expected position of the quadrotor is selected as $\xi_{Qd} = [0.9\cos(0.2t), 1 + \sin(0.2t), 2.5]^T$m. The external

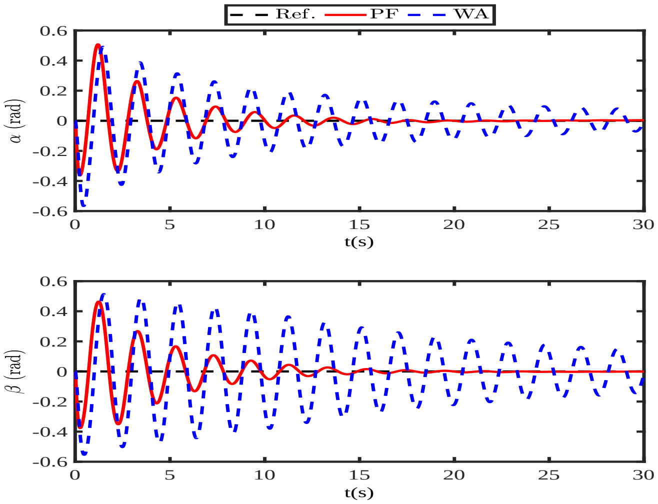

Fig 3. Tracking results of swing angles

disturbances d are supposed as

$$d = \begin{bmatrix} 1.6\sin(0.3t) \\ 2.1\sin(0.4t) \\ 1.2\sin(0.02t) \end{bmatrix}$$

Meanwhile, the selection of user-specified parameters is $\eta_1 = 0.1$, $\eta_2 = 2$, $N_1 = \text{diag}\{2, 2, 2, 2, 2\}$, $N_2 = \text{diag}\{20, 20, 20, 20, 20\}$, $S_Q = 0.011$, $S_L = 26$, $r = 0.9$, $S_1 = \text{diag}\{15, 12, 10\}$, $S_2 = \text{diag}\{2.1, 1.85, 4.5\}$.

The PID controller is designed as

$$\begin{aligned} F(t) = &-s_{\text{p1}}e_1(t) - s_{\text{i1}}\int e_1(\tau)d\tau - s_{\text{d1}}\dot{e}_1(t) \\ &+s_{\text{p2}}e_\Omega(t) + s_{\text{i2}}\int e_\Omega(\tau)d\tau + s_{\text{d2}}\dot{e}_\Omega(t) \end{aligned}$$

where $e_\Omega = [\alpha - \alpha_d, \beta - \beta_d]^T$, s_{p1}, s_{i1}, s_{d1}, s_{p2}, s_{i2} and s_{d2} are parameter matrices.

The simulation results are presented in Figs. 3-10. Fig. 3 shows the comparison results of whether the swing angles are suppressed, where the black lines (Ref.) represent the expected trajectories, the red lines (PF) represent the swing angles under the energy function method, and the blue lines (WA) denote the swing angles without control. From Fig. 3, it can be seen that if the swing angles are not controlled, it will continue to swing left and right, which is detrimental to flight safety. However, under the proposed energy function-based method, the swing angles can converge to the neighborhood of zero in a short time. Meanwhile, in order to reveal the advantage of the energy function method,

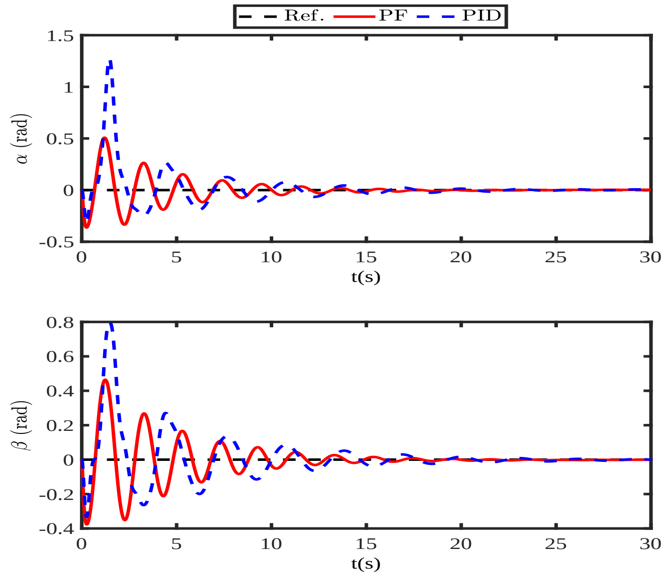

Fig 4. Comparison anti-swing results under the proposed energy function method and PID method

the comparison anti-swing results are provided in Fig. 4, where the blue lines (PID) denote the swing angles under the PID controller. From Fig. 4, it is observed that the proposed energy function-based method enables the swing angles to have a smaller overshoot and a faster convergence speed.

Fig. 5 illustrates the impact of external unknown disturbances on the tracking performance of the QSLS, where the black lines (Ref.) represent the reference trajectories, the red lines (PF) refer to the actual trajectory under the proposed control scheme, and the blue lines (WDO) depict the tracking results with the disturbance being not suppressed. Fig. 5 clearly demonstrates that external disturbances significantly degrade the safety performance of the QSLS. If the unknown disturbances cannot be addressed in a timely manner, the stability of the QSLS will be implicated. In addition, to present the control effect more clearly, the 3D tracking results of position loop are provided in Fig. 6.

To further indicate the superiority of the developed compensation function observer, the ESO method and the adaptive disturbance observer (ADO) method are compared. The ESO is designed as

$$\begin{cases} \lambda_1 = z_1 - \hat{z}_1 \\ \dot{\hat{z}}_1 = \hat{z}_2 + N_{s1}\lambda_1 \\ \dot{\hat{z}}_2 = R^{-1}(z_1)(-J(z_1, z_2)z_2 - M(z_1) + H) + \hat{z}_3 + N_{s2}\lambda_1 \\ \dot{\hat{z}}_3 = N_{s3}\lambda_1 \end{cases}$$

where N_{s1}, N_{s2} and N_{s3} are the designed parameters.

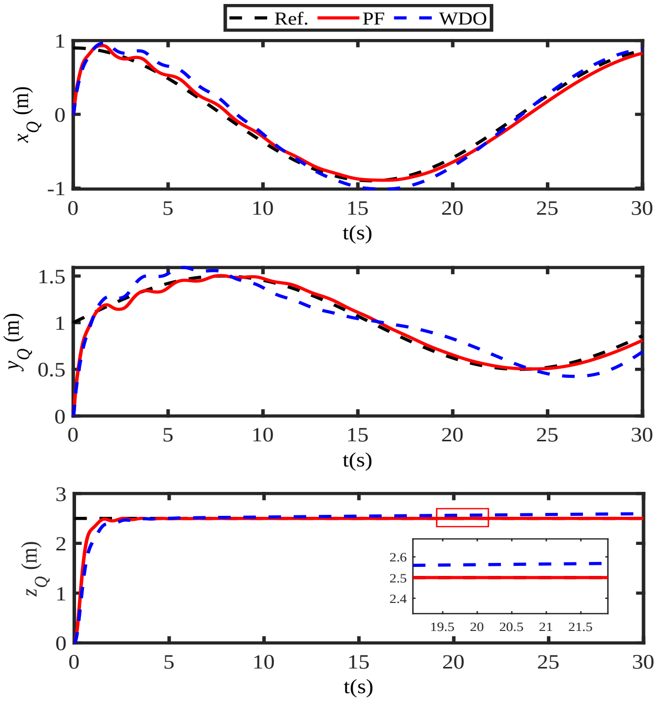

Fig 5. Tracking results of position loop

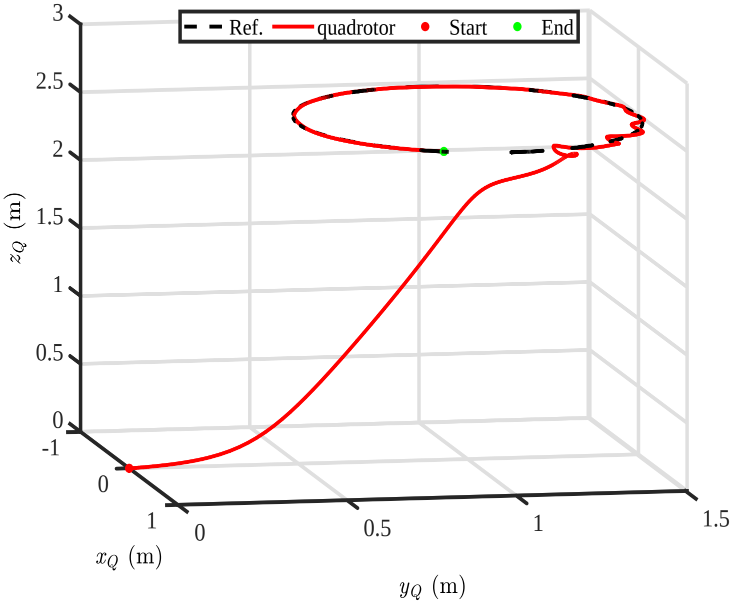

Fig 6. Three-dimensional tracking results of position loop

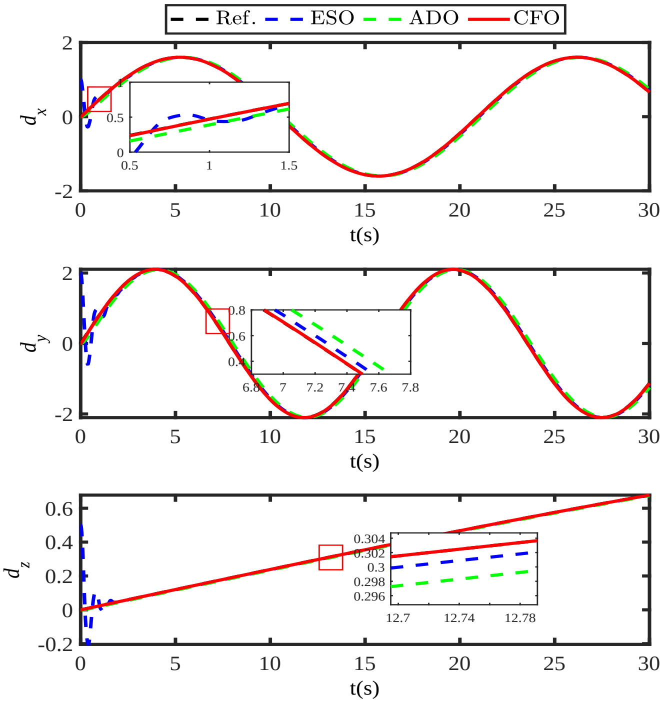

Fig 7. Comparison disturbance estimation results under different methods

The ADO is designed as

$$\begin{cases} \dot{\hat{z}}_1 = \hat{z}_2 \\ \dot{\hat{z}}_2 = R^{-1}(z_1)(-J(z_1, z_2)z_2 - M(z_1) + H) + \hat{z}_3 \\ \dot{\hat{z}}_3 = K\left(z_2 - \hat{z}_2\right) \end{cases}$$

where K is the designed parameter.

Fig. 7 gives the disturbance estimation comparison results of the ESO approach, the ADO approach and the proposed compensation function observer approach. Fig. 8 shows the estimation errors under these different methods. In Fig. 7 and Fig. 8, the black lines (Ref.) represent the actual disturbances, the blue lines (ESO) indicate the disturbance estimations under the ESO method, the green lines (ADO) denote the disturbance estimations under the ADO method, and the red lines (CFO) represent the disturbance estimations under the presented compensation function observer method. The comparative results in Fig. 7 and Fig. 8 clearly demonstrate that the proposed compensation function observer method can ensure the QSLS have higher tracking accuracy and smaller tracking error. Moreover, to achieve satisfactory tracking result, the gain values of ESO are selected as $N_{s1} = \text{diag}\{250, 250, 250, 250, 250\}$, $N_{s2} = \text{diag}\{1380, 1380, 1380, 1380, 1380\}$,

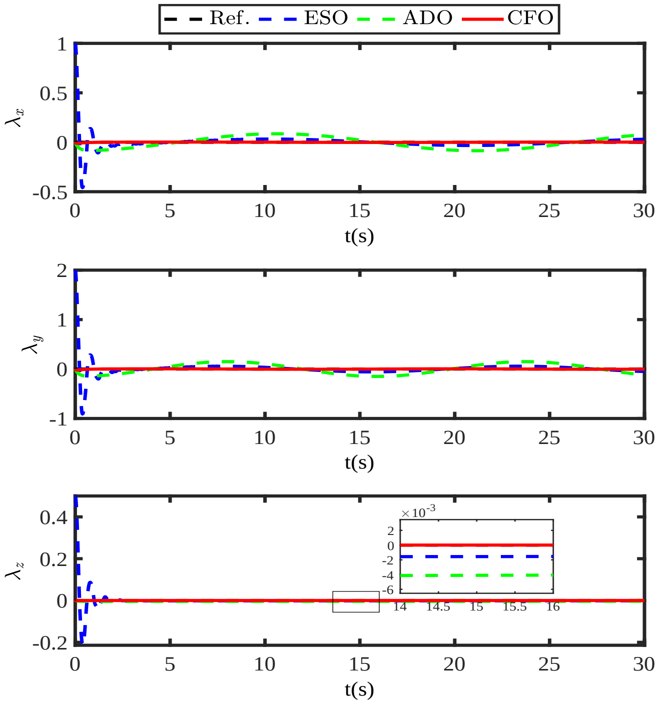

Fig 8. Comparison disturbance estimation errors under under different methods

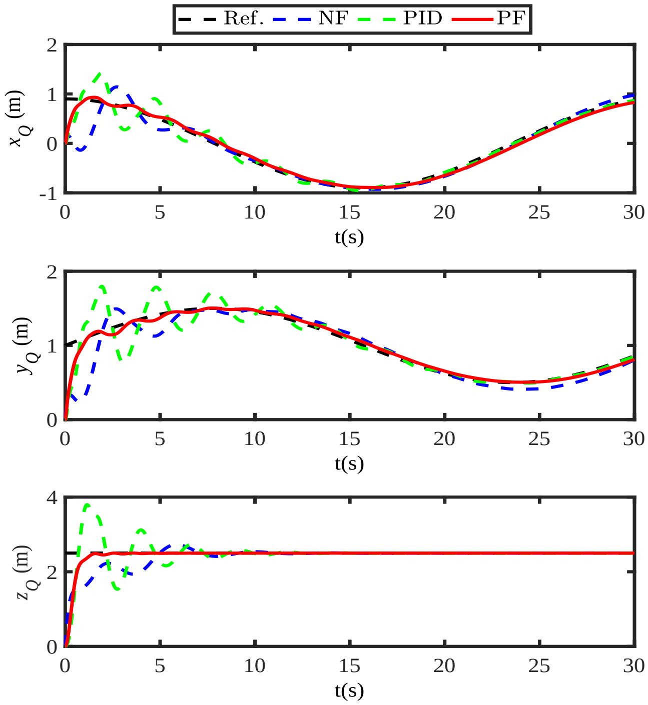

Fig 9. Comparison tracking results under the different control methods

$N_{s3} = \text{diag}\{9000, 9000, 9000, 9000, 9000\}$. Obviously, the gain value of ESO is much larger than that of the compensation function observer. This also leads to the issue of initial peaking in the traditional ESO method.

Subsequently, Fig. 9 compares the overall tracking performance of the QSLS under the proposed finite-time anti-swing control method, the conventional PID controller and traditional backstepping control method. In Fig. 9, the black lines (Ref.) represent the reference trajectories, the bule lines (NF) represent the tracking errors under the backstepping control method, the green lines (PID) represent the tracking errors under the PID controller, and the red lines (PF) denote the tracking errors under the proposed controller. From Fig. 9, it is clear that the proposed finite-time control strategy has a faster convergence speed, which implies that the proposed control strategy can ensure the system has good transient performance. Finally, the curves of the designed finite-time anti-swing controller is illustrated in Fig. 10. To sum up, the simulation results confirm that the designed controller significantly suppresses load swing and effectively ensures stable flight of the QSLS.

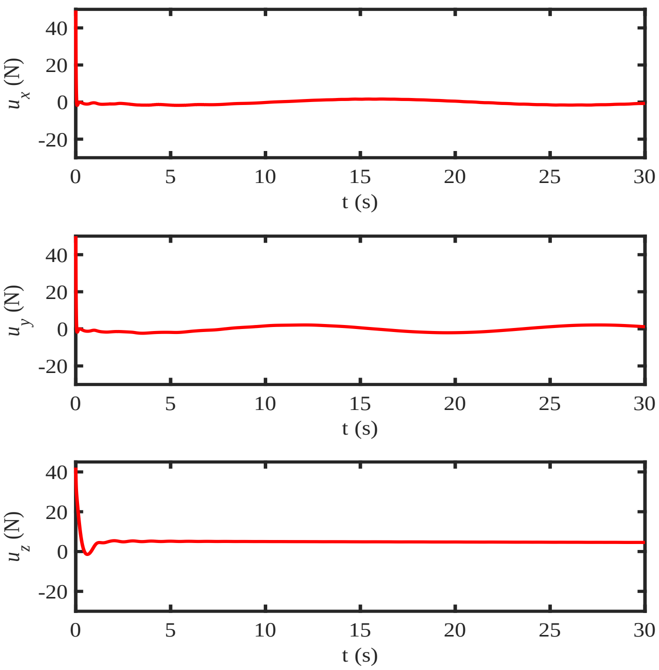

Fig 10. The curves of the finite-time anti-swing controller

5 Conclusion

The finite-time anti-swing control problem has been investigated for the QSLS suffering from external disturbances. Firstly, based on comprehensive force analysis and kinematic constraints, the position loop nonlinear dynamics of the QSLS has been developed. Then, the compensation function observer has been designed to handle the unknown disturbances during the flight process. Finally, the energy function method has been combined with the finite-time theory to develop the robust anti-swing controller for the QSLS, ensuring the tracking performance and fast convergence of the whole system. Simulation results have demonstrated the feasibility and superior performance of the proposed method.

Acknowledgement

The authors thank the colleagues for their constructive suggestions and research assistance throughout this study.

Author Contributions

Conceptualization: Xiaoning Yu.
Methodology: Kun Yan, Jiayong Fang.
Investigation: Peng Zhang, Bo Cheng.
Writing-original draft preparation: Kun Yan, Liaozhang Li.
Writing-review and editing: Xiaoning Yu, Liaozhang Li.
Supervision: Kun Yan.
Project administration: Bo Cheng.
Funding acquisition: Kun Yan, Jiayong Fang, Peng Zhang.

Fundings

This work was supported in part by the National Natural Science Foundation of China under Grant 62303367 (K. Y.), the National Key Laboratory of Unmanned Aerial Vehicle Technology in NPU under Grant WR202412 (J. F., P. Z.), the Shaanxi Provincial Education Department Service Local Special Plan Project under Grant 24JC038 (K. Y.), the Science and Technology Plan Project of Beilin District under Grant GX2413 (K. Y.), the Science and Technology Plan Project of Weiyang District under Grant 202426 (K. Y.), and the 2023 Youth Innovation Team of Shaanxi Universities (K. Y.). The funders had no role in the study design, data collection and analysis, decision to publish, or preparation of the manuscript.

Data Availability Statement

All data generated or analyzed are included in this study.

Competing Interests

The authors declare no conflict of interest.

References

1. Bacelar T, Madeiras J, Melicio R, Cardeira C, Oliveira P. On-Board Implementation and Experimental Validation of Collaborative Transportation of Loads With Multiple UAVs. Aerospace Science and Technology. 2020;107. doi:10.1016/j.ast.2020.106284.

2. Zhang D, Han HZ, Yang YX, Hu YB. Modeling and Anti-Swing Control of Quadrotor-Suspended System With Variable Cable Length at Unequal Speed. Transactions of the Institute of Measurement and Control. 2024;46(6):1177–1189. doi:10.1177/01423312231187446.

3. Baraean A, Hamanah WM, Bawazir A, Quama MM, El Ferik S, Baraean S, et al. Optimal Nonlinear Backstepping Controller Design of a Quadrotor-Slung Load System Using Particle Swarm Optimization. Alexandria Engineering Journal. 2023;68:551–560. doi:10.1016/j.aej.2023.01.024.

4. Liang X, Zhang Z, Yu H, Wang Y, Fang YC, Han JD. Antiswing Control for Aerial Transportation of the Suspended Cargo by Dual Quadrotor UAVs. IEEE-ASME Transactions on Mechatronics. 2022;27(6):5159–5172. doi:10.1109/tmech.2022.3174144.

5. Qian LH, Liu HHT. Path-Following Control of a Quadrotor UAV With a Cable-Suspended Payload Under Wind Disturbances. IEEE Transactions on Industrial Electronics. 2020;67(3):2021–2029. doi:10.1109/tie.2019.2905811.

6. Xian B, Wang SZ, Yang S. An Online Trajectory Planning Approach for a Quadrotor UAV With a Slung Payload. IEEE Transactions on Industrial Electronics. 2019;67(8):6669–6678. doi:10.1109/tie.2019.2938493.

7. Yu H, Liang X, Han JD, Fang YC. Adaptive Trajectory Tracking Control for the Quadrotor Aerial Transportation System Landing a Payload Onto the Mobile Platform. IEEE Transactions on Industrial Informatics. 2024;20(1):23–37. doi:10.1109/tii.2023.3256374.

8. Li XX, Zhang JL, Han JD. Trajectory Planning of Load Transportation With Multi-Quadrotors Based on Reinforcement Learning Algorithm. Aerospace Science and Technology. 2021;116. doi:10.1016/j.ast.2021.106887.

9. Guerrero-Sanchez ME, Lozano R, Castillo P, Hernandez-Gonzalez O, Garcia-Beltran CD, Valencia-Palomo G. Nonlinear Control Strategies for a UAV Carrying a Load With Swing Attenuation. Applied Mathematical Modelling. 2021;91:709–722. doi:10.1016/j.apm.2020.09.027.

10. Sreenath K, Lee T, Kumar V. Geometric Control and Differential Flatness of a Quadrotor UAV With a Cable-Suspended Load. In: Proceedings of the IEEE Conference on Decision and Control; 2013. p. 2269–2274.

11. Yu G, Xie W, Cabecinhas D, Cunha R, Silvestre C. Adaptive Control with Unknown Mass Estimation for a Quadrotor-Slung-Load System. ISA Transactions. 2023;133:412–423. doi:10.1016/j.isatra.2022.06.036.

12. Yan B, Lin H, Cai CX, Shi P. An Enhanced Coupling Nonlinear Control for Quadrotor with Suspended Double-Pendulum Payload. International Journal of Robust and Nonlinear Control. 2024;34(14):9676–9696. doi:10.1002/rnc.7483.

13. Zhu BY, Wang DZ. Nonlinear Adaptive Control Design for Quadrotor UAV Transportation System. Drones. 2024;8(9). doi:10.3390/drones8090420.

14. Luo YH, Yu H, Zhang HG, Zhou Y. A Novel Newton-Euler Method-Based Nonlinear Anti-Swing Control for a Quadrotor UAV Carrying a Slung Load. IEEE Transactions on Systems, Man, and Cybernetics: Systems. 2024;54(4):2266–2275. doi:10.1109/tsmc.2023.3342027.

15. Liang X, Yu H, Zhang Z, Liu HW, Fang YC, Han JD. Unmanned Aerial Transportation System with Flexible Connection Between the Quadrotor and the Payload: Modeling, Controller Design, and Experimental Validation. IEEE Transactions on Industrial Electronics. 2022;70(2):1870–1882. doi:10.1109/tie.2022.3163526.

16. Yang S, Xian B. Energy-Based Nonlinear Adaptive Control Design for the Quadrotor UAV System With a Suspended Payload. IEEE Transactions on Industrial Electronics. 2020;67(3):2054–2064. doi:10.1109/tie.2019.2902834.

17. Han JQ. From PID to Active Disturbance Rejection Control. IEEE Transactions on Industrial Electronics. 2009;56(3):900–906. doi:10.1109/tie.2008.2011621.

18. Zhao ZL, Guo BZ. On Active Disturbance Rejection Control for Nonlinear Systems Using Time-Varying Gain. European Journal of Control. 2015;23:62–70. doi:10.1016/j.ejcon.2015.02.002.

19. Cui RX, Chen LP, Yang CG, Chen M. Extended State Observer-Based Integral Sliding Mode Control for an Underwater Robot With Unknown Disturbances and Uncertain Nonlinearities. IEEE Transactions on Industrial Electronics. 2017;64(8):6785–6795. doi:10.1109/tie.2017.2694410.

20. Yan K, Chen HT, Chen CB, Gao S, Sun JL. Time-Varying Gain Extended State Observer-Based Adaptive Optimal Control for Disturbed Unmanned Helicopter. ISA Transactions. 2024;148:1–11. doi:10.1016/j.isatra.2024.02.028.

21. Lu XJ, Li ZH, Xu J. Design and Control of a Hand-Launched Fixed-Wing Unmanned Aerial Vehicle. IEEE Transactions on Industrial Informatics. 2023;19(3):3006–3016. doi:10.1109/tii.2022.3167840.

22. Lotufo MA, Colangelo L, Novara C. Control Design for UAV Quadrotors via Embedded Model Control. IEEE Transactions on Control Systems Technology. 2020;28(5):1741–1756. doi:10.1109/tcst.2019.2918750.

23. Xu LX, Ma HJ, Guo D, Xie AH, Song DL. Backstepping Sliding-Mode and Cascade Active Disturbance Rejection Control for a Quadrotor UAV. IEEE-ASME Transactions on Mechatronics. 2020;25(6):2743–2753. doi:10.1109/tmech.2020.2990582.

24. Yan K, Ren HP. Fault Identification and Fault-Tolerant Control for Unmanned Autonomous Helicopter With Global Neural Finite-Time Convergence. Neurocomputing. 2021;459:165–175. doi:10.1016/j.neucom.2021.06.081.

25. Chen B, Lin C. Finite-Time Stabilization-Based Adaptive Fuzzy Control Design. IEEE Transactions on Fuzzy Systems. 2021;29(8):2438–2443. doi:10.1109/tfuzz.2020.2991153.

26. Wang HQ, Liu PXP, Zhao XD, Liu XP. Adaptive Fuzzy Finite-Time Control of Nonlinear Systems With Actuator Faults. IEEE Transactions on Cybernetics. 2020;50(5):1786–1797. doi:10.1109/tcyb.2019.2902868.

27. Tian BL, Cui J, Lu HC, Zuo ZY, Zong Q. Adaptive Finite-Time Attitude Tracking of Quadrotors With Experiments and Comparisons. IEEE Transactions on Industrial Electronics. 2019;66(12):9428–9438. doi:10.1109/tie.2019.2892698.

28. Chen YJ, Liang JC, Wu YN, Miao ZQ, Zhang H, Wang YN. Adaptive Sliding-Mode Disturbance Observer-Based Finite-Time Control for Unmanned Aerial Manipulator With Prescribed Performance. IEEE Transactions on Cybernetics. 2022;53(5):3263–3276. doi:10.1109/tcyb.2022.3168030.

29. Yan K, Chen M, Wu QX, Lu K. Robust Attitude Fault-Tolerant Control for Unmanned Autonomous Helicopter With Flapping Dynamics and Actuator Faults. Transactions of the Institute of Measurement and Control. 2019;41(5):1266–1277. doi:10.1177/0142331218775477.

30. Liu W, Chen M, Shi P. Fixed-Time Disturbance Observer-Based Control for Quadcopter Suspension Transportation System. IEEE Transactions on Circuits and Systems I: Regular Papers. 2022;69(11):4632–4642. doi:10.1109/tcsi.2022.3193878.

31. Liang X, Lin H, Zhang P, Wu SZ, Sun N, Fang YC. A Nonlinear Control Approach for Aerial Transportation Systems With Improved Antiswing

and Positioning Performance. IEEE Transactions on Automation Science and Engineering. 2020;18(4):2104–2114. doi:10.1109/tase.2020.3035178.
