# Supplementary figures and images for: Robust finite-time anti-swing control for quadrotor slung-load system based on compensation function observer
Source: PLoS One. 2026 Apr 24;21(4):e0331662. doi: 10.1371/journal.pone.0331662 (PMC13108781; doi:10.1371/journal.pone.0331662)

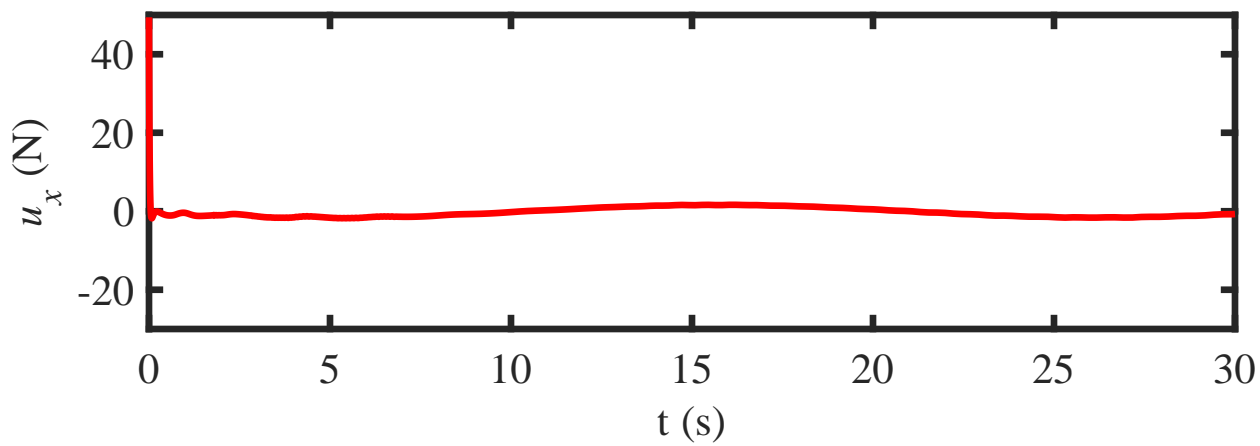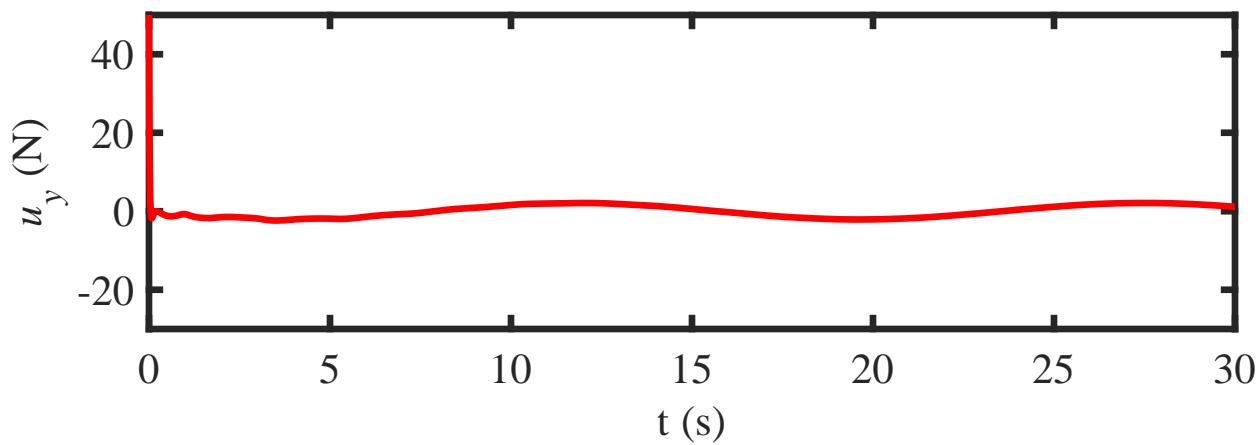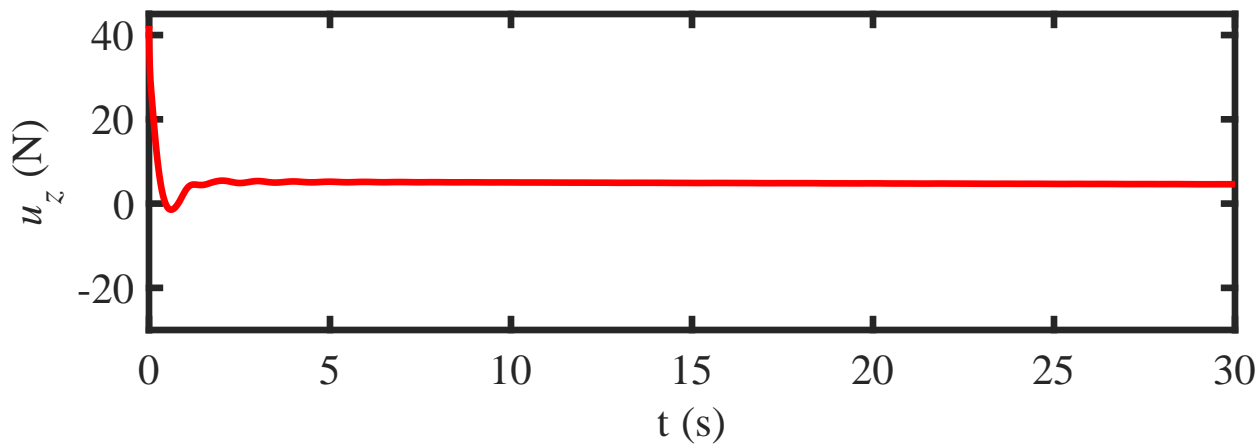

Supplement: S1 File — (ZIP) [file pone.0331662.s001.zip › LaTeX source file (with figures)-bib/Fig10-eps-converted-to.pdf]

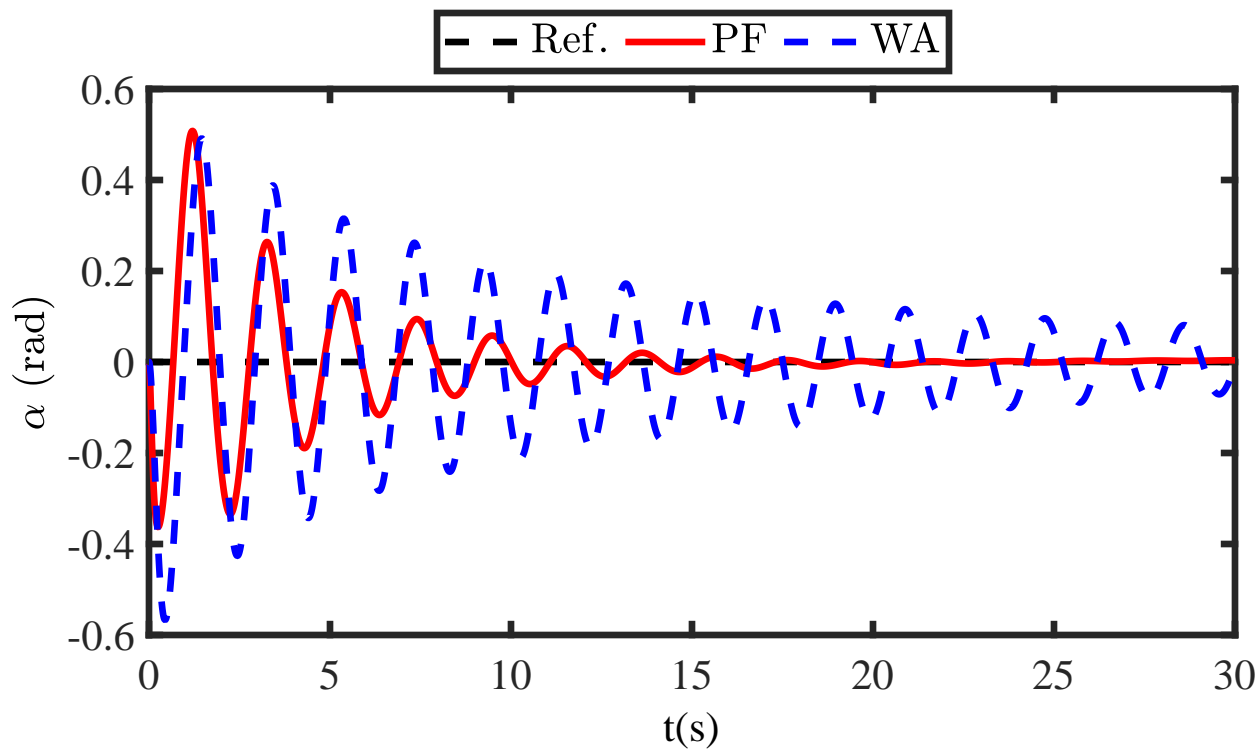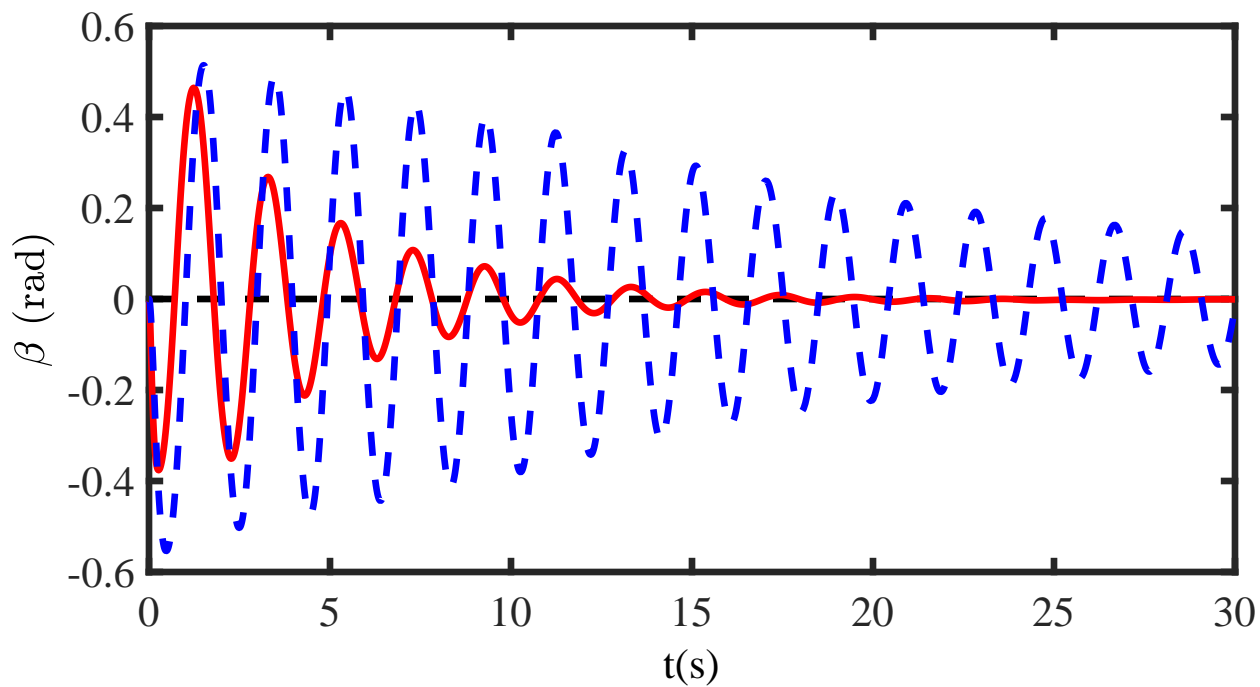

Supplement: S1 File — (ZIP) [file pone.0331662.s001.zip › LaTeX source file (with figures)-bib/Fig3-eps-converted-to.pdf]

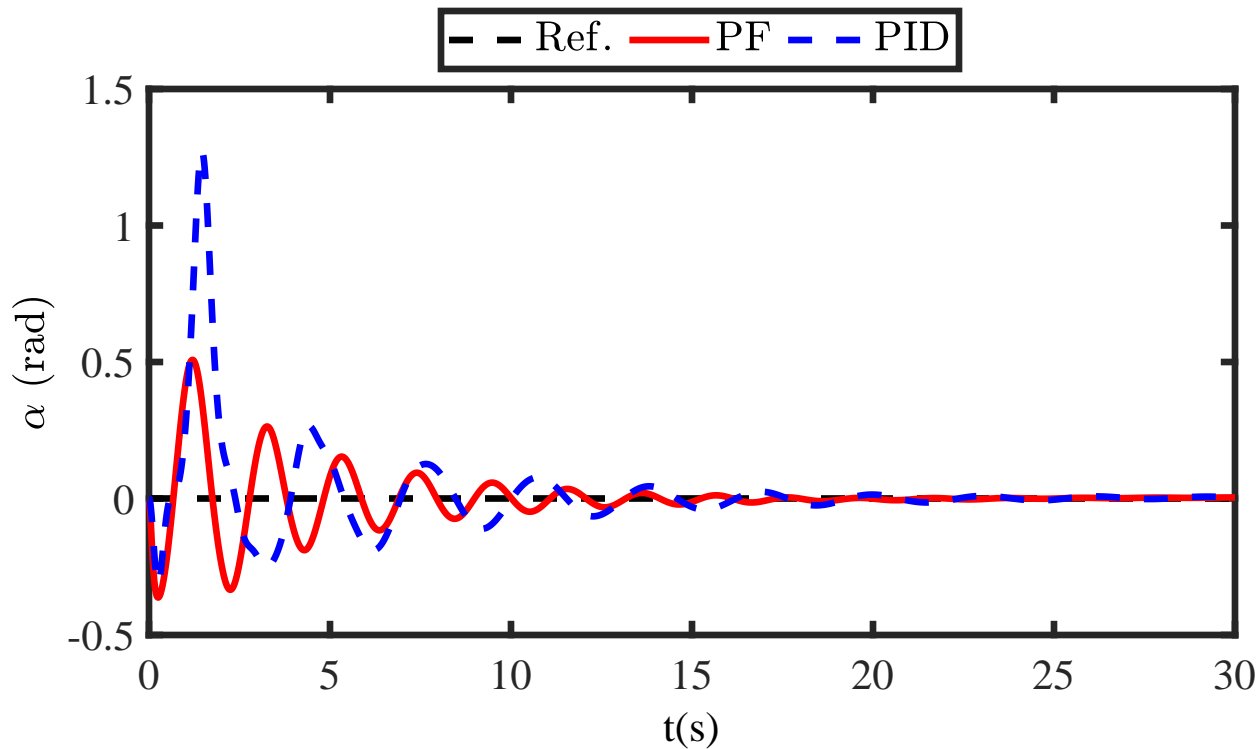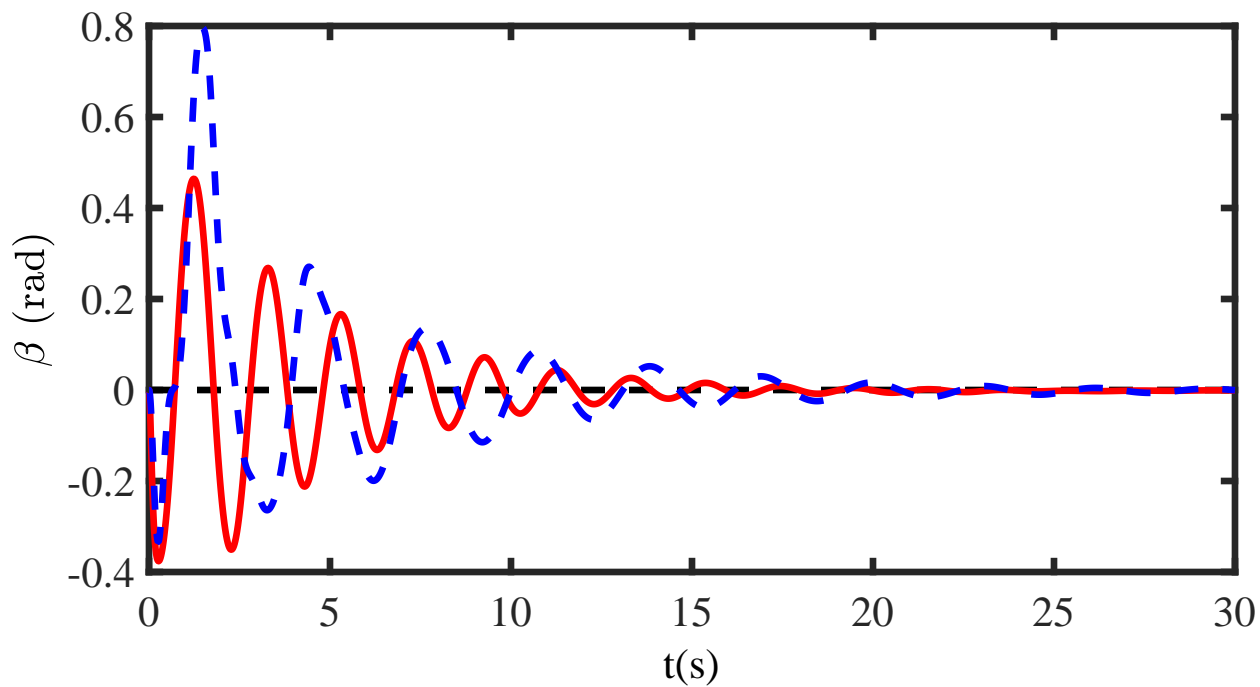

Supplement: S1 File — (ZIP) [file pone.0331662.s001.zip › LaTeX source file (with figures)-bib/Fig4-eps-converted-to.pdf]

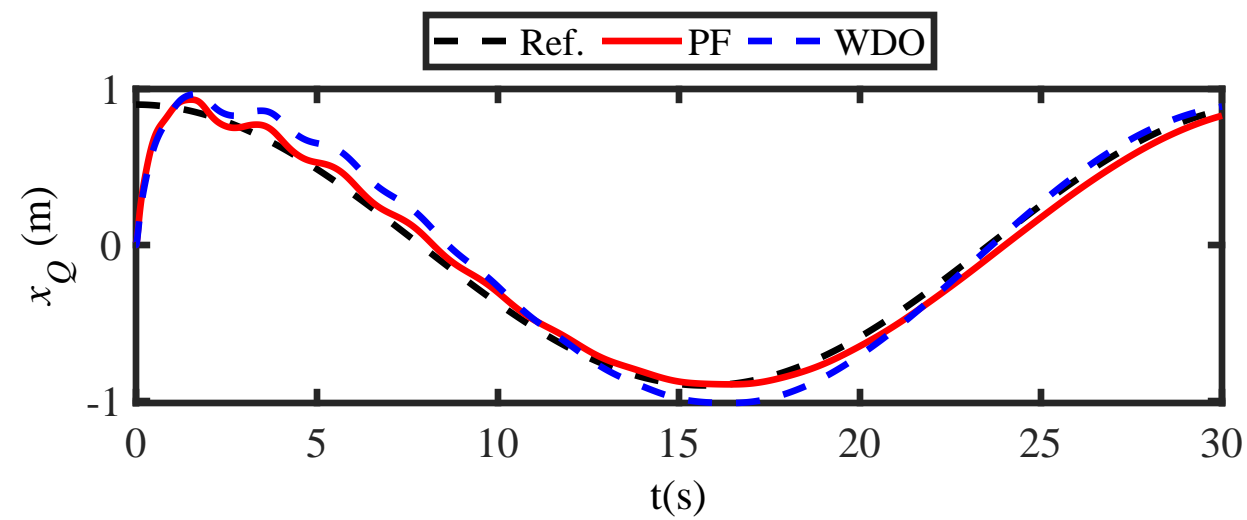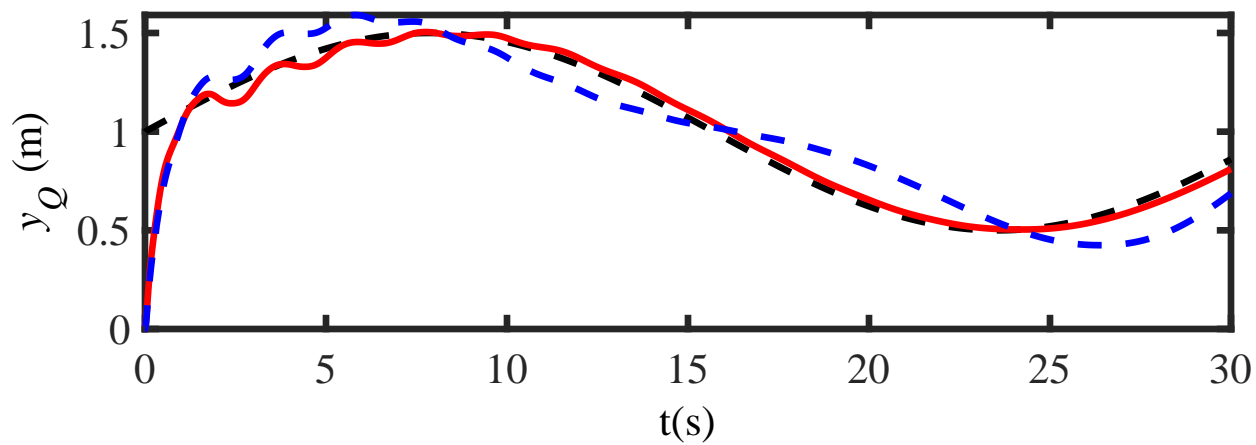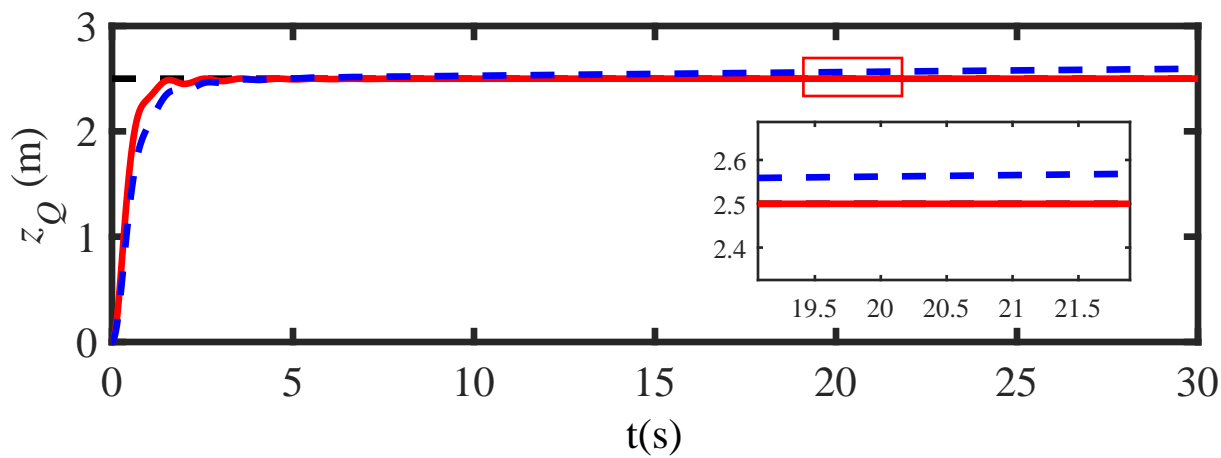

Supplement: S1 File — (ZIP) [file pone.0331662.s001.zip › LaTeX source file (with figures)-bib/Fig5-eps-converted-to.pdf]

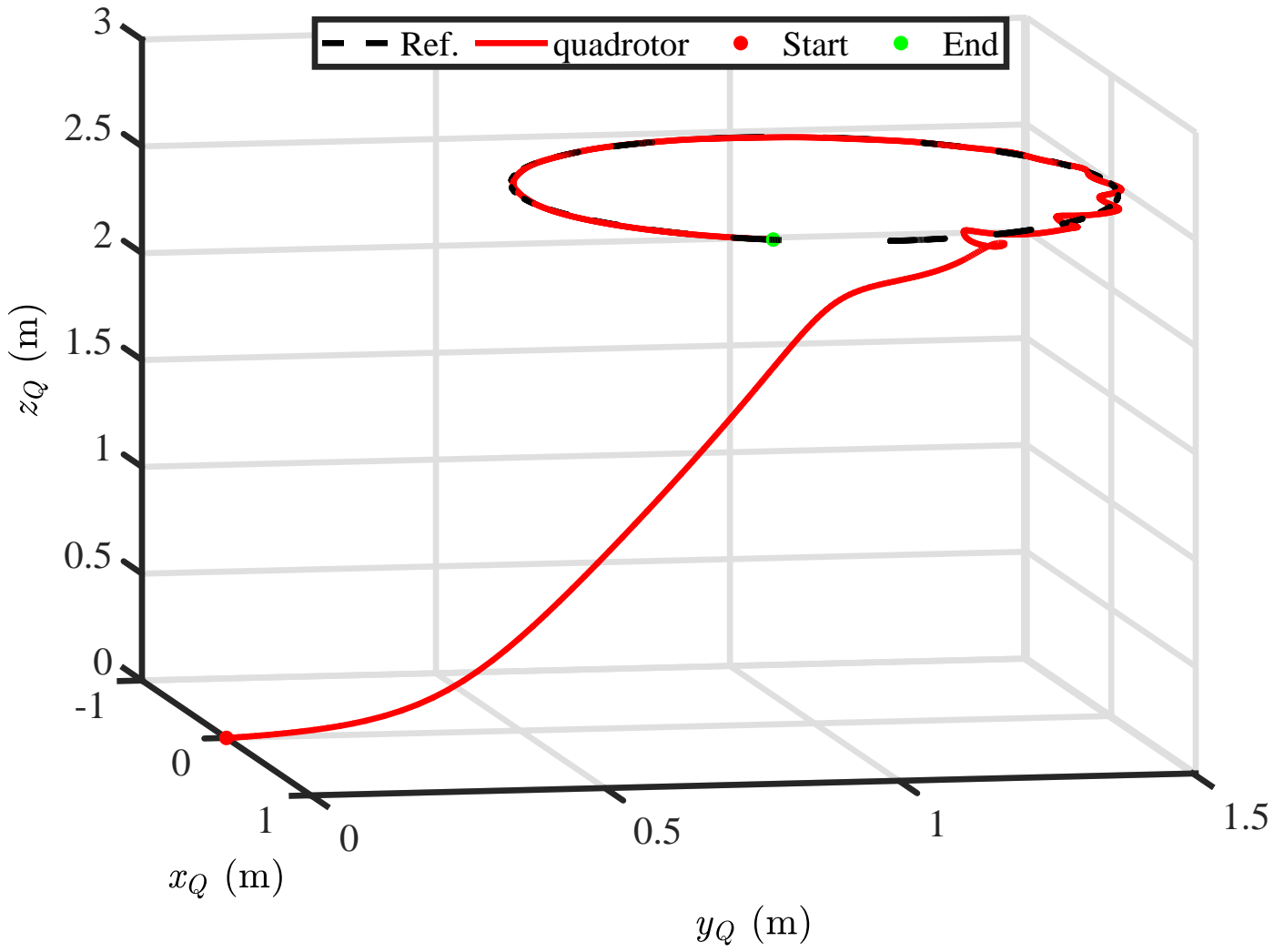

Supplement: S1 File — (ZIP) [file pone.0331662.s001.zip › LaTeX source file (with figures)-bib/Fig6-eps-converted-to.pdf]

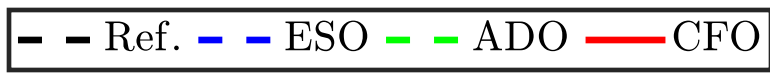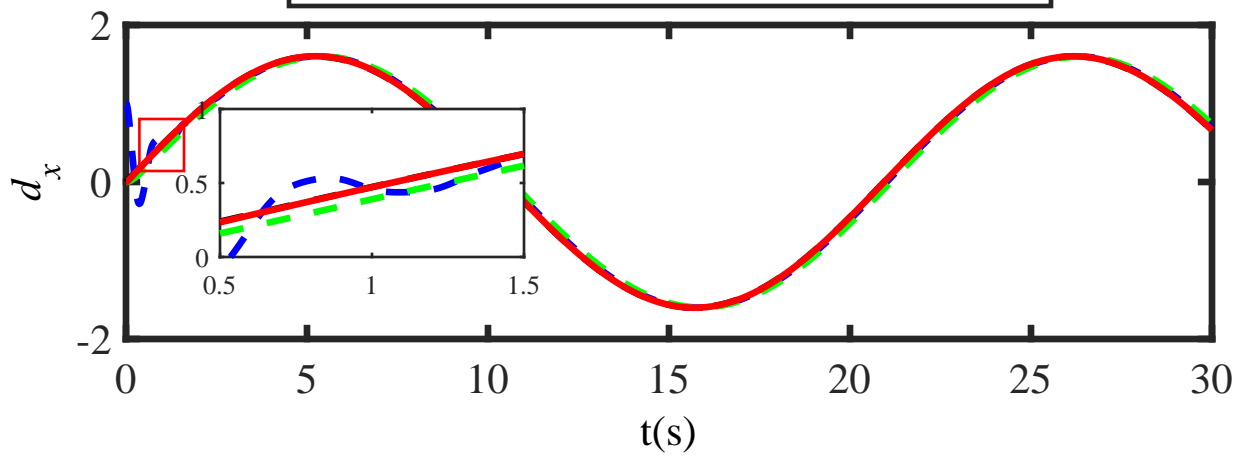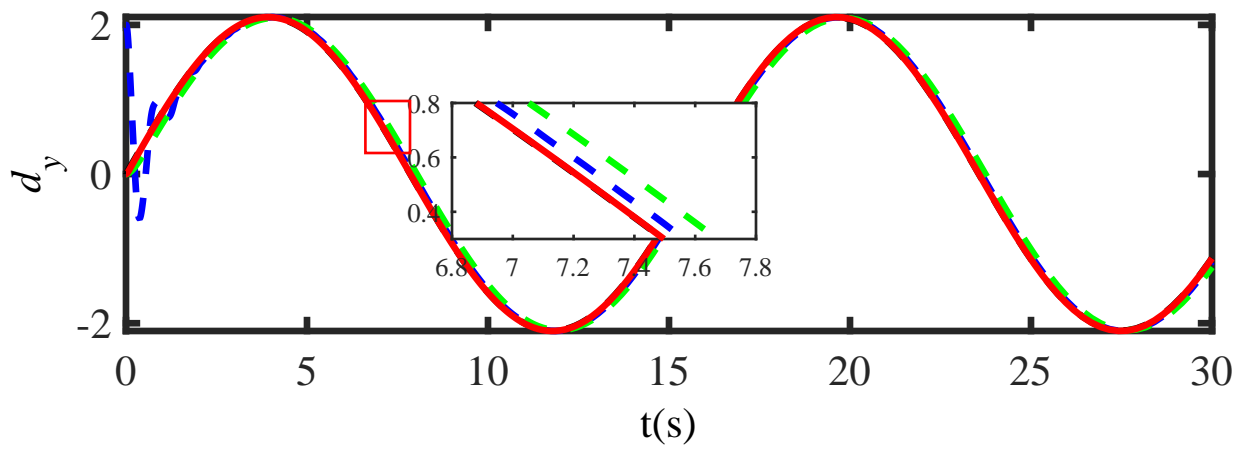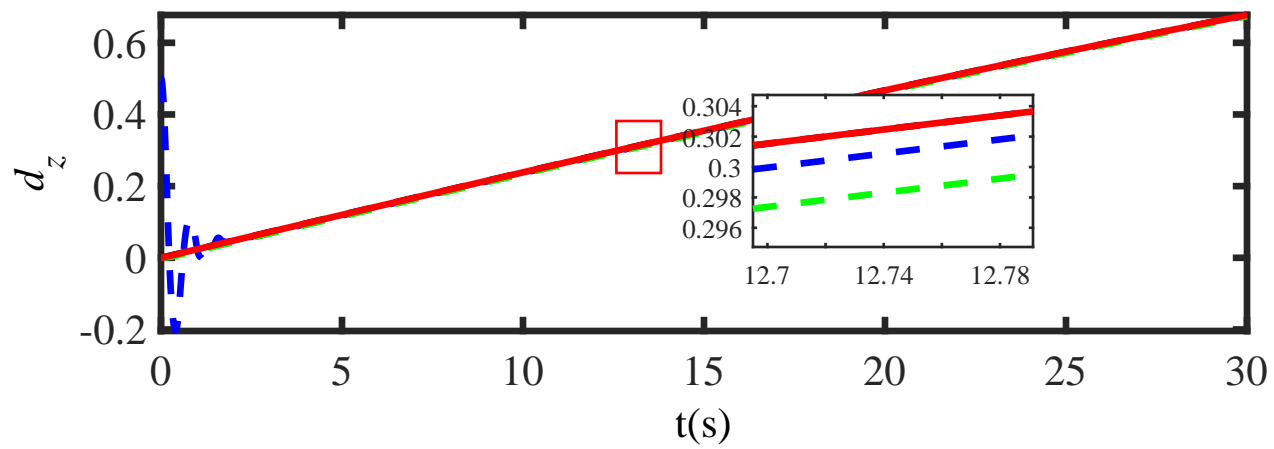

Supplement: S1 File — (ZIP) [file pone.0331662.s001.zip › LaTeX source file (with figures)-bib/Fig7-eps-converted-to.pdf]

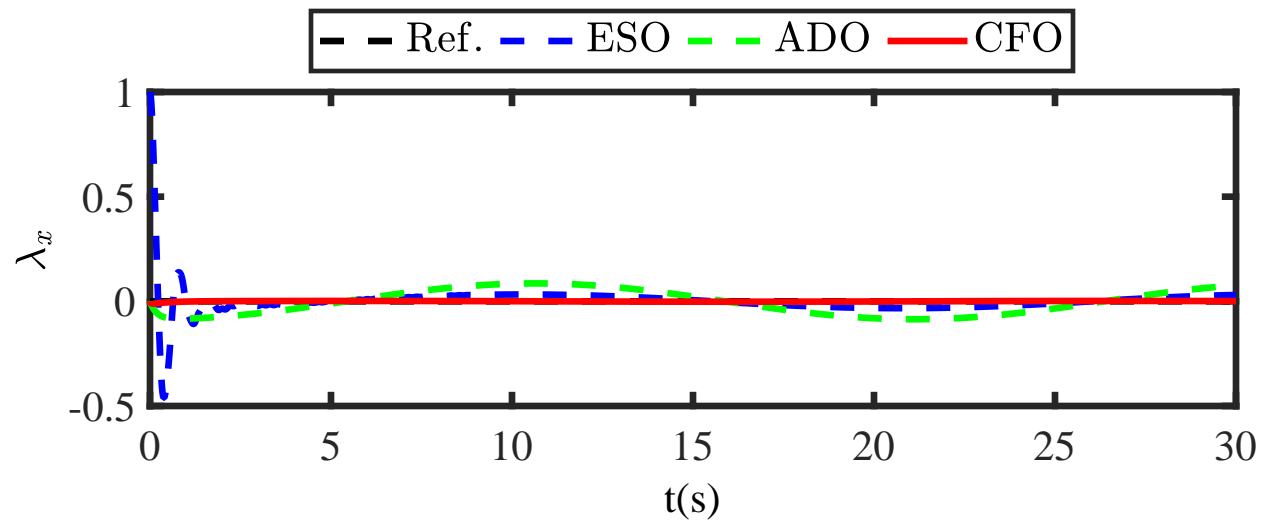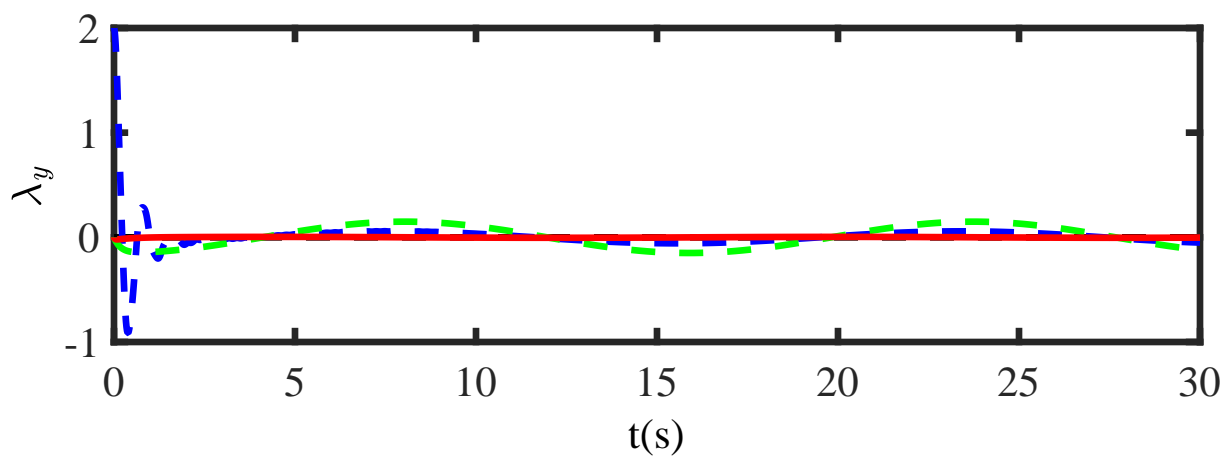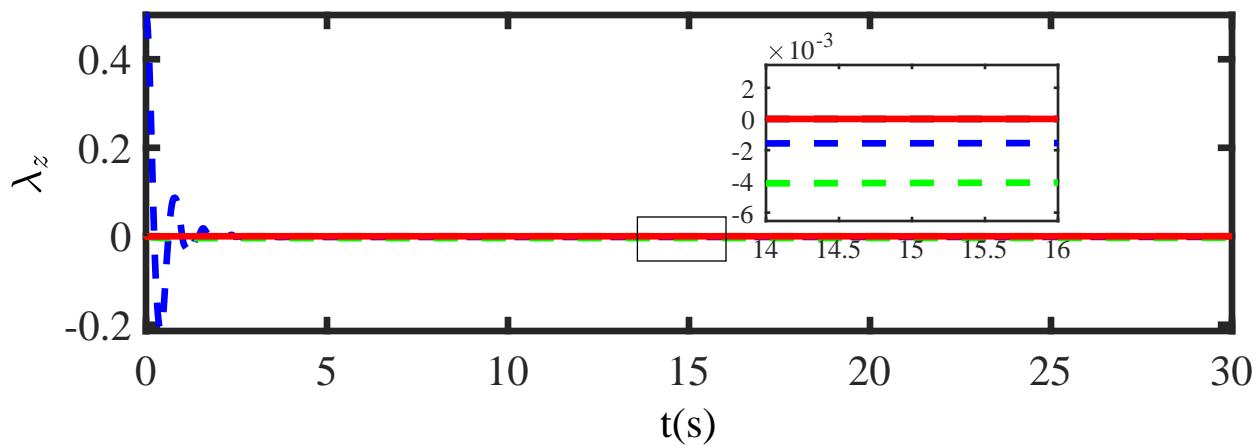

Supplement: S1 File — (ZIP) [file pone.0331662.s001.zip › LaTeX source file (with figures)-bib/Fig8-eps-converted-to.pdf]

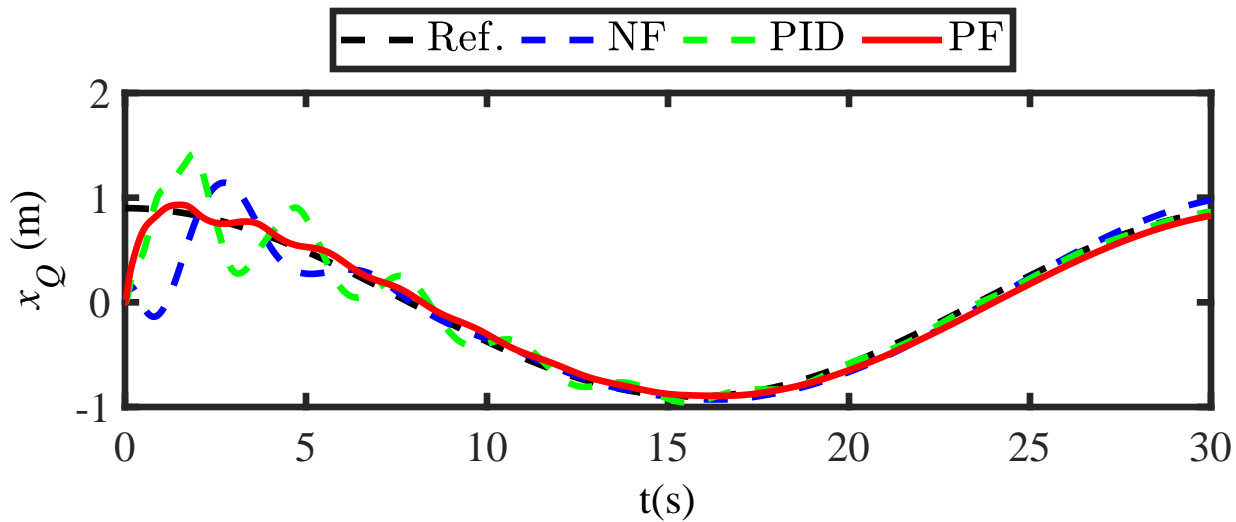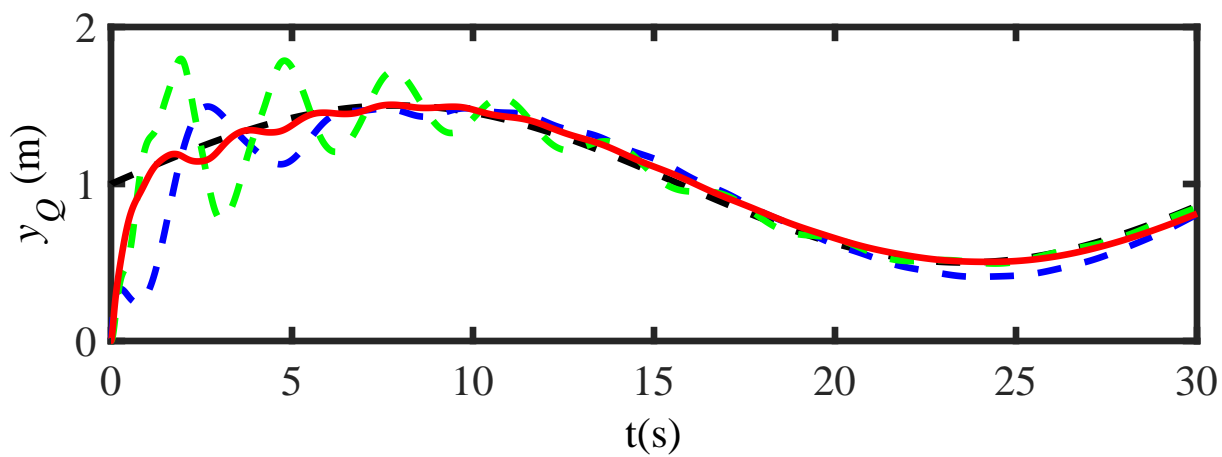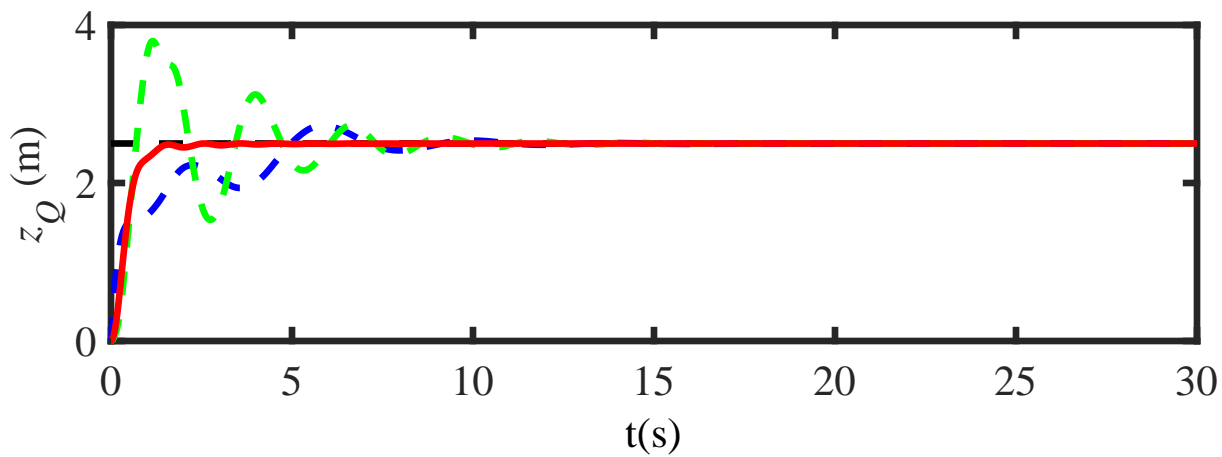

Supplement: S1 File — (ZIP) [file pone.0331662.s001.zip › LaTeX source file (with figures)-bib/Fig9-eps-converted-to.pdf]
