# Supplementary material for: Robust finite-time anti-swing control for quadrotor slung-load system based on compensation function observer
Source: PLoS One. 2026 Apr 24;21(4):e0331662. doi: 10.1371/journal.pone.0331662 (PMC13108781; doi:10.1371/journal.pone.0331662)
Supplement: S1 File — (ZIP) [file Fig1-eps-converted-to.pdf › LaTeX source file (with figures)-bib/Fig1-eps-converted-to.pdf]

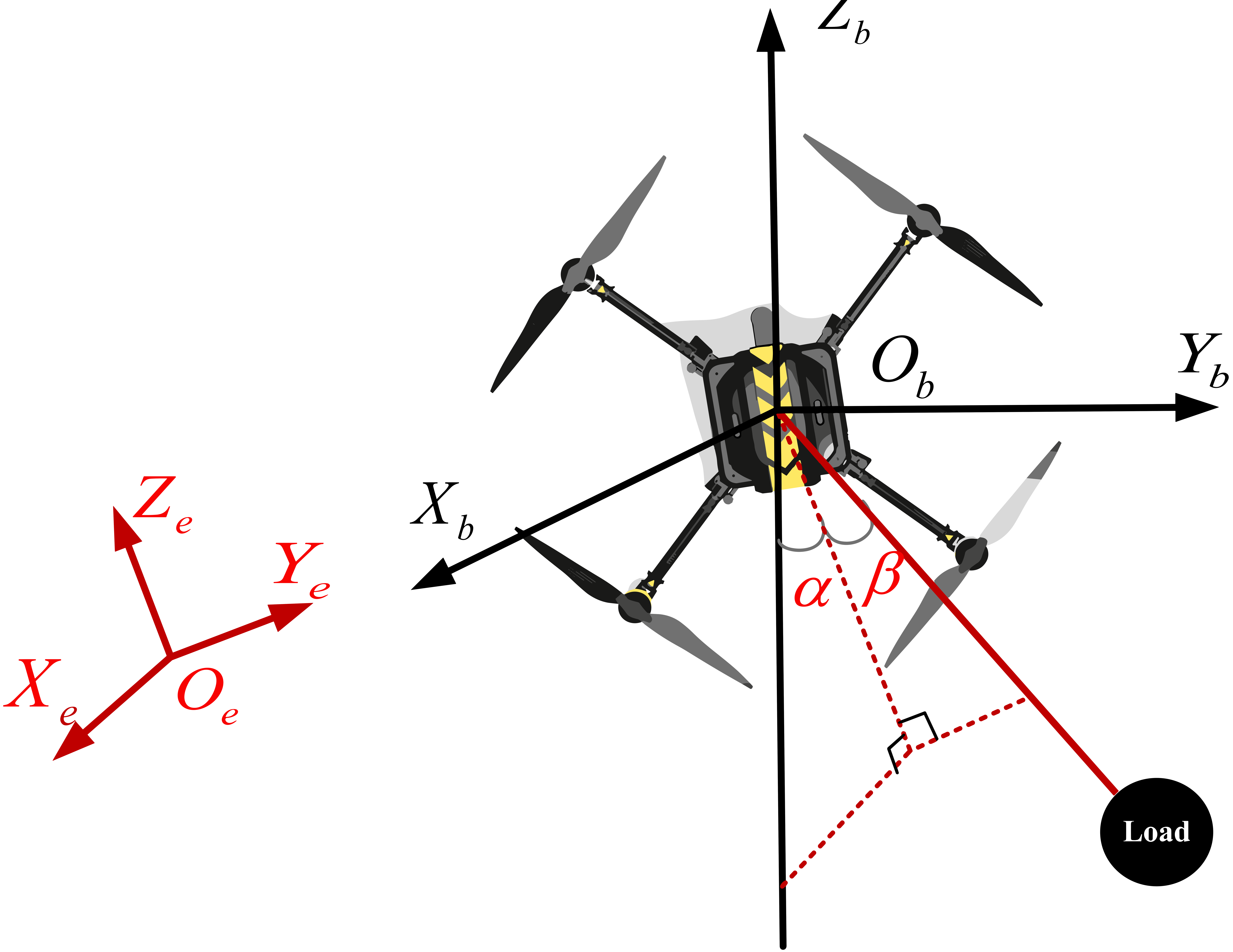

Z_b
O_b
Y_b
X_b
α
β
Load
Z_e
Y_e
X_e
O_e
