# Supplementary material for: Robust finite-time anti-swing control for quadrotor slung-load system based on compensation function observer
Source: PLoS One. 2026 Apr 24;21(4):e0331662. doi: 10.1371/journal.pone.0331662 (PMC13108781; doi:10.1371/journal.pone.0331662)
Supplement: S1 File — (ZIP) [file Fig2-eps-converted-to.pdf › LaTeX source file (with figures)-bib/Fig2-eps-converted-to.pdf]

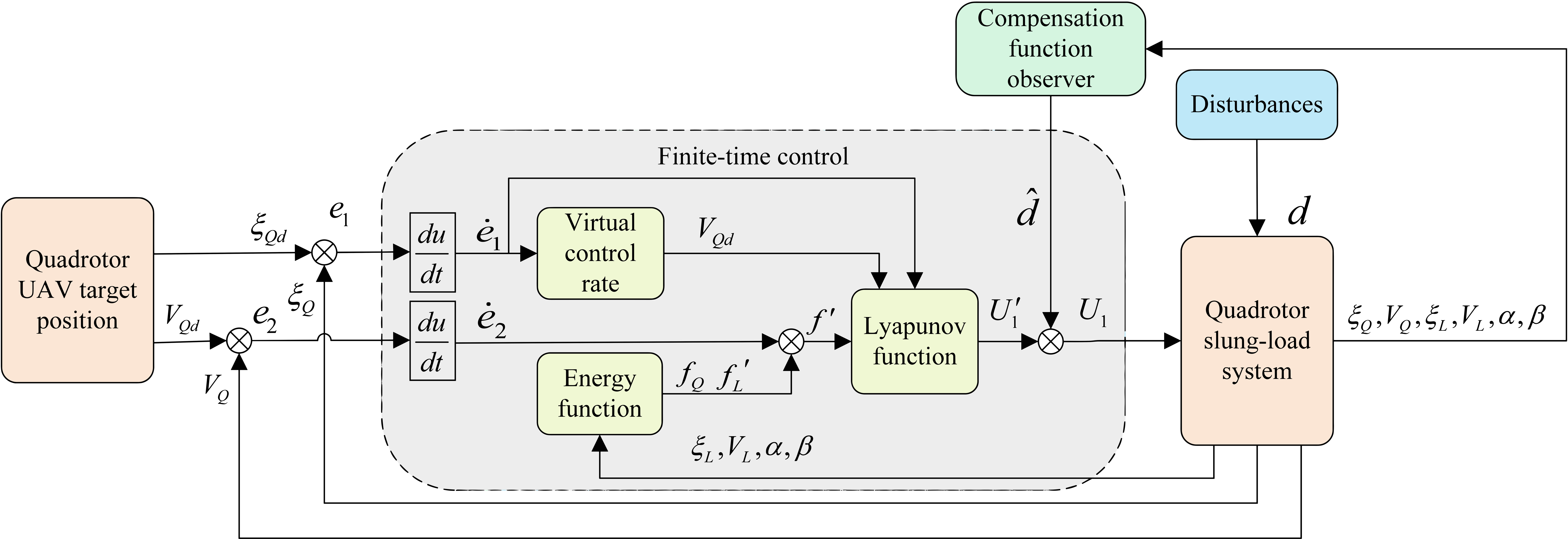

Compensation function observer
Disturbances
Finite-time control
Quadrotor UAV target position
ξ_{Qd}
e_1
V_{Qd}
e_2
ξ_Q
V_Q
$\frac{du}{dt}$
$\dot{e}_1$
$\dot{e}_2$
Virtual control rate
V_{Qd}
$\hat{d}$
d
Energy function
$f_Q \ f_L{}'$
f'
Lyapunov function
U_1'
U_1
Quadrotor slung-load system
$\xi_Q, V_Q, \xi_L, V_L, \alpha, \beta$
$\xi_L, V_L, \alpha, \beta$
